# Prompt-based bioinformatic pipeline generation for a multi-step metaviral workflow

**DOI:** 10.1093/bioadv/vbaf308

**Published:** 2025-11-27

**Authors:** Pengchong Ma, Haoze Zheng, Weijun Yi, Li Ma, Brandi Sigmon, Karrie A Weber, Gangqing Hu, Qiuming Yao

**Affiliations:** School of Computing, University of Nebraska Lincoln, Lincoln, NE 68588, United States; School of Computing, University of Nebraska Lincoln, Lincoln, NE 68588, United States; Department of Microbiology, Immunology & Cell Biology, West Virginia University, Morgantown, WV 26505, United States; Lane Department of Computer Science & Electrical Engineering, West Virginia University, Morgantown, WV 26506, United States; Department of Microbiology, Immunology & Cell Biology, West Virginia University, Morgantown, WV 26505, United States; Department of Plant Pathology, University of Nebraska-Lincoln, Lincoln, NE 68583, United States; School of Biological Sciences, University of Nebraska-Lincoln, Lincoln, NE 68588, United States; Department of Earth and Atmospheric Sciences, University of Nebraska-Lincoln, Lincoln, NE 68588, United States; Daugherty Water for Food Institute, University of Nebraska System, Lincoln, NE 68588, United States; Department of Microbiology, Immunology & Cell Biology, West Virginia University, Morgantown, WV 26505, United States; School of Computing, University of Nebraska Lincoln, Lincoln, NE 68588, United States; Nebraska Center for Virology, University of Nebraska-Lincoln, Lincoln, NE 68583, United States

## Abstract

**Motivation:**

The rapid evolution of bioinformatics tools and multi-step analytic procedure presents a challenge for building effective pipelines, particularly for researchers without extensive programming expertise. This study demonstrates that large language models (LLMs) hold strong potential for generating end-to-end bioinformatic pipelines through carefully crafted prompts, using a multi-step metaviral workflow as a representative example. Multiple LLMs were tested for their effectiveness, including OpenAI ChatGPT series, Anthropic Claude series, Google Gemini, Meta Llama, and DeepSeek.

**Results:**

Our results show that ChatGPT-4, ChatGPT-5, Claude 4.5, and Gemini 2.5 consistently outperform other LLMs in generating complete bioinformatic pipelines, with statistically significant success rates. These models also handle tool substitutions effectively. Simple prompt engineering and the inclusion of official documentation further enhance performance, especially for newer bioinformatic tools. While capabilities vary, all LLMs tested show potential for both pipeline generation and updates with our designed prompts and strategies.

**Availability and implementation:**

All prompts are available in the paper. The examples are available in GitHub https://github.com/mpckkk/pBio.

## 1 Introduction

One of the central responsibilities of bioinformaticians is to design data analysis pipelines tailored to diverse biological questions. However, the rapidly expanding landscape of bioinformatics tools, alongside the continuous emergence of new data types, creates significant and frequent learning curves. Keeping up to date with the latest software packages, methods, and best practices can be overwhelming, even for experienced computational scientists. For experimental biologists and researchers with limited knowledge of technical jargon and programming expertise, constructing effective bioinformatic pipelines becomes even more challenging.

Recent advances in large language models (LLMs), particularly after the public release of ChatGPT in late 2022, have opened new avenues for addressing these challenges, although current technical support applications remain modest (5%–12% among all ChatGPT use cases), highlighting substantial potential for broader increase (Chatterji *et al.* 2025). ChatGPT offers a natural language interface capable of translating analytical goals into executable code, potentially enhancing access to bioinformatics analyses. It facilitates complex workflow design, lowers barriers for non-experts, and accelerates exploratory analysis, especially in time-sensitive biomedical contexts (Shue *et al.* 2023, Xu 2023, Zhou *et al.* 2024). Moreover, it has been extensively applied across diverse biomedical tasks (Wang *et al.* 2024, Xu *et al.* 2025).

One example is Optimization of Prompts Through Iterative Mentoring and Assessment (OPTIMAL), a framework that harnesses ChatGPT to assist users, especially beginners, in generating and refining programming-heavy bioinformatics workflows (Shue *et al.* 2023). Beyond the OPTIMAL framework, several recent studies have demonstrated the utility of ChatGPT and other LLMs in bioinformatics analysis and tool development. The BioCoder benchmark was introduced to assess LLMs capability to produce bioinformatics-specific code. This benchmark encompasses a wide range of tasks, such as sequence analysis, and includes a diverse set of programming challenges (Tang *et al.* 2024). Evaluations using BioCoder revealed that while models like ChatGPT-4 showed promise in bioinformatic algorithms, there remains a gap in generating scripts for complex bioinformatics pipelines. In the realm of single-cell RNA sequencing (scRNA-seq) analysis, the published works using ChatGPT can assist from cell embedding to annotation. GenePT is embedding genes by ChatGPT-3.5 weighted single cell expression data to cluster the cells (Chen and Zou 2025). GPT-4 has been assessed for its capability to accurately annotate cell types, and its generated annotations exhibit strong concordance with manual annotations, potentially reducing the effort and expertise required for cell type annotation(Hou and Ji 2024). These advancements highlight the growing interest in leveraging LLMs to lower the barrier for cutting-edge and complex bioinformatics analyses, making them more accessible to researchers with varying levels of computational expertise and domain knowledge.

Building on this concept, our study explores how prompt-based interactions with LLMs can support the automated generation and continual adaptation of bioinformatic pipelines. These pipelines involve multiple end-to-end steps that require precise command syntax and seamless data input and output flows between tools. Unlike isolated algorithm design, pipeline construction introduces additional complexity due to frequent updates in bioinformatics tools, which make it difficult for both new and experienced users to maintain the valid computing protocol. Although LLMs have been tested on general coding tasks, their ability to generate complete, modular bioinformatic pipelines remains underexplored, especially in dynamic, real-world analysis settings and from the perspective of users with limited computational expertise.

**Figure 1.**
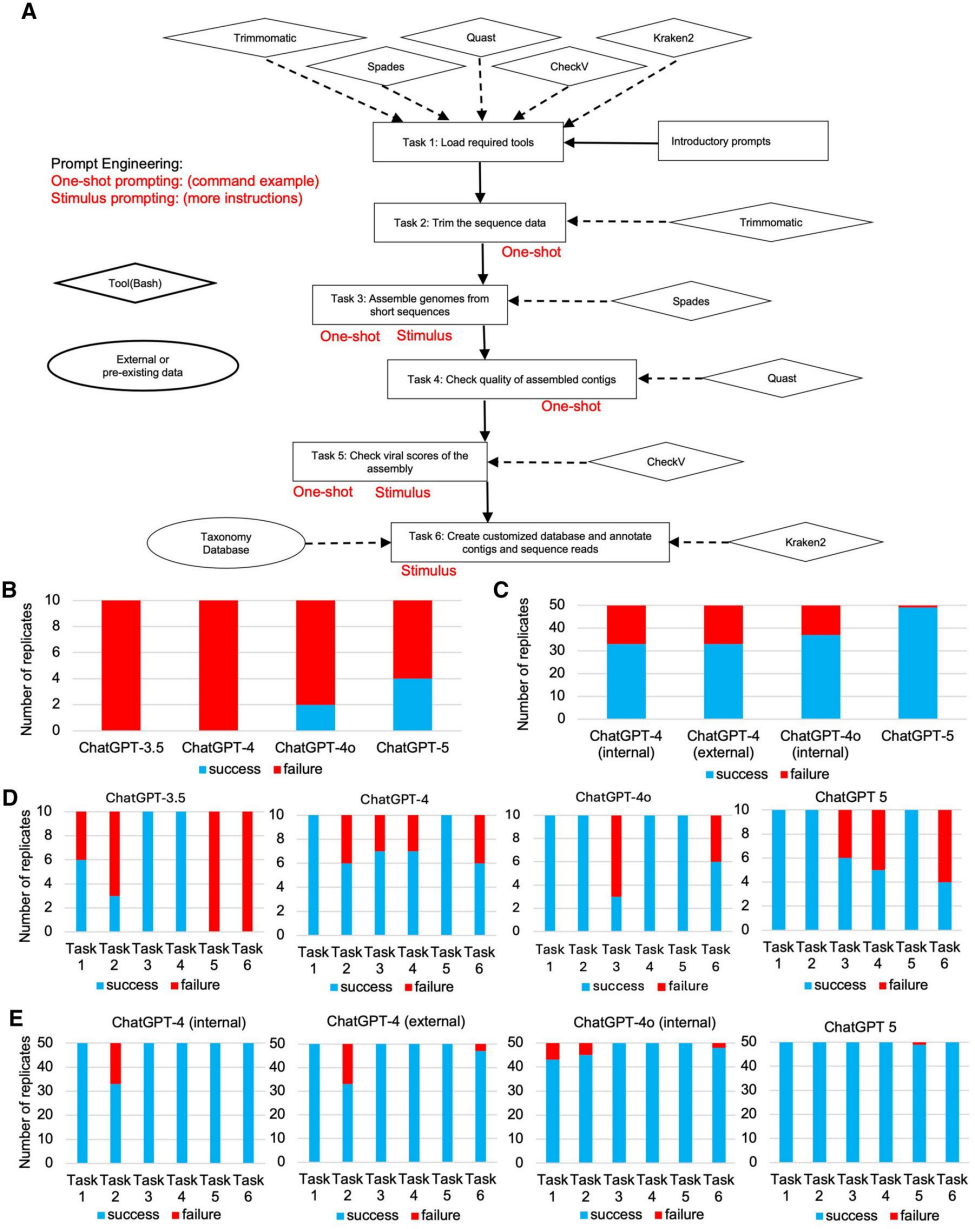
Pipeline building by ChatGPT (A) pipeline diagram, (B) total correctness by natural language version prompts, (C) total correctness by structured prompts, (D) task-wise correctness by natural language version prompts, (E) task-wise correctness by structured prompts.

In this study, to provide a controlled and reproducible testing scenario, we manually constructed a representative metaviral pipeline that reflects common practices in viral genome assembly and annotation. Metaviral analysis plays a critical role in human microbiome and environmental science. This pipeline includes essential tasks (Fig. 1A): loading version-specific tools, trimming paired-end reads, assembling viral genomes, evaluating assembly quality and completeness, and finally building a custom database for annotating both contigs and raw reads. Executing these tasks requires foundational bioinformatics knowledge, including next generation sequencing (NGS) data handling, genome assembly, annotation, and command-line scripting, highlighting the non-trivial nature of the computational protocol. Can a regular user simply explain the above computational needs of this analysis and obtain a sequential set of executable commands? In the following sections, using this minimal metaviral analysis as a case study, we evaluated the effectiveness of a structured prompting design, explored simple prompt optimization techniques, tested the feasibility of tool replacement for evolving methods, and compared output scripts across different LLMs. We aim to offer practical insights into the potential of prompt-based approaches to streamline pipeline development and provide a flexible framework for integrating language models into routine bioinformatics analysis and education.

## 2 Methods

### 2.1 Example data and reference codes

The metaviral analytic dataset we used in this study is a small, simulated dataset with one million paired-end reads by InSilicoSeq (Gourlé *et al.* 2019) from an artificial viral community containing a mixture of single- and double-stranded DNA viruses in 2 × 126 bp format on an Illumina HiSeq mode. The ground-truth reference consists of 12 viral genomes, including 10 double-stranded DNA (dsDNA) viruses and 2 single-stranded DNA (ssDNA) viruses. The dsDNA group includes multiple *Cellulophaga* phages (e.g. *phi13:1*, *phi18_1*, *phi18_3*, *phi38_1*, *phi38_2*) and PSA phages (*HS2*, *HM1*, *HS6*, *HP1*, *HS9*), representing diverse marine bacteriophages. The ssDNA group includes two *Enterobacteria* phages, *phiX174* and *alpha3*. The simulated sequencing data generated from this curated genome collection offer a simplified and verifiable benchmark for evaluating the correctness of metaviral analysis pipelines, which is especially necessary given that real virome datasets often lack definitive ground truth. However, our methodological framework is independent of any specific dataset used, as the focus of this study is not on evaluating the performance of each metaviral tool themselves, but rather on assessing the ability of large language models to generate accurate and executable command-line instructions. This dataset enables straightforward verification of command correctness, as its configuration and expected outputs allow for quick execution and easy validation.

The analytical pipeline reflects typical steps by a routine viral genomics analyst, encompassing both de novo assembly-based and reference-based approaches. The workflow includes loading appropriate tools, trimming raw sequencing reads, assembling viral genomes, assessing the quality of assemblies, and analyzing both contigs and raw reads using a curated mini-reference database (Fig. 1A). While the pipeline involves multiple stages and requires some understanding of the logical sequence of bioinformatics tasks, it remains sufficiently straightforward to allow for evaluation of command correctness. This use case goes beyond the beginner level yet does not demand advanced computational expertise, making it a practical and representative scenario for our study.

The reference codes (Fig. 1, available as supplementary data at *Bioinformatics Advances* online) have been tested well over the simulated dataset, and it can finish within 30 minutes on a basic Linux terminal equipped with a single CPU (Intel Xeon Gold 6348 Processor, 2.6 GHz) and ∼10 GB of memory, without the need for specialized hardware. The input consists of (i) a raw sequencing file (read1.fastq.gz and read2.fastq.gz), (ii) an adapter sequence file (adapters.fa), and (iii) a reference database folder (Ref_database) containing reference genome FASTA files. The expected output includes (i) an assembled contigs file (contigs.fasta), (ii) quality assessment reports for the assembly, and (iii) two annotation reports, i.e. one for annotated contigs and one for annotated filtered reads. The AI-generated bioinformatic codes were executed using the same computational settings and input dataset as the manually curated reference pipeline. Hardware capability in this study however does not affect the efficacy of our testing cases.

### 2.2 Web test for LLMs

**Figure 2.**
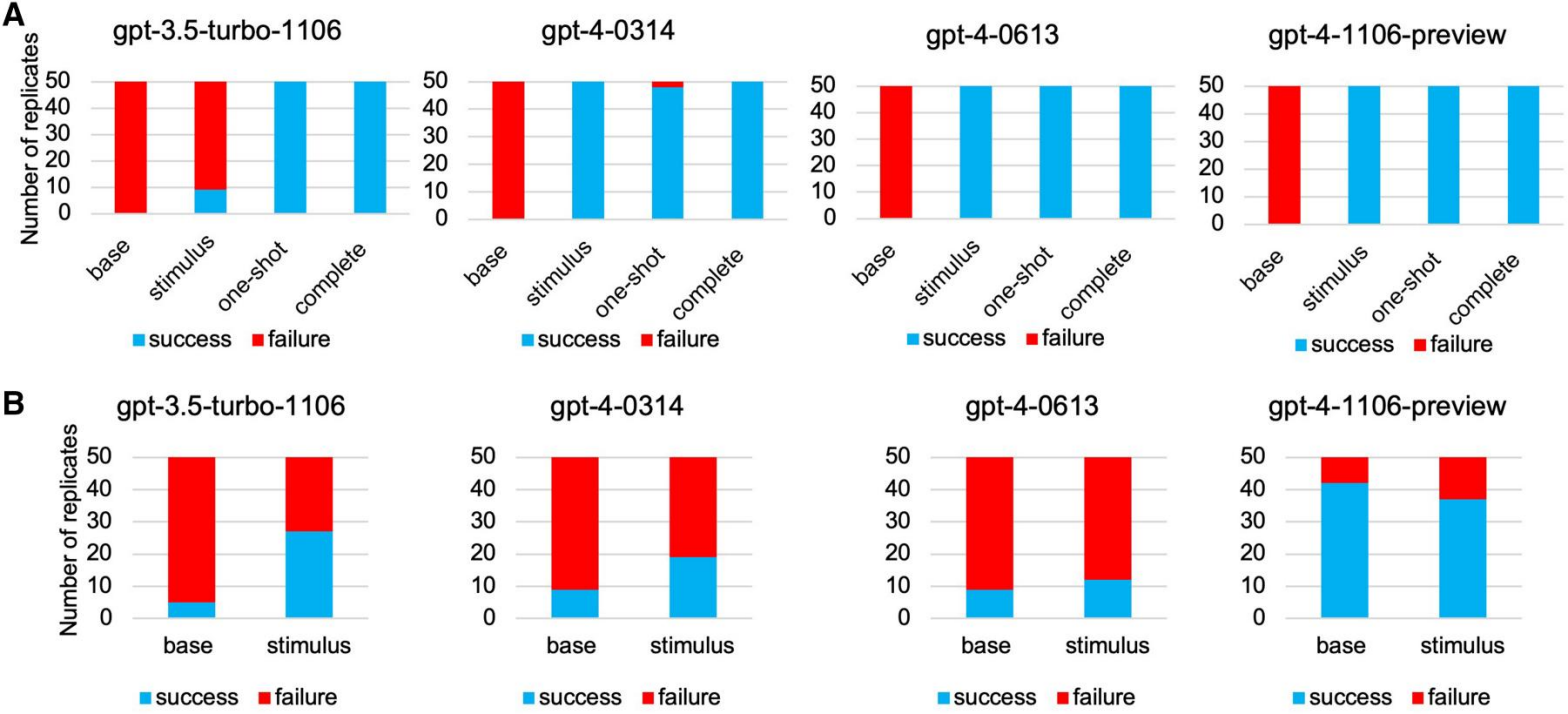
Effect of prompt engineering through ChatGPT API (A) Correctness in Task 3 (B) and Task 6 by the baseline prompts, prompt with stimulus, prompt with one-shot, and the final complete prompt (with both stimulus and one-shot)

Most LLMs offer freely accessible web interfaces that are suitable for regular users. To simulate real-world usage by individuals with limited computational expertise, we tested most of the recent LLMs directly through their web interfaces by our designed prompts (Fig. 2, available as supplementary data at *Bioinformatics Advances* online). We did not modify the default settings for all hidden parameters in each LLM; we documented key model specifications for every tested LLM, including parameter size, context window, model release date, and the date of our testing (Tables 1 and 2, available as supplementary data at *Bioinformatics Advances* online). Prompt testing was conducted on the LLM web server across multiple scenarios. (i) For end-to-end pipeline generation, both natural language and structured prompts were tested in LLMs, and 10 replicates were performed in most cases. Structured prompt testing with ChatGPT-4, ChatGPT-4o, and ChatGPT-5 included 50 replicates each. In our study, a replicate refers to an independent run in which the same pipeline-generation prompt is submitted to the language model, and a complete end-to-end set of pipeline code is generated, allowing us to assess consistency and variability in the model’s responses. To minimize the session effect, all web testing cases and replicates were performed in a separate browser window with memory save option off. (ii) For single viromic tool testing scenarios, six newly developed viral genome classification and annotation tools: VirSorter2 (Guo *et al.* 2021), CheckV (Nayfach *et al.* 2021), vRhyme (Kieft *et al.* 2022), PHAMB (Johansen *et al.* 2022), geNomad (Camargo *et al.* 2024), and iPHoP(Roux *et al.* 2023) (Table 3, available as supplementary data at *Bioinformatics Advances* online) were evaluated with 10 replicates each. (iii) For the tool replacement scenario, we use the reference code as a baseline pipeline and substitute two viral annotation tools to simulate pipeline evolution with newer alternatives. To assess reproducibility and consistency, each substitution test was repeated across 10 replicates. In both viromic tool testing cases and replacing experiments, we applied a strategy of submitting prompts together with tool documentations. We assumed that the cutoff time of the LLMs affect the usage information of the novel tools, where the documentation can help to generate the correct command of the new tools. All documentation was obtained (simply copied) directly from tool official GitHub repositories README file (Table 3, available as supplementary data at *Bioinformatics Advances* online), and submit as a text document together with prompt in ChatGPT-4, ChatGPT-4o, Claude 3.5 Sonnet and further models. Other LLMs may not be able to accept a document or were not tested at the time of writing this manuscript.

In the testing of metaviral pipeline generation, internal testing was conducted by our team at the University of Nebraska-Lincoln, while external testing was performed by collaborators at West Virginia University. External validation was limited to web-based ChatGPT-4 cases, focusing on evaluating prompt usability and effectiveness in generating full pipelines through the ChatGPT interface at a different geographic location, during our early development of the prompts.

### 2.3 API test for ChatGPT prompt engineering

Due to the difficulty of composing effective prompts with earlier ChatGPT models, we focused specifically on prompt engineering in Task 3 (genome assembly) and Task 6 (reference-based annotation) to gain deeper insights. We conducted 50 replicates using the ChatGPT API across four different model versions (gpt-3.5-turbo-1106, gpt-4-0314, gpt-4-0613, gpt-4-1106-preview), where gpt-4-1106-preview is the most recent API model tested. To evaluate the effectiveness of different prompting strategies, we tested four prompt types: base, stimulus, one-shot, and complete. The base prompt contains minimal task instructions and serves as a baseline (e.g. perform genome assembly). The stimulus prompt adds clarifying language to improve task interpretation (e.g. with specific virology focused method). The one-shot prompt includes a simple working example to illustrate the desired command or parameters (e.g. example command is as such). The complete prompt combines both stimulus guidance and a one-shot example, leading toward the most efficacy for the model (Tables 23–24, available as supplementary data at *Bioinformatics Advances* online).

### 2.4 Evaluation metrics

**Figure 3.**
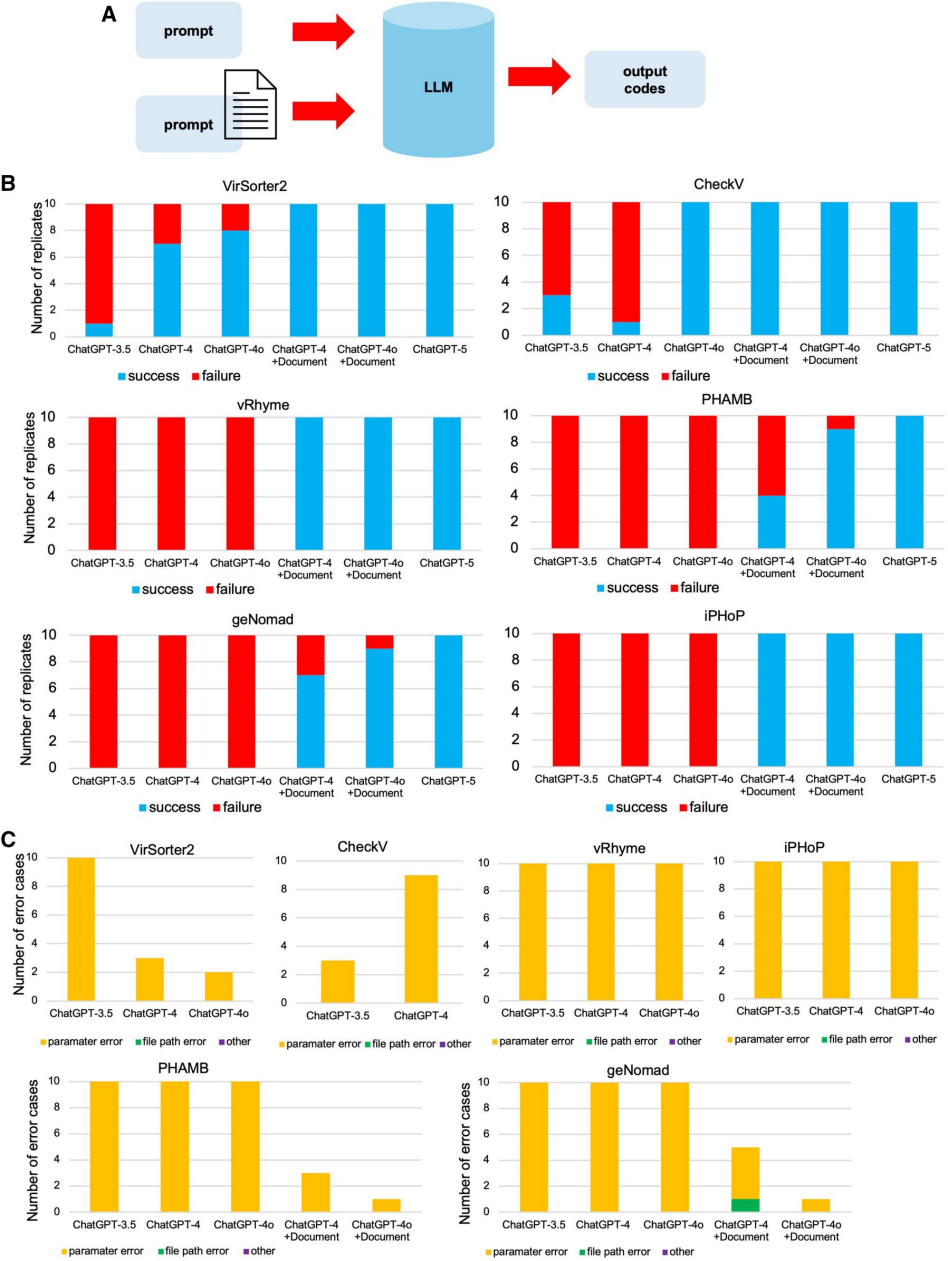
Correctness of the LLM-generated commands on recent tools. (A) Conceptual figure for prompting with and without README document. (B) Correctness for six representative tools among 10 testing cases (C) Error category breakdown for LLM-generated commands across six tools.

For both end-to-end commands script and individual command generated by LLMs, we execute them using simulated data on a minimal Linux environment (as mentioned above) to assess their functionality. If a generated script fails, we manually compare it with the corresponding reference code to identify discrepancies. Successful executions are further validated by examining outputs at multiple checkpoints to confirm result correctness and data consistency (Fig. 3, available as supplementary data at *Bioinformatics Advances* online).

Single command runtime errors are categorized into three types: parameter errors (invalid syntax or unsupported arguments), file path errors (incorrect use of input/output paths), and other errors. Since reference code and command do not cover all scenarios, when we manually characterize the error types, we also refer to official documentation from each tool’s GitHub repository and command help messages. This manual verification checks for proper syntax, required parameters, and appropriate workflow structure.

**Figure 4.**
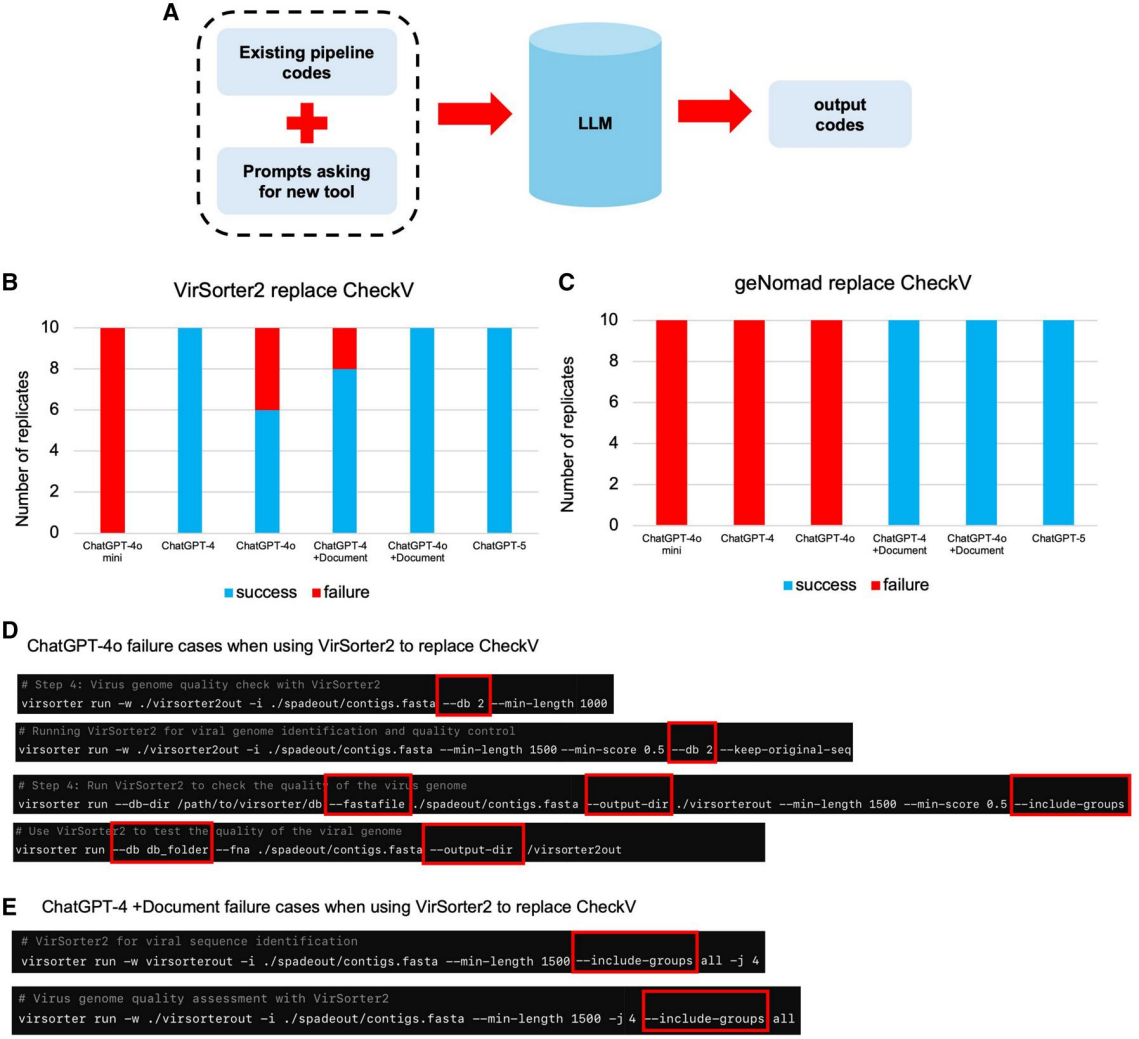
Tool replacement in existing pipeline. (A) Conceptual figure to illustrate the testing idea. (B) Replace CheckV with geNomad in the pipeline. (C) Replace CheckV with Virsorter2 in the pipeline. (D) Four ChatGPT-4o failure cases of VirSorter2 commands in B (E). Two ChatGPT-4o + Document failure cases of VirSorter2 commands in B.

**Figure 5.**
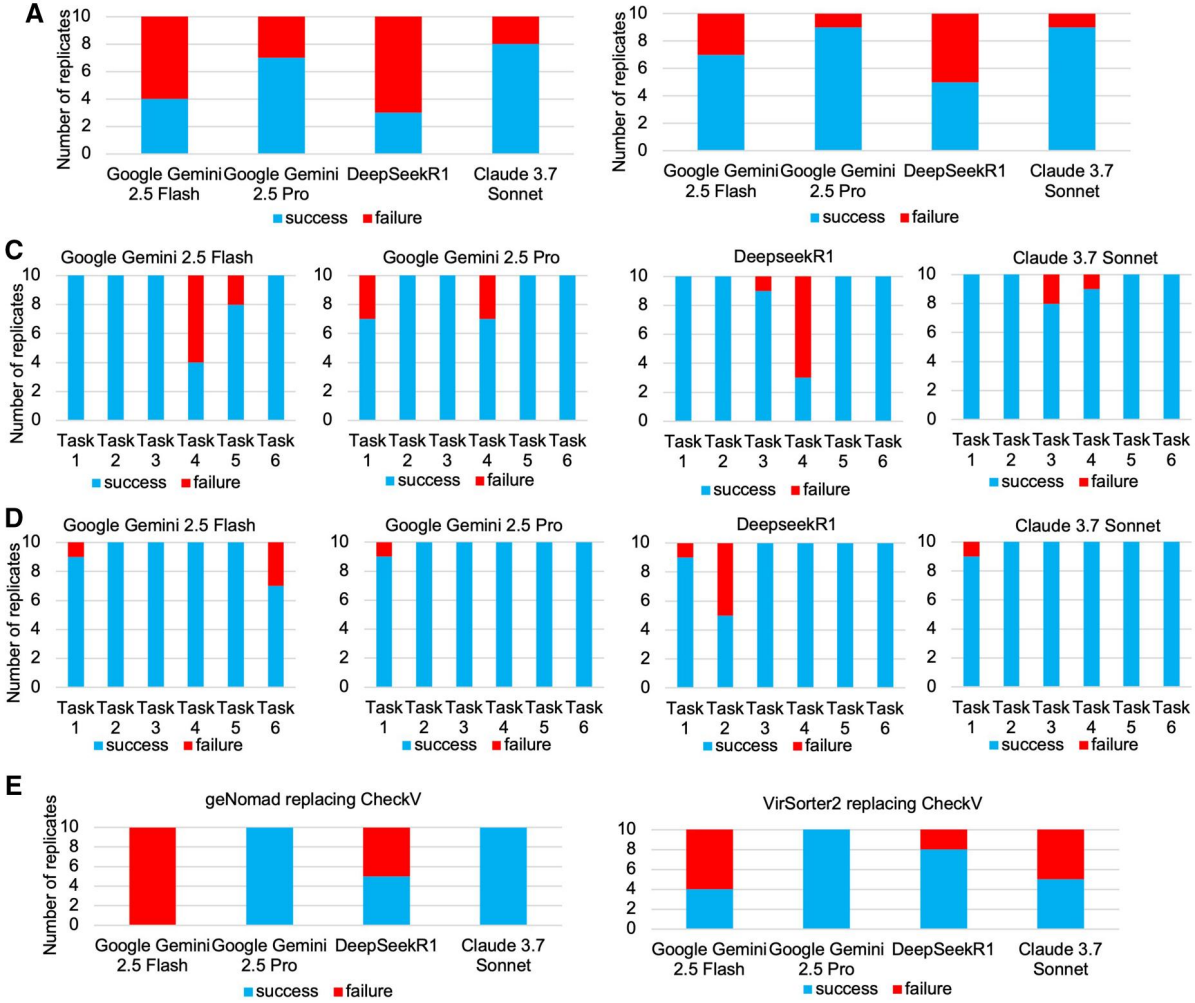
Testing results with other large language models (LLMs). (A) Pipeline generation with natural language prompts (B), structured prompts (C) total correctness and task-wise correctness natural language. (D) Structured prompts (E) tool replacement test in existing pipeline (geNomad replacing CheckV and Virsorter2 replacing CheckV).

**Figure 6.**
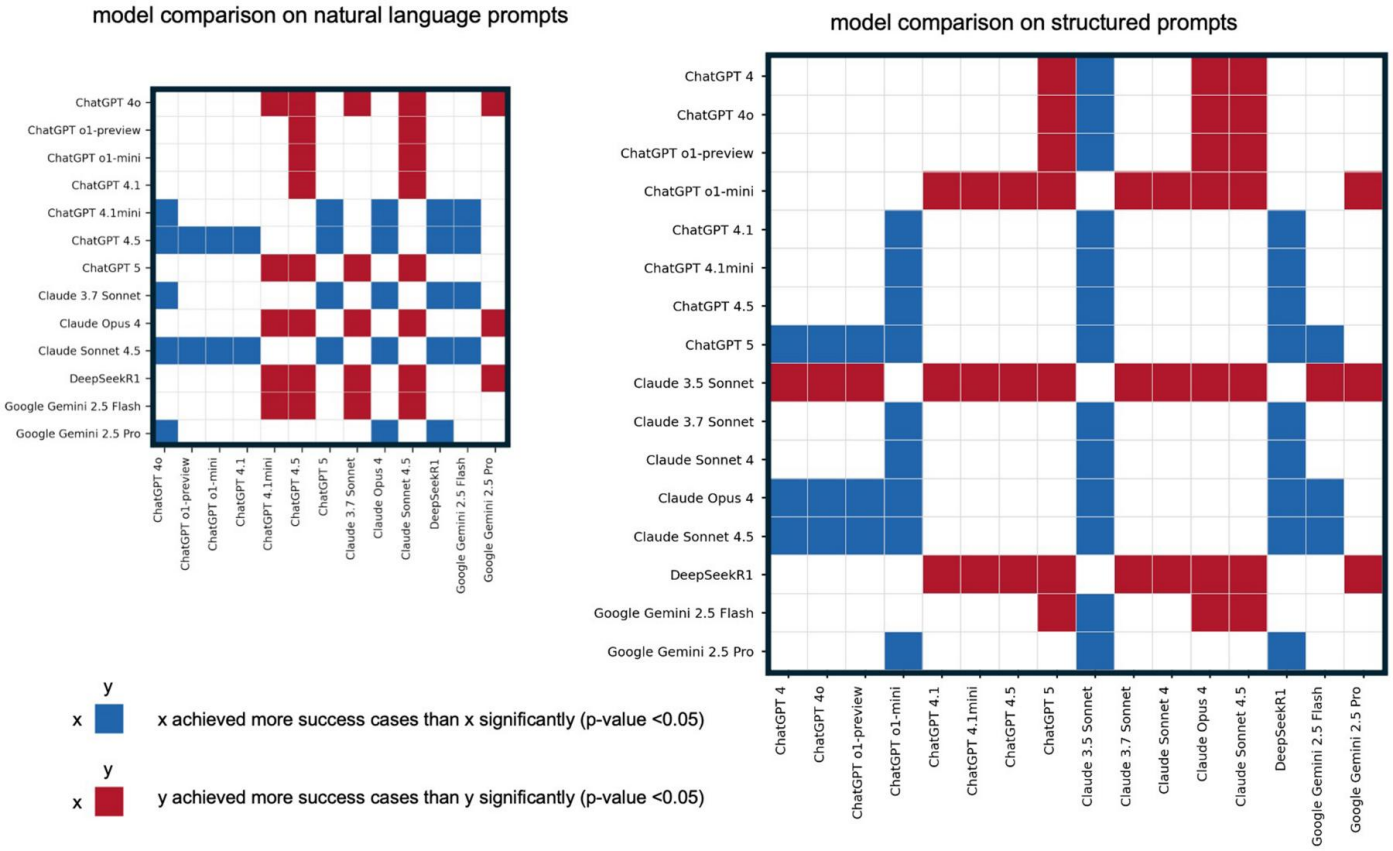
Model performance comparison heatmap showing the significant *P* values (models are tested only when success proportion is non-zero).

For end-to-end pipelines, any failure in a single step results in the whole pipeline being classified as incorrect, although we track which step caused the failure and report typical error cases (Figs 4–8, available as supplementary data at *Bioinformatics Advances* online). When commands are correct, the results are fully reproducible with no random deviations in our analytical procedure. Thus, in our evaluation, we treat command errors and output data errors equivalently when determining overall correctness.

### 2.5 Statistical comparison of LLM performance

To evaluate whether two LLMs differ significantly in their ability to generate correct bioinformatic commands or pipelines, we apply a two-sample Z-test for proportions. This statistical test assesses whether the observed difference in success rates between two independent groups is likely due to chance. Let ${\hat{p}}_{1}$and ${\hat{p}}_{2}$ represent the proportion of correct outputs generated by LLM-1 and LLM-2, respectively, based on $n_{1}$and $n_{2}$ trials. The pooled proportion $\hat{p}$ is calculated as:


$$\hat{p}=\frac{x_{1}+x_{2}}{n_{1}+n_{2}}$$

where $x_{1}$ and $x_{2}$ are the number of successful outcomes for each model. The *Z* statistic is then computed using the formula:


$$Z=\frac{{\hat{p}}_{1}-{\hat{p}}_{2}}{\sqrt{\hat{p}(1-\hat{p})(\frac{1}{n_{1}}+\frac{1}{n_{2}})}}$$

This test evaluates the null hypothesis $H_{0}:p_{1}=p_{2}$ (i.e. both LLMs perform equally well) against the alternative hypothesis $H_{1}:p_{1}\neq p_{2}$. A significant *Z*-score indicates a statistically meaningful difference in performance. A high absolute *Z*-score and a corresponding low *P* value (typically <.05) would lead us to reject the null hypothesis, indicating a statistically significant difference in the performance of the two LLMs.

## 3 Results

### 3.1 Structured prompts outperform fully natural language instructions

Metaviral analyses have enabled detailed characterization of viral communities across diverse environments from water to the human gut. This analytic pipeline includes essential tasks (Fig. 1A) loading version-specific tools, trimming paired-end reads, assembling viral genomes, evaluating assembly quality and completeness, and finally building a custom database for annotating both contigs and raw reads, requiring foundational bioinformatics knowledge. Although minimal in design, this metaviral pipeline effectively integrates both de novo assembly and reference-based approaches, reflecting a realistic case study in viral metagenomics. Each step varies in its complexity; some, like read trimming or quality checks, are relatively straightforward, while others, such as Task 6, involve more intricate operations like building a custom database and performing taxonomic quantification. For each task, we used ChatGPT to generate shell scripts, and evaluate their effectiveness in running them and comparing with reference codes (Fig. 1, available as supplementary data at *Bioinformatics Advances* online). The prompts were manually designed and optimized. Successful execution can be further verified on key checkpoints of the data outputs (Fig. 3, available as supplementary data at *Bioinformatics Advances* online), particularly the quality of genome assembly and the accuracy of downstream reference-based genome quantification.

In the end-to-end pipeline construction, we designed and compared two styles of prompts: a natural language version and a structured version (Fig. 2, available as supplementary data at *Bioinformatics Advances* online). The natural language prompt mimics a typical user scenario, where all requirements are provided in one paragraph with minimal formatting. This version is designed to test the model’s ability to parse free-text instructions and generate a complete, end-to-end pipeline. In contrast, the structured prompt is tailored for users with some bioinformatics and coding background. It breaks the workflow into discrete tasks, each with clearly defined inputs, outputs, tools, and example commands, like writing a computational protocol in each task. In both prompts, we added minimal examples and instructions to make the prompts less confusing to ChatGPT, which will be systematically tested in the next section.

We tested four versions of ChatGPT, ChatGPT-3.5, ChatGPT-4, ChatGPT-4o, and ChatGPT-5 on both prompt designs (Table 1, available as supplementary data at *Bioinformatics Advances* online). In our strict evaluation, any single step failure will considered as the pipeline failure. The natural language version led to high failure rates across all four models, with the majority of tasks incorrectly executed (Fig. 1B). By contrast, structured prompting significantly improves success rates, particularly for ChatGPT-5 (Fig. 1C) (GPT-3.5 was no longer available when the time we developed structured prompt). The external test at a different location from our collaborative lab shows the performance stableness with the same prompt. This reflects the design of the prompts is the key to LLMs for complex bioinformatics workflows.

Task-specific breakdown of performance for the natural language prompt (Fig. 1D) shows that ChatGPT-3.5, ChatGPT-4, and ChatGPT-5 struggled across nearly all tasks, especially Tasks 2 (pre-processing), 3 (genome assembly), 4 (quality checking), and 6 (taxonomy annotation), which involve multiple command-line flags/syntaxes, file re-naming, conditional logics. ChatGPT-4o and ChatGPT-5 show improvement from older versions, but still exhibit confusion in Task 3, Task 4, and Task 6 although we have added examples and instructions in the natural language prompt.

Task-specific performance for the structured prompt (Fig. 1E) shows that, 90%–100% success rates can be achieved across all tasks (except task 2 in GPT-4 and task 1 in ChatGPT-4o). The previous challenges with Tasks 3, 4, and 6 observed in natural language prompt are largely resolved, demonstrating that structured prompts help the models better interpret and execute more complex, multi-step commands.

In summary, in ChatGPT versions, ChatGPT-4o and ChatGPT-5 achieves the highest overall accuracy and generalizability (more models will be mentioned in later sections). The results suggest that prompt engineering, particularly structured decomposition of tasks, is essential for leveraging LLMs in complex bioinformatics analysis and pipeline generation. Our method of designing prompt by input, output, tool specification should be working and practical approach for building pipelines with LLMs.

### 3.2 Simple prompt engineering techniques improve performance

Several lightweight techniques have been developed to refine prompts and elicit more human-like performance from LLMs. Prompt engineering, boosts model accuracy by optimally selecting or tuning prompt examples, ranging from zero-shot (Sivarajkumar and Wang 2022) and few-shot (Brown *et al.* 2020, Lu *et al.* 2023, Chen *et al.* 2024, Šuster *et al.* 2024) to Chain-of-Thought prompting (Wei *et al.* 2022, Hebenstreit *et al.* 2024, Shah and Househ 2025).

In the early test of our metaviral pipeline generation, we observed that large language models like ChatGPT-3.5 and ChatGPT-4 often struggle with complex tasks, particularly those involving nested commands, tool parameters, or multi-step logic, leading to hallucinated or syntactically incorrect code (Figs 4–6, available as supplementary data at *Bioinformatics Advances* online). This was especially evident in Tasks 3 (de novo assembly) and 6 (reference-based annotation), where even advanced versions of ChatGPT frequently failed to correctly construct working commands or appropriately loop through reference files.

To assess the impact of simple prompt engineering techniques, we applied structured prompting in four distinct modes: base (minimal instruction), stimulus (additional instructional cues), one-shot (a single working example), and complete (combination of stimulus and example). We tested these across multiple versions of ChatGPT using the API, with 50 replicates per condition.

Across all models, Task 3 performance significantly improved when stimulus and one-shot techniques were used (Fig. 2A) to emphasize the use of metaviral mode of the assembly tool. Specifically, ChatGPT-3.5, which failed most frequently under the base prompt, reached full success with the complete prompt. ChatGPT-4 and ChatGPT-4o also exhibited 96%–100% success rate under one-shot and complete prompts, indicating that even a single example drastically improves the model’s ability to construct the correct assembly command.

Task 6, which involves Kraken2 (Wood *et al.* 2019) database construction and annotation, is a complex, multi-step task that requires both iterative logic (e.g. file loops) and precise parameter specification. Stimulus prompts led to modest improvements across models (Fig. 2B), but performance remained lower compared to simpler tasks such as Task 3. GPT-3.5-turbo improved from 10% success (base) to 54% success (stimulus). GPT-4-0314 and GPT-4-0613 increased from 18% and 18% (base) to 38% and 24% (stimulus), respectively. The most recent model, GPT-4-1106-preview, showed the highest reliability with 84% success (base) and 74% success (stimulus), maintaining strong performance even with added prompt complexity.

Overall, these results show that simple prompt engineering methods, particularly stimulus instructions and one-shot examples, significantly enhance the accuracy and reliability of LLM-generated codes for our metaviral pipeline. Therefore, later we have added other stimuls and one-shot examples in the other steps wherever needed.

### 3.3 Pre-trained language models capture recent tool developments

To assess whether public LLMs like ChatGPT can effectively capture and apply recent developments in computational virology, we evaluated their ability to generate functional code for six widely used viral analysis tools: VirSorter2 (Guo *et al.* 2021), CheckV (Nayfach *et al.* 2021), vRhyme (Kieft *et al.* 2022), PHAMB (Johansen *et al.* 2022), geNomad (Camargo *et al.* 2024), and iPHoP (Roux *et al.* 2023) (Table 3, available as supplementary data at *Bioinformatics Advances* online). They are across different functionalities from genome binning to annotation. These tools span a release window from 2021 to 2024, allowing us to assess how well ChatGPT models have learned information and usage patterns across time (Fig. 3A). These tools were not explicitly included in our earlier metaviral pipeline test (except CheckV), making them ideal candidates for evaluating the generalizability and temporal learning capability of LLMs.

ChatGPT-3.5 struggled across all tools (Fig. 3B), with high failure rates and limited ability to produce executable commands. ChatGPT-4 demonstrated some improvement, especially for older and more established tools (from 2021) like VirSorter2 and CheckV, which were successfully run in most trials. ChatGPT-4o further enhanced success rates, especially for newer tools, but certain recent packages (2022–2024, newer relative to ChatGPT-4 models), such as geNomad, PHAMB, and iPHoP, remained challenging.

To determine whether the models could overcome these limitations with additional context, we tested the same tasks while supplementing the prompt with relevant GitHub or README documentation (Fig. 3A). The results (Fig. 3B) show a clear improvement for both ChatGPT-4 and ChatGPT-4o, which achieved near-perfect success across all tools when documentation was provided. This highlights that even for newly released tools, which may not have been extensively represented in the training data, ChatGPT can generalize tool usage when given sufficient instructional material. ChatGPT-5 has a later release date and it is already good for testing these tools without feeding extra information. By manually inspection, the errors are almost always syntax error (may indicate hallucination) instead of filenames in these testing cases (Fig. 3C).

These findings suggest that while LLMs can capture general usage patterns of popular bioinformatics tools, especially those with longer public exposure, their ability to apply newly released tools improves substantially when provided with documentation (without any human selection bias of the contents though). Thus, combining LLMs with external knowledge sources is a practical strategy for enabling up-to-date tool usage in bioinformatics workflows.

### 3.4 Tool replacement experiments for pipeline evolution

In real-world bioinformatics, pipelines must often evolve to incorporate newer tools, or updated versions of the algorithms. This adaptability is especially critical in fast-moving fields like viral metagenomics, where new annotation tools are frequently released. To explore whether ChatGPT can support such evolution, we tested their ability to replace an existing component of a pipeline, CheckV (a viral genome quality assessment tool), with two newer alternatives, VirSorter2 and geNomad which are developed more recently.

We designed prompts that combine existing pipeline code with a request to replace CheckV with one of the newer tools. We evaluated multiple versions of ChatGPT (ChatGPT-4o-mini, ChatGPT-4, ChatGPT-4o, ChatGPT-5) with and without supporting documentation. When asked to replace CheckV with geNomad, ChatGPTs struggled more to generate correct commands (Fig. 4B and C) with geNomad than with CheckV, since geNomad is a relatively newer tool. ChatGPT-4o in particular showed confusion around many command syntaxes (Fig. 4D and E). However, when documentation (with GitHub README information) was included in the prompt, performance improved markedly, with ChatGPT-4o successfully completing all tasks. ChatGPT-5 does not have issues in this experiment with our limited testing cases.

Overall, these results demonstrate that while LLMs are capable of updating and evolving bioinformatics pipelines, their success in tool replacement depends on the complexity and specificity of the new tool’s interface. Confusion tends to arise when multiple parameters, configurations, or sub-commands are involved, as seen with VirSorter2 (even with documentation). Supplementing prompts with official documentation remains an effective strategy for overcoming these limitations and enabling pipeline evolution through LLMs.

### 3.5 Performance continuity of ChatGPTs between ChatGPT-4 and ChatGPT-5

To further investigate the progression in performance across the ChatGPT family, we evaluated newer and experimental models (release dates between ChatGPT-4 and ChatGPT-5) including ChatGPT-o1-mini, ChatGPT-o1-preview, ChatGPT-4.1, ChatGPT-4.1-mini, ChatGPT-4.5, and ChatGPT-5 (Tables 1 and 2, available as supplementary data at *Bioinformatics Advances* online). These models were tested using the same metaviral pipeline tasks, under both natural language and structured prompting conditions, and in tool replacement scenarios.

ChatGPT-o1-mini and o1-preview demonstrated limited effectiveness (above 50%) when generating complete pipelines using natural language prompts (Fig. 9, available as supplementary data at *Bioinformatics Advances* online). While o1-preview slightly outperformed o1-mini, both models had high failure rates, even under structured prompts. Both models struggled to substitute CheckV with either VirSorter2 or geNomad, showing that minimal improvements occurred even when the prompt was augmented with contextual changes. In contrast, ChatGPT-4.1-mini and ChatGPT-4.5 achieved 80%–90% total success in pipeline generation with natural language prompts, while ChatGPT-4.1 is slightly behind (Fig. 10, available as supplementary data at *Bioinformatics Advances* online). Task-specific accuracy remained consistently high for ChatGPT-4.1 and ChatGPT-4.5 across all six steps, with the most notable drop only in Task 6 under complex integration requirements. Testing cases with structured prompts did show significant improvement of the success rate among all models. Besides, tool replacement performance was also improved in later ChatGPT models, particularly for VirSorter2, while geNomad remained more challenging, reflecting broader trends observed across models.

Overall, ChatGPT models after ChatGPT-4o show improvements in end-to-end pipeline generation with structured prompts, achieving ∼90% success rates. While structured prompting continues to yield better accuracy, the enhanced natural language understanding of these models allows for more intuitive interactions. ChatGPT-4.5 achieved best results by using natural language prompts. Our designed prompts should have a long continuous value in future.

### 3.6 Testing with alternative and emerging large language models in 2025

Besides very recent ChatGPT-5, there are many emerging LLMs in 2025 from other companies, including DeepSeek R1, Claude 3.7 Sonnet, Claude 4 Sonnet, Claude 4 Opus, ChatGPT-4.5, ChatGPT-5, Google Gemini 2.5 Flash, and Google Gemini 2.5 Pro using the same metaviral pipeline generation and tool replacement prompts (Fig. 5; Figs 11–14, and Table 2, available as supplementary data at *Bioinformatics Advances* online). We have also tested and compared with the alternative models before 2025, such as Gemini 1.5 Flash and Meta Llama 3, Claude 3.5 (Fig. 11, available as supplementary data at *Bioinformatics Advances* online). These evaluations provide insights into how new releases compare in both end-to-end pipeline generation and specific tool usages.

In the end-to-end pipeline generation, natural language prompt still encounter more challenges than structured prompts. DeepSeek R1 showed only moderate performance around 30%–50%, which was comparable to earlier models like ChatGPT-3.5 or Claude 3.5, with frequent failures across complex steps such as read trimming, assembly, and annotation. Claude 3.7 Sonnet, however, displayed meaningful improvements. It achieved better consistency across all six tasks, with fewer failures than its predecessor Claude 3.5, particularly in handling multi-step operations like genome quality assessment and file looping. In full pipeline generation and structured prompt tests, Claude 3.7 was more robust, and its performance in tool replacement tasks was also more reliable, especially when using geNomad as a substitute for CheckV.

Beyond Claude 3.7, we also compared Claude 4 Sonnet, Claude 4 Opus, and Claude Sonnet 4.5. While Claude 4 Sonnet had a higher failure rate in end-to-end pipeline generation, Claude 4 Opus showed slightly improved robustness, failing in fewer individual tasks overall, indicating its potential for pipeline generation. However, its overall task success remained moderate, suggesting that while Claude Opus 4 may be more consistent than earlier Claude models, it has not been better than Claude 3.7 in natural language prompts. Until recently, Claude Sonnet 4.5 shows the most satisfactory performance in both types of prompts, although Claude 3.7 is very acceptable with 80%–90% of the successful rate.

Even though Google Gemini 2.5 Flash and Google Gemini 2.5 Pro came out in late 2025, their performance showed improvement compared with Gemini 1.5 models. One interesting finding is Google Gemini 2.5 Flash shows better accuracy than Google Gemini 2.5 Pro.

We also tested whether these LLMs could update existing pipelines by replacing CheckV with VirSorter2 or geNomad (Fig. 5E). Without documentation, Claude 4.5 Sonnet and Gemini 2.5 pro correctly integrated VirSorter2 and geNomad into the pipeline without any supporting documentation. The earlier LLMs did not perform well in tool replacement.

These results suggest that while non-ChatGPT LLMs are capable of executing our metaviral tasks, recent models perform consistently well in our designed prompts. The overall findings reinforce the critical role of both prompt engineering and external knowledge support in leveraging LLMs for reliable bioinformatics workflow generation and evolution.

### 3.7 Statistic tests among large language models recommend ChatGPT-4.5, ChatGPT-5, and Claude-4.5

To identify which language models perform significantly better in generating end-to-end metaviral pipelines from natural language and structured prompts, we applied a two-proportion z-test using the number of successful trials versus total trials for each LLM. The resulting heatmap summarizes all pairwise comparisons among models with non-zero success rates. Colored cells indicate statistically significant differences (*P* < .05): blue indicates that the model in the row outperforms the model in the column, while red indicates the opposite (Fig. 6; Fig. 15, available as supplementary data at *Bioinformatics Advances* online).

Under natural language prompts, ChatGPT-4.5 and Claude-4.5 outperformed most other models with extremely low *P* values (as small as 8.2 × 10^−4^), whereas ChatGPT-5 did not show a significant advantage. In contrast, under structured prompts, ChatGPT-5 exhibited the strongest gains, particularly compared to other ChatGPT variants (*P* ≈ 10^−6^–10^−9^), together with Claude-4 and Claude-4.5. Due to marginally small testing cases in a lot of models, there is potential lack of statistical power, but our evaluation focuses on overall performance patterns across models, as visualized in the heatmap by comparing row-wise and column-wise trends. Using natural language prompts, ChatGPT-4.5 and Claude-4.5 perform similarly, while using structured prompts, ChatGPT-5, Claude Opus 4 and Claude Sonnet 4.5 achieve comparable good performance. Based on these findings, we recommend Claude-4.5 as a strong general-purpose model, ChatGPT-4.5 for natural language prompts, and ChatGPT-5 for structured prompt applications.

## 4 Discussion

Our study highlights the best practice and potentials of LLMs in supporting bioinformatics pipeline generation, using the context of a minimal viral metagenomics study. While LLMs have demonstrated promising capabilities, their effectiveness is highly dependent on prompt design, user intent, and the evolving nature of bioinformatics tools. Based on our evaluations and observations, we offer several key insights below.

First, structured prompts represent a practical and effective strategy when working with LLMs in computational biology. Rather than relying on purely natural language instructions, we advocate for a medium-expertise approach with a structured format. Prompts designed with our proposed structured format create a balance between clarity and convenience, preserving the human readability of natural language while incorporating structured task elements similar to protocol-like steps. This approach proved to be both reproducible and adaptable across different LLMs and pipeline scenarios.

Second, our results show that simple prompting techniques, especially those incorporating examples, significantly improve the correctness of LLM-generated code, particularly for complex tasks that frequently lead to hallucinated or misconfigured commands. Techniques like stimulus prompts (instructional points) and one-shot examples (minimal working examples) are essential in guiding the LLMs toward correct tool usage, especially in steps that involve non-trivial parameter settings or complex dependencies.

Third, the inclusion of tool documentation, such as GitHub README files or command-line references, substantially improves model performance, particularly for newly released or less commonly used tools. This encourages the value of writing clear, comprehensive, and machine-interpretable documentation during tool development. Not only does this benefit human users, but it also enhances the utility of LLMs in automated code generation and tool integration, allowing broader access to cutting-edge methods. Recently in May 2025, OpenAI released Codex, a new agent product capable of reading and interpreting GitHub repositories directly to support coding tasks, potentially offering additional advantages for applications like bioinformatics pipeline generation and tool integration.

Lastly, our findings support the role of LLMs in pipeline evolution. Beyond de novo pipeline creation, LLMs are increasingly capable of updating individual components to reflect new analytical trends or software updates. This modular adaptability is crucial in real-world settings, where tool replacement, version upgrades, or task-specific customizations are common. With appropriate guidance, LLMs can facilitate this continuous adaptation, making them useful not only as initial code generators but also as collaborative assistants in long-term workflow maintenance.

Although it is impossible to test all LLMs from the past to the future, based on our aggregation of data presented in Poe’s “Early 2025 AI Ecosystem Trends” report, the LLMs we tested collectively account for ∼84.4% of the models most commonly used by users.

In practical scenarios, our code generation workflow is designed to be accessible to real-world users by relying only on free, web-based LLM interfaces, making it feasible for individuals without advanced computational backgrounds. Most academic institutions provide access to Linux environments where users can execute command-line code with minimal software configuration. While the installation of bioinformatics tools is often handled by institutional IT staff, the challenge lies in how to correctly integrate these tools into meaningful analytical workflows, where our approach addresses by enabling LLMs to generate personalized, task-specific pipelines. From our testing, many modern LLMs produce fully functional code in over 50% of cases, and code errors can often be corrected by re-generating the prompt, reducing the burden on users. We emphasize that the accuracy of biological results ultimately depends on the input data and underlying tools used; evaluating the biological correctness of outputs is beyond the scope of this work.

Several recent efforts (Sänger *et al.* 2024, Clarke *et al.* 2025) have applied LLMs to generate scientific workflows by utilizing intermediate, workflow-specific languages that formalize procedural structure. These methods are producing standardized representations required by middleware tools, which serve as interpreters to translate workflows into executable instances. In contrast, our work uniquely emphasizes direct, prompt-based generation of runnable code in an end-to-end fashion, bypassing the need for intermediate interpreters or dialogue-based interaction. While workflow-specific middleware tools offer a more professional pipeline solution, our work enables a more natural and naive case from average users’ intent to conduct bioinformatic analyses.

Although developing AI agents is not the primary goal of this study, we included preliminary tests of coding-specific LLM agents to explore their potential, specifically Cursor and Replit, with results presented (Figs 16 and 17, available as supplementary data at *Bioinformatics Advances* online). While an effective evaluation of agent performance by continuous user interactions is a challenging scope, we believe these modern agentic systems represent an important direction for the next generation of LLM-based tools. They demonstrated robust capabilities in code generation, web search integration, and multi-step workflow automation.

## 5 Conclusion

Taken together, these observations provide a roadmap for leveraging LLMs in bioinformatics, through well-structured, example-enriched prompts; comprehensive documentation practices; and iterative integration into evolving analytical ecosystems. As LLM capabilities continue to improve, so too will their potential to democratize and accelerate computational biology. This study provides practical insights into harnessing LLMs for bioinformatics automation and outlines strategies to improve their reliability and utility in computational biology workflows.

## Supplementary Material

vbaf308_Supplementary_Data

## Data Availability

The example data and reference code can be download from GitHub https://github.com/mpckkk/pBio. The performance data are summarized in Tables 1 and 2, available as supplementary data at *Bioinformatics Advances* online. All ChatGPT series sessions are available publicly and listed in Tables 4–48, available as supplementary data at *Bioinformatics Advances* online.
