# Supplementary material for: Prompt-based bioinformatic pipeline generation for a multi-step metaviral workflow
Source: Bioinform Adv. 2025 Nov 27;6(1):vbaf308. doi: 10.1093/bioadv/vbaf308 (PMC12782108; doi:10.1093/bioadv/vbaf308)
Supplement: vbaf308_Supplementary_Data [file vbaf308_supplementary_data.zip › Supplementary_Tables_v7.pdf]

Supplementary Tables

Supplementary Table 1. Testing summary for web based ChatGPT versions

LLMs	Release	Est Parameter size	Context window size	Test dates	Pipeline test (natural language prompt) (success/total)	Pipeline test (structured prompt) (success/total)	Tool replacement geNomad (success/total)	Tool replacement VirSorter2 (success/total)
ChatGPT 3.5	11/30/2022	175 billion	16,385 tokens	05/2023	0/10	n/a (*)	n/a	n/a
ChatGPT 4	03/14/2023	1.76 trillion	128,000 tokens	9/2023	0/10	33/50 (*)	0/10<br>8/10	10/10<br>8/10
ChatGPT 4 (external)	03/14/2023	1.76 trillion	128,000 tokens	9/2023	n/a	33/50	n/a	n/a
ChatGPT 4o	05/13/2024	200 billion	128,000 tokens	08/2024	2/10	37/50	0/10<br>10/10	6/10<br>10/10
ChatGPT 4omini	07/10/2024	8 billion	128,000 tokens	09/2024	n/a	n/a	0/10	0/10
ChatGPT o1-preview	09/12/2024	300 billion	128,000 tokens	09/2024	5/10	7/10	0/10	10/10
ChatGPT o1-mini	09/12/2024	200 billion	128,000 tokens	09/2024	5/10	5/10	0/10	0/10
ChatGPT 4.1	04/14/2025	31 billion	1,047,576 tokens	05/2025	5/10	9/10	3/10	2/10
ChatGPT 4.1mini	04/14/2025	27 billion	1,047,576 tokens	05/2025	8/10	9/10	0/10	5/10
ChatGPT 4.5	02/27/2025	5~7 trillion	128,000 tokens	05/2025	9/10	9/10	10/10	8/10
ChatGPT 5	08/07/2025	n/a	400,000 tokens	09/2025	4/10	49/50	10/10	10/10

Note: ChatGPT 4 and ChatGPT 4o in tool replacement has two testing case, first row is without documentation, second row is with documentation.
(*)we have tested on their API version for only task 3 and task 6.

Supplementary Table 2. Testing summary for other web based LLMs

LLMs	Release	Est Parameter size	Context window size	Test dates	Pipeline test (natural language	Pipeline test (structured prompt)	Tool replacement geNomad	Tool replacement VirSorter2

					prompt) (success/t otal)	(success/t otal)	(success/t otal)	(success/t otal)
Claude 3.5 Sonnet	06/20/2 024	175 billion	200,000 tokens	08/20 24	0/10	3/10	0/10<br>10/10	9/10<br>10/10
Claude 3.7 Sonnet	02/24/2 025	150-250 billion	200,000 tokens	05/20 25	8/10	9/10	10/10	5/10
Claude Sonnet 4	05/22/2 025	50-100 billion	200,000 tokens	05/20 25	0/10	9/10	10/10	10/10
Claude Opus 4	05/22/2 025	300-500 billion	200,000 tokens	05/20 25	2/10	10/10	10/10	8/10
Claude Sonnet 4.5	09/2025		200,000 tokens	09/20 25	9/10	10/10	9/10	10/10
DeepSee kR1	01/20/2 025	671 billion	128,000 tokens	05/20 25	3/10	5/10	5/10	8/10
Meta Llama 3	04/18/2 024	8 billion	8000 tokens	08/20 24	0/10	0/10	0/10	0/10
Google Gemini 1.5 Flash	05/24/2 024	8 billion	1,048,5 76 tokens	08/20 24	0/10	0/10	0/10	0/10
Google Gemini 2.5 Flash	06/17/2 025	n/a	1,048,5 76 tokens	09/20 25	4/10	7/10	0/10	4/10
Google Gemini 2.5 Pro	06/17/2 025	n/a	1,048,5 76 tokens	09/20 25	7/10	9/10	10/10	10/10

Note: Claude 3.5 Sonnet in tool replacement has two testing case, first row is without documentation, second row is with documentation.

Supplementary Table 3: All external tools used in this work

Existing tools	Release year	Version	Available Link
Trimmomatic	2014	0.39	https://github.com/usadellab/Trimmomatic
Metaviral SPAdes	2020	3.15	https://github.com/ablab/spades
QUAST	2013	5.0	https://github.com/ablab/quast
CheckV	2021	0.8	https://bitbucket.org/berkeleylab/checkv/src/master/
Kraken 2	2019	2.1.5	https://github.com/DerrickWood/kraken2
VirSorter2	2021	2.2.4	https://github.com/jiarong/VirSorter2
vRhyme	2022	1.1	https://github.com/AnantharamanLab/vRhyme
PHAMB	2022	1.0.1	https://github.com/RasmussenLab/phamb

geNomad	2024	1.11	https://github.com/apcamargo/genomad
iPHoP	2023	1.3.3	https://bitbucket.org/srouxjgi/iphop/src/main/

Supplementary Table 4: Chat sessions for ten testing cases to generate metaviral pipeline using natural language prompts in ChatGPT-3.5.

Test	Link
1	https://chatgpt.com/share/a9467dc7-6634-42ec-8cf0-8b0a7c49411a
2	https://chatgpt.com/share/94c7569a-831c-477d-b8ed-92561909de64
3	https://chatgpt.com/share/23f25c4b-343e-4bf9-8400-1d3d0707e074
4	https://chatgpt.com/share/538e6d0d-74a5-4116-abc3-d86fe5ac810e
5	https://chatgpt.com/share/02a40735-87e4-4efe-a2af-2d912fa984f8
6	https://chatgpt.com/share/ac57e3b9-a64e-43a2-931a-614019ffc424
7	https://chatgpt.com/share/28829811-b3ce-4181-b395-4c1771615cd8
8	https://chatgpt.com/share/279cee00-8d30-4ec5-b97a-1d810b0c2147
9	https://chatgpt.com/share/35ab99c0-cdfe-4ae8-a765-be4f2d6aa230
10	https://chatgpt.com/share/3eccf228-2fa9-433f-be10-54e05533484b

Supplementary Table 5: Chat sessions for ten testing cases to generate metaviral pipeline using natural language prompts in ChatGPT-4.

Test	Link
1	https://chatgpt.com/share/7367091e-456e-4333-85f4-126986978994
2	https://chatgpt.com/share/07eab24c-e32f-489a-a656-1e832662cb02
3	https://chatgpt.com/share/28785178-8e09-418b-ad38-c4e4fc335602
4	https://chatgpt.com/share/9bb411bb-2770-4037-adbc-eeccfe500683
5	https://chatgpt.com/share/e21e4f38-e7ad-4a5d-b5e8-8349fd81f1fd
6	https://chatgpt.com/share/43942f89-2bd5-4a1d-8101-03d2b233437a
7	https://chatgpt.com/share/4797d21b-e3a6-436f-a81f-d2cdf5d7200e
8	https://chatgpt.com/share/bdf3eb17-9111-4cc2-a10a-48fabdd81b6f
9	https://chatgpt.com/share/47ec6c9e-52de-495e-a88f-395d5778e22d
10	https://chatgpt.com/share/62b9806f-9aad-40b2-9f42-d43a5129e220

Supplementary Table 6: Chat sessions for ten testing cases to generate metaviral pipeline using natural language prompts in ChatGPT-4o.

Test	Link
1	https://chatgpt.com/share/c9b09c23-f759-493e-8276-e62a0d4cf742
2	https://chatgpt.com/share/cba84c58-823b-4edc-a1f5-ef71c6a3bd25
3	https://chatgpt.com/share/b78c2609-001d-4b0d-b328-44cb886da603
4	https://chatgpt.com/share/14235787-e925-47cd-978d-a00fd237f1c2
5	https://chatgpt.com/share/8980bebc-5f43-42e6-ac98-6888bcc706f3
6	https://chatgpt.com/share/0b12147d-936e-4b54-afb4-cfd25490c4cc

7	https://chatgpt.com/share/63a98b1e-73f1-4628-9a1b-cac0a42cb808
8	https://chatgpt.com/share/3b1bfe20-c7d7-4e70-b11a-ecb9f0cbf8a6
9	https://chatgpt.com/share/2f1bb753-25f1-4b14-8d4a-0194f8511cd4
10	https://chatgpt.com/share/9aa6724d-0d33-44e0-932b-f3b330b551fd

Supplementary Table 7: Chat sessions for ten testing cases to generate metaviral pipeline using natural language prompts in ChatGPT-o1-mini.

Test	Link
1	https://chatgpt.com/share/66e85823-0628-8013-a481-21b077158e36
2	https://chatgpt.com/share/66e85839-233c-8013-9736-ceaec2627587
3	https://chatgpt.com/share/66e85849-6c10-8013-9f0f-53424b0fa13c
4	https://chatgpt.com/share/66e8585e-7c94-8013-9cdc-36221d5ebb3d
5	https://chatgpt.com/share/66e8586e-5cdc-8013-99b0-92757a79606a
6	https://chatgpt.com/share/66e8587f-3edc-8013-bec2-f213b5073cd4
7	https://chatgpt.com/share/66e8588c-709c-8013-9d28-df662d5fa118
8	https://chatgpt.com/share/66e858b9-81bc-8013-b936-048b9f0b4106
9	https://chatgpt.com/share/66e858c6-3020-8013-beec-c2ab67860d63
10	https://chatgpt.com/share/66e858d2-e4ac-8013-a8b8-429f1444ecc8

Supplementary Table 8: Chat sessions for ten testing cases to generate metaviral pipeline using natural language prompts in ChatGPT-o1-preview.

Test	Link
1	https://chatgpt.com/share/66e8616f-6a2c-8013-be9e-b4a483a16178
2	https://chatgpt.com/share/66e8617b-aa40-8013-8ed5-4bf79b697fff
3	https://chatgpt.com/share/66e8618a-1e7c-8013-8866-0e8f07679d95
4	https://chatgpt.com/share/66e8619c-9988-8013-ab2b-f7464cc0cc42
5	https://chatgpt.com/share/66e861b6-9554-8013-b93e-8bab6466c6d9
6	https://chatgpt.com/share/66e861de-e6bc-8013-ae8e-c90f4454ac32
7	https://chatgpt.com/share/66e861ed-3cf4-8013-a379-00191b5ca104
8	https://chatgpt.com/share/66e861fd-6f68-8013-889d-b5f112cf0757
9	https://chatgpt.com/share/66e8620c-9490-8013-8b3c-0ba4ca02a10c
10	https://chatgpt.com/share/66e86233-63a8-8013-a65a-faafd468079b

Supplementary Table 9: Chat sessions for ten testing cases to generate metaviral pipeline using natural language prompts in ChatGPT-4.1.

Test	Link
1	https://chatgpt.com/share/682b60b7-b168-8013-8bf1-58d4da1904bb
2	https://chatgpt.com/share/682b60d8-2b7c-8013-b4dc-ddd68d285ba7
3	https://chatgpt.com/share/682b60eb-7a8c-8013-bb4d-fe90ad918092
4	https://chatgpt.com/share/682b613e-4024-8013-9c66-afb0818a72e6
5	https://chatgpt.com/share/682b6153-29a8-8013-bd57-32dc378de8bc
6	https://chatgpt.com/share/682b616b-5648-8013-aee5-97e42e89abf7
7	https://chatgpt.com/share/682b6185-4100-8013-946c-7fc8c28b5b8b

8	https://chatgpt.com/share/682b6195-b5d8-8013-94f7-cc27e8e1ce72
9	https://chatgpt.com/share/682b61aa-e338-8013-821d-9c50a0d4334e
10	https://chatgpt.com/share/682b6206-2d9c-8013-8792-fdf920911a75

Supplementary Table 10: Chat sessions for ten testing cases to generate metaviral pipeline using natural language prompts in ChatGPT-4.1 mini.

Test	Link
1	https://chatgpt.com/share/682b6c62-82e8-8013-be64-c8f44e3ed029
2	https://chatgpt.com/share/682b6cd4-f3d0-8013-9b13-3588e440f503
3	https://chatgpt.com/share/682b6da5-5030-8013-ae9b-3f40e39fa03b
4	https://chatgpt.com/share/682b6e8e-2138-8013-a62a-16cb685d364c
5	https://chatgpt.com/share/682b6f59-b070-8013-b8bb-a611b9fdff20
6	https://chatgpt.com/share/682b6f81-b1c4-8013-9212-d8efb57e2307
7	https://chatgpt.com/share/682b702f-b4d8-8013-91fa-5f65af024046
8	https://chatgpt.com/share/682b703d-342c-8013-820c-f2e007c5835e
9	https://chatgpt.com/share/682b7062-ac88-8013-9716-5e0310b245bc
10	https://chatgpt.com/share/682b708e-84b8-8013-9755-f1f3974aa4cb

Supplementary Table 11: Chat sessions for ten testing cases to generate metaviral pipeline using natural language prompts in ChatGPT-4.5.

Test	Link
1	https://chatgpt.com/canvas/shared/682f8948bc44819186d1e336c3f5ca60
2	https://chatgpt.com/canvas/shared/6830b35068648191b419d3ff1a3cd106
3	https://chatgpt.com/canvas/shared/6830b36fa9d88191a3ba7d1d0b64cbf5
4	https://chatgpt.com/canvas/shared/6830b468efa0819189114a9a5c52eb83
5	https://chatgpt.com/canvas/shared/6830b48e12148191ad7a86a4b45e89dc
6	https://chatgpt.com/canvas/shared/6830b4a65f248191a5f77aa5ccdaf365
7	https://chatgpt.com/canvas/shared/6830b4b88b1c8191aaef8b20315b0c2e
8	https://chatgpt.com/canvas/shared/6830b4c9037c8191bbb7ad8a1ecf9aa6
9	https://chatgpt.com/canvas/shared/6830b4da1ffc81918a4af03a95df7d72
10	https://chatgpt.com/canvas/shared/6830b4e9a3e08191b42a4eab57542e6d

Supplementary Table 12: Chat sessions for ten testing cases to generate metaviral pipeline using natural language prompts in ChatGPT-5.

Test	Link
1	https://chatgpt.com/share/68bdc735-a4f8-8013-be12-b98f4e1b8ead
2	https://chatgpt.com/share/68bdc74e-4a10-8013-8b6e-c9dfb1668e13
3	https://chatgpt.com/share/68bdc75d-2ea0-8013-baf2-7642699162ee
4	https://chatgpt.com/share/68bdc76c-fbe8-8013-93b6-991657cc7d53
5	https://chatgpt.com/share/68bdc77b-e4f8-8013-ad69-2babd6739720
6	https://chatgpt.com/share/68bdc793-a090-8013-a0b9-aaa221722257
7	https://chatgpt.com/share/68bdc7a4-7950-8013-ba0d-700f45013dae
8	https://chatgpt.com/share/68bdc8e7-aad8-8013-8e12-78435f570654

9	https://chatgpt.com/share/68bdc8f6-8654-8013-b35c-28b97c6fd772
10	https://chatgpt.com/share/68bdc908-e978-8013-88c0-538286706d8f

Supplementary Table 13: Chat sessions for ten testing cases to generate metaviral pipeline using natural language prompts in Google Gemini 2.5 Flash.

Test	Link
1	https://g.co/gemini/share/fa52f19f8799
2	https://g.co/gemini/share/4e27b896706e
3	https://g.co/gemini/share/c46ed01a66e0
4	https://g.co/gemini/share/e08fd89dd76c
5	https://g.co/gemini/share/be1f97c633f0
6	https://g.co/gemini/share/cbbc78142723
7	https://g.co/gemini/share/79ceac4e4026
8	https://g.co/gemini/share/0b2bfca6c026
9	https://g.co/gemini/share/c19b682e76c4
10	https://g.co/gemini/share/b6770c24a69b

Supplementary Table 14: Chat sessions for ten testing cases to generate metaviral pipeline using natural language prompts in Google Gemini 2.5 Pro.

Test	Link
1	https://g.co/gemini/share/af2fb1281ce7
2	https://g.co/gemini/share/1eaf59658a07
3	https://g.co/gemini/share/3f1b61dbfa08
4	https://g.co/gemini/share/25c0294d70b8
5	https://g.co/gemini/share/3b77ef3727ca
6	https://g.co/gemini/share/2d9196b66fb9
7	https://g.co/gemini/share/16b58f8e15d4
8	https://g.co/gemini/share/30d3520fd458
9	https://g.co/gemini/share/a56a90ffd7f5
10	https://g.co/gemini/share/89657d92cece

Supplementary Table 15: Chat sessions for ten testing cases to generate metaviral pipeline using structured prompts in ChatGPT-o1-mini.

Test	Link
1	https://chatgpt.com/share/66e866a8-9490-8013-a6c6-fd43cf304458
2	https://chatgpt.com/share/66e866bc-56fc-8013-9187-a3644ecee60c
3	https://chatgpt.com/share/66e87368-e0dc-8013-b1c0-ad70f60a6d9b
4	https://chatgpt.com/share/66e87375-6df4-8013-b552-c00acdfbe890
5	https://chatgpt.com/share/66e873d7-8aec-8013-8060-8a9ff3abdfe5
6	https://chatgpt.com/share/66e873e5-6f10-8013-aae3-c83d98a4942b
7	https://chatgpt.com/share/66e873f0-9b24-8013-8dee-8b588fa54794
8	https://chatgpt.com/share/66e873fb-bab0-8013-bebf-d86dcb77d207

9	https://chatgpt.com/share/66e87406-8ad4-8013-ba39-8ad58291e559
10	https://chatgpt.com/share/66e87411-68d4-8013-8ac6-ca2a834b9dad

Supplementary Table 16: Chat sessions for ten testing cases to generate metaviral pipeline using structured prompts in ChatGPT-o1-preview.

Test	Link
1	https://chatgpt.com/share/66e8797c-3234-8013-80e0-444c4162230b
2	https://chatgpt.com/share/66e87989-dbd4-8013-a27e-fde024d6b963
3	https://chatgpt.com/share/66e87998-b82c-8013-ad73-0720705bdb87
4	https://chatgpt.com/share/66e879a7-3fa8-8013-9ef6-219282eb5041
5	https://chatgpt.com/share/66e879b6-0874-8013-a0f8-8295cf45bce1
6	https://chatgpt.com/share/66e879c3-1ea8-8013-956d-62fb9ccfda12
7	https://chatgpt.com/share/66e879d2-ac8c-8013-a009-8200e1e39ed2
8	https://chatgpt.com/share/66e879e1-a598-8013-add0-a9e1b099d18b
9	https://chatgpt.com/share/66e879f1-20e8-8013-a6dd-5835494a6d60
10	https://chatgpt.com/share/66e87a01-7c04-8013-8c63-c6556580c4d2

Supplementary Table 17: Chat sessions for ten testing cases to generate metaviral pipeline using structured prompts in ChatGPT-4.1.

Test	Link
1	https://chatgpt.com/share/682b7d8a-3744-8013-b9f5-34a27b05fca9
2	https://chatgpt.com/share/682b7df3-80e0-8013-a2d7-6452f8371bc0
3	https://chatgpt.com/share/682b7ee4-0a18-8013-b903-4e3a3734a688
4	https://chatgpt.com/share/682b7f3b-dd18-8013-bff6-484d689b4f82
5	https://chatgpt.com/share/682b7fc5-2470-8013-bfd3-9250484ef2fa
6	https://chatgpt.com/share/682b809b-3370-8013-aac9-e0c527d4123d
7	https://chatgpt.com/share/682b8194-8700-8013-955a-0e7e50a12af9
8	https://chatgpt.com/share/682b8221-9744-8013-8201-518dac8b5338
9	https://chatgpt.com/share/682b8269-50f0-8013-a020-82701022bee9
10	https://chatgpt.com/share/682b82ad-770c-8013-b99c-50efea15fc71

Supplementary Table 18: Chat sessions for ten testing cases to generate metaviral pipeline using structured prompts in ChatGPT-4.1 mini.

Test	Link
1	https://chatgpt.com/share/682b8300-a288-8013-b1b1-a2ef9c245826
2	https://chatgpt.com/share/682b8418-cc80-8013-b603-c789666866f0
3	https://chatgpt.com/share/682b8460-6d3c-8013-987d-380e91d48c8a
4	https://chatgpt.com/share/682b849b-48ec-8013-973f-78f377b8d1c9
5	https://chatgpt.com/share/682b84c5-6e24-8013-873d-34c7d191149c
6	https://chatgpt.com/share/682b84f7-ed04-8013-b1c5-46deb22fef3a
7	https://chatgpt.com/share/682b851d-0414-8013-9cdb-9b4e0323d2fc
8	https://chatgpt.com/share/682b85e1-1e2c-8013-b599-d29d5d4760ae
9	https://chatgpt.com/share/682b865e-01f4-8013-b9a2-7d2d16350c98

10	https://chatgpt.com/share/682b8684-fef0-8013-be99-832242d2002e

Supplementary Table 19: Chat sessions for ten testing cases to generate metaviral pipeline using structured prompts in ChatGPT-4.5.

Test	Link
1	https://chatgpt.com/canvas/shared/6830a67d61b48191b38ce5737329d534
2	https://chatgpt.com/canvas/shared/6830a6c3393c8191a66117e128185fd5
3	https://chatgpt.com/canvas/shared/682f98d068008191ba041da11ca721cb
4	https://chatgpt.com/canvas/shared/682f9b1d8bf88191b4bc289ae048b87f
5	https://chatgpt.com/canvas/shared/682f9b3b5f0081919905cb0fcb391693
6	https://chatgpt.com/canvas/shared/682f9b527c308191a521b6e011f39add
7	https://chatgpt.com/canvas/shared/682f9b73cd6c819182b829ba16a80a74
8	https://chatgpt.com/canvas/shared/682f9b8d2d7881919a866fad2fcebb43
9	https://chatgpt.com/canvas/shared/682f9ba4628881918573f1c542b65b65
10	https://chatgpt.com/canvas/shared/682f9bb40b4881918ff03ac621a41177

Supplementary Table 20: Chat sessions for ten testing cases to generate metaviral pipeline using structured prompts in ChatGPT-5.

Test	Link
1	https://chatgpt.com/share/68bdb262-59d4-8013-bb1c-d3da52b4d9b1
2	https://chatgpt.com/share/68bdb27f-18e8-8013-bd9f-60e698b5a31c
3	https://chatgpt.com/share/68bdb381-3744-8013-8f79-7ef38520ad98
4	https://chatgpt.com/share/68bdb395-3208-8013-95b0-e7b0274e09fe
5	https://chatgpt.com/share/68bdb3ac-9808-8013-82cb-0e8a76d6f3e6
6	https://chatgpt.com/share/68bdb3c3-0ddc-8013-8734-c3bb23786013
7	https://chatgpt.com/share/68bdb3f8-e544-8013-94be-d8192bb0bf70
8	https://chatgpt.com/share/68bdb409-76c4-8013-9bcb-123242a2a9fb
9	https://chatgpt.com/share/68bdb416-f2e4-8013-a01f-8a095e481e0f
10	https://chatgpt.com/share/68bdb428-79f8-8013-9a0b-f291fb9efb1c
11	https://chatgpt.com/share/68c85130-df50-8013-aa6b-a1e4ecd046ab
12	https://chatgpt.com/share/68c852df-c710-8013-92ff-a59ea935fb06
13	https://chatgpt.com/share/68c853c3-ede0-8013-9da2-cc11c1ac2b2c
14	https://chatgpt.com/share/68c854da-e008-8013-a6d2-f20c4daa42fc
15	https://chatgpt.com/share/68c8553d-604c-8013-b09a-46a53535a077
16	https://chatgpt.com/share/68c85bd3-f07c-8013-b455-edb39a08a8bc
17	https://chatgpt.com/share/68c85c5e-e9dc-8013-aa3b-abb88a86683c
18	https://chatgpt.com/share/68c85cd4-aed8-8013-a940-f91a454bcbb8
19	https://chatgpt.com/share/68c85d78-b738-8013-a928-16a3ea7fe81d
20	https://chatgpt.com/share/68c85e29-a2ec-8013-975b-7cb05745eeb0
21	https://chatgpt.com/share/68c85eab-80d4-8013-a8ec-8c7be585a754
22	https://chatgpt.com/share/68c85fc2-92d4-8013-a7fd-8bab0c49ed43
23	https://chatgpt.com/share/68c8605b-a398-8013-a87f-72f4625c5925

24	https://chatgpt.com/share/68c860b6-d680-8013-9046-bde89b572780
25	https://chatgpt.com/share/68c86131-b954-8013-8865-ab25736cfe4f
26	https://chatgpt.com/share/68c8618a-4e18-8013-82ec-dd45aa00b7dc
27	https://chatgpt.com/share/68c861f9-fef8-8013-81c5-81268c7274de
28	https://chatgpt.com/share/68c862e7-7e68-8013-ba1f-ac58541c6501
29	https://chatgpt.com/share/68c863dc-dbac-8013-9c83-176cd5cf5bdc
30	https://chatgpt.com/share/68c8649a-dfe8-8013-a031-88ecbb47e13a
31	https://chatgpt.com/share/68c865a5-7b6c-8013-b50b-79fe54a16f88
32	https://chatgpt.com/share/68c86602-18a4-8013-a184-57d7877c9de2
33	https://chatgpt.com/share/68c8668f-d1c8-8013-998e-989c073ce0a0
34	https://chatgpt.com/share/68c86700-9238-8013-b546-e84a6c4fd891
35	https://chatgpt.com/share/68c867cb-dd90-8013-aae6-6b47e594a27b
36	https://chatgpt.com/share/68c8686e-dcdc-8013-b811-40240275bde2
37	https://chatgpt.com/share/68c868ec-f0bc-8013-8394-59def1e5f954
38	https://chatgpt.com/share/68c86ad2-165c-8013-a22d-4aa169cf1dbe
39	https://chatgpt.com/share/68c86b44-7464-8013-a841-a9fddfe954b5
40	https://chatgpt.com/share/68c86d42-ee44-8013-a715-c51e48592256
41	https://chatgpt.com/share/68c86e33-a890-8013-9abe-e5cc789a4d68
42	https://chatgpt.com/share/68c86ea5-f7a4-8013-9e95-819be0942367
43	https://chatgpt.com/share/68c86ff9-e344-8013-9603-466e14ed9133
44	https://chatgpt.com/share/68c87085-ddb0-8013-97fc-f7a83b7e55cc
45	https://chatgpt.com/share/68c87192-f9c0-8013-b04a-d9982ff6cbcf
46	https://chatgpt.com/share/68c8725d-7910-8013-b5a0-6b98f4ae632c
47	https://chatgpt.com/share/68c872c6-d780-8013-afba-5e5302a1d0ee
48	https://chatgpt.com/share/68c87347-4214-8013-b00a-8173cfb61e40
49	https://chatgpt.com/share/68c873ce-cafc-8013-831f-16587826abdc
50	https://chatgpt.com/share/68c8745c-128c-8013-be21-045bf52483e8

Supplementary Table 21: Chat sessions for ten testing cases to generate metaviral pipeline using structured prompts in Google Gemini 2.5 Flash.

Test	Link
1	https://g.co/gemini/share/39029a48cf40
2	https://g.co/gemini/share/d3b47250855e
3	https://g.co/gemini/share/fb05e5f96710
4	https://g.co/gemini/share/a0c127d9c932
5	https://g.co/gemini/share/73292ef85f9b
6	https://g.co/gemini/share/6efe41a85350
7	https://g.co/gemini/share/afbee2772342
8	https://g.co/gemini/share/4a534692bbd5
9	https://g.co/gemini/share/0afcc37ffd1f
10	https://g.co/gemini/share/7f7932d36176

Supplementary Table 22: Chat sessions for ten testing cases to generate metaviral pipeline using structured prompts in Google Gemini 2.5 Pro.

Test	Link
1	https://g.co/gemini/share/4d71c4223228
2	https://g.co/gemini/share/aa838af1d396
3	https://g.co/gemini/share/01e83b241fa2
4	https://g.co/gemini/share/52d968dbc40c
5	https://g.co/gemini/share/c0bdcd4534c3
6	https://g.co/gemini/share/16e8cb197ef6
7	https://g.co/gemini/share/554106f59f39
8	https://g.co/gemini/share/06e88e1edd5b
9	https://g.co/gemini/share/cfd40bd6ac62
10	https://g.co/gemini/share/25ffdd22e4ee

Supplementary Table 23: Chat sessions for fifty testing cases to generate metaviral pipeline using structured prompts in ChatGPT-4 (Dec 2023)

Test	Link
1	https://chat.openai.com/share/fcf93f36-a224-4227-818d-97fde23cc217
2	https://chat.openai.com/share/6933e09a-f504-4d28-b3df-a7ea4bc4eb7c
3	https://chat.openai.com/share/5b4d5980-00c5-43cd-b1ac-0a40601ae49b
4	https://chat.openai.com/share/319d6f24-a04a-44ac-9a2d-f4918144e140
5	https://chat.openai.com/share/98d6d0aa-dc8b-4c1e-bbda-964cf8b90789
6	https://chat.openai.com/share/8684361c-9c3a-4fa1-9b81-3c435f9c4fd2
7	https://chat.openai.com/share/454d8244-e95a-416a-b4fe-b85ea193809e
8	https://chat.openai.com/share/90c8e43e-05f7-426f-b113-1df5d0f9c704
9	https://chat.openai.com/share/cc4d1344-f26d-4167-bd4a-d118971d1122
10	https://chat.openai.com/share/0166df6b-23ea-480b-9fec-f5dc999a0a49
11	https://chat.openai.com/share/64ffdf52-a2f7-4ce3-8ade-131cf0d5b901
12	https://chat.openai.com/share/5089d329-1a3c-44e1-91a2-20905fa4707d
13	https://chat.openai.com/share/0a91b16f-91e5-4d65-a1cc-562027d440ce
14	https://chat.openai.com/share/e824529b-9b0c-430a-8a2f-b1251cea9493
15	https://chat.openai.com/share/fc4d34b3-479a-4a78-97fd-6a90629def6a
16	https://chat.openai.com/share/3a25172a-7255-446d-ae4e-a32c43ff72ae
17	https://chat.openai.com/share/971501e6-f81d-45f6-b11d-149bc5c6fad6
18	https://chat.openai.com/share/edc58f0d-d4cd-43e8-854a-66768e1c34ea
19	https://chat.openai.com/share/d968f34f-f524-482c-9134-6e1b5b1d3037
20	https://chat.openai.com/share/afc113b9-28cc-42a0-b7c6-5991f97479a0
21	https://chat.openai.com/share/e1dd6409-3cae-4bd8-bcb4-6a1f444104e1
22	https://chat.openai.com/share/f7557e20-e1ee-42ad-8e97-3d7ba7b02eea
23	https://chat.openai.com/share/404c3a8f-3954-42d1-a872-f7c190be5903
24	https://chat.openai.com/share/90fbb231-8887-4837-8a58-f4a78aa1cba2

25	https://chat.openai.com/share/a9f1be52-fbc8-4b38-afb5-9f53d4cf19a8
26	https://chat.openai.com/share/3da4197f-f9d0-4197-9da9-d45a7fd6feb1
27	https://chat.openai.com/share/aebba0b0-46c7-4dc6-bacb-7005a89c30c2
28	https://chat.openai.com/share/ad3f5e64-941e-48eb-b13d-93f2d2038310
29	https://chat.openai.com/share/f38a1ef2-c3d8-42f9-aee2-418ec512f878
30	https://chat.openai.com/share/9c78e79e-9865-41f9-8d9d-e2986cb56807
31	https://chat.openai.com/share/a50ceff8-2b3f-41f4-8adc-e5047825611d
32	https://chat.openai.com/share/d897f5b6-c22f-42f5-bab2-384f050060cf
33	https://chat.openai.com/share/346efddf-2a7e-4f22-a71d-4d303b57c88d
34	https://chat.openai.com/share/c0c99f5a-63ee-4151-bc74-f1d9bd2af5d3
35	https://chat.openai.com/share/f80e1a18-6e76-4ec9-8ac6-095c57d9206e
36	https://chat.openai.com/share/9829ec82-99aa-476b-8977-39e6c7b4a6c1
37	https://chat.openai.com/share/c9c7e653-e837-4d98-9e08-96969b414c99
38	https://chat.openai.com/share/16474a88-e5ec-4da6-94ea-6d201cc3eb12
39	https://chat.openai.com/share/4a1a66f1-e507-487b-b2c0-82952b35a2bd
40	https://chat.openai.com/share/b2653702-8bd5-420c-b50a-ce2fadde47c3
41	https://chat.openai.com/share/b7ee9fed-6784-4e96-aab8-2ae63f81c1a6
42	https://chat.openai.com/share/08bb3ed4-6a3f-4e01-8083-f35db6b82bd1
43	https://chat.openai.com/share/16cc363a-dc6c-48e8-a7f7-9cae25c6ea43
44	https://chat.openai.com/share/8df228b0-1dfa-40f3-8f5f-bd823c56735f
45	https://chat.openai.com/share/947df93f-2ff6-49ad-80a1-8e909fac1ac1
46	https://chat.openai.com/share/1cb7f5c2-b42d-4d27-8370-8f900a44b7bd
47	https://chat.openai.com/share/6b2134db-03f5-44ba-8f72-fccf436e3759
48	https://chat.openai.com/share/b28212a7-f9eb-42be-bb42-5d05a116d057
49	https://chat.openai.com/share/c6f42e3e-c54e-432e-bd8f-54a080ce772c
50	https://chat.openai.com/share/68c2899f-a79f-4bbf-8480-39a7b5aac989

Supplementary Table 24: Correctness of metaviral pipeline codes execution from fifty testing cases (reported in Supplementary Table 23) using structured prompts in ChatGPT-4 (Dec 2023)

Test	Task 1	Task 2	Task 3	Task 4	Task 5	Task 6
1	√	×	√	√	√	√
2	√	√	√	√	√	√
3	√	√	√	√	√	√
4	√	√	√	√	√	√
5	√	√	√	√	√	√
6	√	×	√	√	√	√
7	√	√	√	√	√	√
8	√	√	√	√	√	√
9	√	×	√	√	√	√
10	√	√	√	√	√	√
11	√	√	√	√	√	√

12	√	√	√	√	√	√
13	√	×	√	√	√	√
14	√	×	√	√	√	√
15	√	×	√	√	√	√
16	√	√	√	√	√	√
17	√	√	√	√	√	√
18	√	√	√	√	√	√
19	√	√	√	√	√	√
20	√	√	√	√	√	√
21	√	√	√	√	√	√
22	√	√	√	√	√	√
23	√	√	√	√	√	√
24	√	√	√	√	√	√
25	√	×	√	√	√	√
26	√	√	√	√	√	√
27	√	√	√	√	√	√
28	√	×	√	√	√	√
29	√	√	√	√	√	√
30	√	√	√	√	√	√
31	√	×	√	√	√	√
32	√	√	√	√	√	√
33	√	×	√	√	√	√
34	√	√	√	√	√	√
35	√	×	√	√	√	√
36	√	√	√	√	√	√
37	√	×	√	√	√	√
38	√	√	√	√	√	√
39	√	√	√	√	√	√
40	√	×	√	√	√	√
41	√	√	√	√	√	√
42	√	×	√	√	√	√
43	√	√	√	√	√	√
44	√	×	√	√	√	√
45	√	×	√	√	√	√
46	√	√	√	√	√	√
47	√	√	√	√	√	√
48	√	×	√	√	√	√
49	√	√	√	√	√	√
50	√	√	√	√	√	√
√: Pass; ×: Failed.						

```
# Task 2: Trimming reads
trimmomatic PE -threads 2 \
    hiseq_reads_R1.fastq hiseq_reads_R2.fastq \
    read1trimmed.fastq read1untrimmed.fastq \
    read2trimmed.fastq read2untrimmed.fastq adapters.fa
```

```
# Trimming the reads
trimmomatic PE \
    hiseq_reads_R1.fastq hiseq_reads_R2.fastq \
    read1trimmed.fastq read2trimmed.fastq \
    read1untrimmed.fastq read2untrimmed.fastq \
    ILLUMINACLIP:adapters.fa:2:30:10 \
    LEADING:3 TRAILING:3 SLIDINGWINDOW:4:15 MINLEN:36
```

Supplementary Table 25: Chat sessions for fifty testing cases to generate metaviral pipeline using structured prompts in ChatGPT-4 (version with DALL-E, external test)

Test	Link
1	https://chat.openai.com/share/181b4fb4-2472-4489-95fe-48acc400dd10
2	https://chat.openai.com/share/dc3e5a5e-426f-4bfa-a6b2-63b20aa22306
3	https://chat.openai.com/share/271a4ca4-2ffb-447c-9d3a-b3e8aa470812
4	https://chat.openai.com/share/8fa08927-2b56-44e1-9af0-e7a188554a5b
5	https://chat.openai.com/share/fed4bd58-9518-4b39-80d2-c214018474b9
6	https://chat.openai.com/share/7177c564-4831-44df-9d7f-660fb9cfc542
7	https://chat.openai.com/share/db64911a-1af2-4c8f-99a7-68123890d765
8	https://chat.openai.com/share/c90312d9-7841-4bf0-8267-0343822ebaf5
9	https://chat.openai.com/share/814ed624-38c0-415c-8a94-42055ba2cf6d
10	https://chat.openai.com/share/7d09d502-51a7-48cc-bb66-4ca52fd96d5b
11	https://chat.openai.com/share/b9c20c09-5a45-4ef3-8d36-6c8487070e5e
12	https://chat.openai.com/share/08de795e-c08c-40d4-8669-85550a24e8d7
13	https://chat.openai.com/share/00e4f1fb-3e74-4412-837b-fbf6004faf9c
14	https://chat.openai.com/share/75dfc38e-8fe7-42d5-a134-c504bbc95301
15	https://chat.openai.com/share/31b0c05c-643d-46ea-903d-e319e2401821
16	https://chat.openai.com/share/b80223d7-d910-4593-8af3-a62f6c17ea71
17	https://chat.openai.com/share/bd03e8c8-6ea1-406b-9c2c-a08883c8a45d
18	https://chat.openai.com/share/a131edea-c8a8-40a4-b45d-32d79eb2548e
19	https://chat.openai.com/share/9ade90a1-6d49-4ac6-965d-afde96a9902a
20	https://chat.openai.com/share/c38cb29d-16d5-47d3-a8d5-5bdcf4dd5a83
21	https://chat.openai.com/share/3c8ab3c9-26ad-4a1e-9dc2-4f09ffada266
22	https://chat.openai.com/share/055c1bbe-e255-4c76-9b9e-2ff17c9a5bb0
23	https://chat.openai.com/share/d089e7aa-308e-46bd-bf44-4daa6fe69f20
24	https://chat.openai.com/share/dbc0872a-1124-4a7a-803f-9e13b686c033
25	https://chat.openai.com/share/89f7f12a-98c7-47ed-9b2b-88d21d6d04e0
26	https://chat.openai.com/share/e62fbdf8-9295-4c65-93bb-64849f551baf
27	https://chat.openai.com/share/2b64aa0c-1ee6-41c3-b7bb-63a48f5a5264
28	https://chat.openai.com/share/e9e115db-54e0-4c5b-b350-d4f7b5ab14cb
29	https://chat.openai.com/share/ed69f71a-9437-4d2f-827e-f07cdb70a9d2
30	https://chat.openai.com/share/e1a72436-7c55-49c5-aa40-648d97bccbd9
31	https://chat.openai.com/share/fda01038-ea6d-465a-be56-fa6e62ed241f
32	https://chat.openai.com/share/42891b90-20b7-408d-899f-a629da60a185
33	https://chat.openai.com/share/0ccb8dda-9794-4056-b7db-9477fe0566a9

34	https://chat.openai.com/share/635570ee-71c9-4c50-bafb-b0ce2d38196f
35	https://chat.openai.com/share/8204e6f4-eab9-4a01-a631-feb82d976ef0
36	https://chat.openai.com/share/0e3c737e-9d7d-413c-9be8-515876988cfd
37	https://chat.openai.com/share/de967444-23b7-45a3-8914-aa9eaaae06c8
38	https://chat.openai.com/share/317c3b36-920a-47a5-beae-f8cf3b59d15a
39	https://chat.openai.com/share/97d49fff-a254-4bf3-be1f-3ccdb59a5001
40	https://chat.openai.com/share/dac21b73-b354-4840-941e-e7ab528ab1d9
41	https://chat.openai.com/share/4c563665-710e-4d6d-b26a-228de9e295cf
42	https://chat.openai.com/share/78f260ff-59a7-4543-8fe5-15659a68e50b
43	https://chat.openai.com/share/3d90b347-7d82-4ff2-a91b-85925a1c44f3
44	https://chat.openai.com/share/e98e0211-b358-432f-8c10-2118b459b747
45	https://chat.openai.com/share/e8f92817-8a1d-4744-9761-63f79961dd1a
46	https://chat.openai.com/share/9ab1be7e-2bd8-449e-8832-2cdf6085b748
47	https://chat.openai.com/share/9c621ee3-d06e-4864-89ef-95015624fe05
48	https://chat.openai.com/share/63ab26e7-e499-4325-b45f-950f17fc57b7
49	https://chat.openai.com/share/aa067280-f54b-400e-8149-3d613ff0587f
50	https://chat.openai.com/share/70bf3ecf-0247-4bc4-91a8-46d3ddfa26a0

Supplementary Table 26: Correctness of metaviral pipeline codes execution from fifty testing cases (reported in Supplementary Table 25) using structured prompts in ChatGPT-4 (version with DALL-E, external test)

Test	Task 1	Task 2	Task 3	Task 4	Task 5	Task 6
1	√	√	√	√	√	√
2	√	√	√	√	√	√
3	√	×	√	√	√	√
4	√	√	√	√	√	√
5	√	×	√	√	√	√
6	√	√	√	√	√	√
7	√	×	√	√	√	√
8	√	×	√	√	√	×
9	√	√	√	√	√	√
10	√	×	√	√	√	√
11	√	√	√	√	√	√
12	√	√	√	√	√	√
13	√	×	√	√	√	√
14	√	√	√	√	√	√
15	√	√	√	√	√	√
16	√	×	√	√	√	√
17	√	√	√	√	√	√
18	√	√	√	√	√	√
19	√	√	√	√	√	√
20	√	×	√	√	√	√

21	√	×	√	√	√	√
22	√	×	√	√	√	√
23	√	√	√	√	√	√
24	√	√	√	√	√	√
25	√	√	√	√	√	√
26	√	×	√	√	√	×
27	√	√	√	√	√	√
28	√	×	√	√	√	√
29	√	√	√	√	√	√
30	√	√	√	√	√	√
31	√	√	√	√	√	√
32	√	√	√	√	√	√
33	√	√	√	√	√	√
34	√	√	√	√	√	√
35	√	√	√	√	√	√
36	√	√	√	√	√	√
37	√	×	√	√	√	×
38	√	×	√	√	√	√
39	√	×	√	√	√	√
40	√	×	√	√	√	√
41	√	√	√	√	√	√
42	√	√	√	√	√	√
43	√	√	√	√	√	√
44	√	√	√	√	√	√
45	√	√	√	√	√	√
46	√	√	√	√	√	√
47	√	×	√	√	√	√
48	√	√	√	√	√	√
49	√	√	√	√	√	√
50	√	√	√	√	√	√

√: Pass; ×: Failed.

Task 2 common errors (fastq files are in the wrong order):

```
# Task 2: Trim reads
trimmomatic PE -threads $THREADS hiseq_reads_R1.fastq hiseq_reads_R2.fastq \
                read1trimmed.fastq read1untrimmed.fastq \
                read2trimmed.fastq read2untrimmed.fastq adapters.fa
```

```
# Task 2: Trim the files using Trimmomatic
trimmomatic PE \
  hiseq_reads_R1.fastq hiseq_reads_R2.fastq \
  read1trimmed.fastq read2trimmed.fastq \
  read1untrimmed.fastq read2untrimmed.fastq \
  ILLUMINACLIP:adapters.fa:2:30:10
```

Task 6 common errors (missing file name)

```
kraken2 --db custom_db read1trimmed.fastq --threads 2 --report reads_report.txt
```

Supplementary Table 27: Chat sessions for fifty testing cases to generate metaviral pipeline using structured prompts in ChatGPT-4o (June 2024)

Test	Link
1	https://chatgpt.com/share/472cf410-2b9e-4d99-b9ae-81d4267d9945
2	https://chatgpt.com/share/a919f76b-cb6a-460c-8f6e-a171bf796c95

3	https://chatgpt.com/share/5351f023-80ae-4b24-a7d2-85471e7989cb
4	https://chatgpt.com/share/76fd8466-ebaf-4f63-8f53-07ee5d3116f8
5	https://chatgpt.com/share/fae41be9-3ba7-4841-abf6-7a722a26d886
6	https://chatgpt.com/share/af26879d-cd68-455a-ac93-ae82af8b02ef
7	https://chatgpt.com/share/d989c839-e3e1-4f6d-94ac-25f8c3c91d3d
8	https://chatgpt.com/share/7867dbeb-581c-4cf9-b65b-28eb32eb4c45
9	https://chatgpt.com/share/0e3d7472-439d-4276-831f-146bcd90b9eb
10	https://chatgpt.com/share/037e9aaf-bb38-4fdc-bd72-8ab1f79e86a5
11	https://chatgpt.com/share/b5a5097a-3d36-4ab9-a402-b78c463597d2
12	https://chatgpt.com/share/eb9a14e3-d66f-4944-9be4-173a1940e611
13	https://chatgpt.com/share/ab09e488-17fc-4200-81fd-e7b549a4107b
14	https://chatgpt.com/share/5a2a8f9f-b040-415e-9abf-35894371f2cf
15	https://chatgpt.com/share/cff6848d-fe8d-48ea-baae-128d911af0c5
16	https://chatgpt.com/share/8a66d014-980c-403a-a897-b475d4ef3cb6
17	https://chatgpt.com/share/85bdda5f-0341-4f11-a1dc-9db1e4305fd3
18	https://chatgpt.com/share/b17c67be-27ca-4f89-b655-0372beade0cb
19	https://chatgpt.com/share/ab1f5abb-32b4-49ce-b23f-773a2b8c476d
20	https://chatgpt.com/share/c9c0dd62-5694-4eef-8e7f-273e94071cdf
21	https://chatgpt.com/share/c6992531-76ab-4ac9-a518-41d39f2b0a94
22	https://chatgpt.com/share/ec449b56-5dac-4747-82c0-232810e35cc7
23	https://chatgpt.com/share/044ec777-464d-46b1-a4e1-beb1471caa1b
24	https://chatgpt.com/share/954b0f0f-8f1e-4b4b-a59b-24ad753b9f9e
25	https://chatgpt.com/share/d858b4a2-c66e-45f1-aaae-c593386fd5de
26	https://chatgpt.com/share/9e63a117-753c-4095-a9fa-17a501086eb5
27	https://chatgpt.com/share/24827ccb-5cb9-4d6e-bc57-8a610c4d7a99
28	https://chatgpt.com/share/1c78b3e2-543c-48ca-9203-a97c6555a027
29	https://chatgpt.com/share/da40eddb-b9dc-4a39-9031-8efd5bb4322c
30	https://chatgpt.com/share/9b407e96-b630-4918-8903-e261ba75c3b8
31	https://chatgpt.com/share/46f48c0a-8c13-4f8a-9bad-f7cec1db0c6b
32	https://chatgpt.com/share/971eab1c-a629-48c1-bfa2-9b49b730f2d8
33	https://chatgpt.com/share/f33d5656-5dbb-4fac-a490-4b742ea33105
34	https://chatgpt.com/share/341857c4-ae28-4922-b36a-28f04ff19f03
35	https://chatgpt.com/share/f7c21a6f-61e0-4c99-b2ba-819f23e17e45
36	https://chatgpt.com/share/03e79338-7148-48f1-abe9-b10b2aee4398
37	https://chatgpt.com/share/01503fba-4dba-44f0-b368-2f3e97003901
38	https://chatgpt.com/share/0dde1e82-c851-4796-941a-85124f517ca6
39	https://chatgpt.com/share/8ad83a2b-4e07-4971-a31c-76b700cb77ea
40	https://chatgpt.com/share/b58122fb-6ad9-4347-8f5e-ad31fa7ee89c
41	https://chatgpt.com/share/3bdd4a83-2356-4946-acbb-227e2405ac53
42	https://chatgpt.com/share/1779c52b-c727-4e82-97ac-9b46c4861697
43	https://chatgpt.com/share/5138242b-fa36-4fef-b6c9-af6fb1bcc467

44	https://chatgpt.com/share/4393afdc-5f2b-4c65-9ce8-1caef38dc5ed
45	https://chatgpt.com/share/d0691986-1c28-4844-a9d8-8dd2c56cd484
46	https://chatgpt.com/share/ecd1d377-83af-4841-986d-cf2738fd416c
47	https://chatgpt.com/share/8c71f96d-6a66-4074-9dcf-d1e135c56f2d
48	https://chatgpt.com/share/22f3a6bc-e0df-4875-8309-134c5a6ba9be
49	https://chatgpt.com/share/ccb522cb-73d3-4059-824d-c3eddc8aee63
50	https://chatgpt.com/share/54ed9a3b-0dbd-452c-8955-040fbfdae55c

Supplementary Table 28: Correctness of metaviral pipeline codes execution from fifty testing cases (reported in Supplementary Table 27) using structured prompts in ChatGPT-4o (June 2024)

Test	Task 1	Task 2	Task 3	Task 4	Task 5	Task 6
1	×	√	√	√	√	√
2	√	√	√	√	√	√
3	√	√	√	√	√	√
4	√	√	√	√	√	√
5	×	√	√	√	√	√
6	√	√	√	√	√	√
7	√	√	√	√	√	√
8	√	√	√	√	√	√
9	√	√	√	√	√	√
10	√	√	√	√	√	√
11	√	√	√	√	√	√
12	×	√	√	√	√	√
13	√	√	√	√	√	×
14	√	√	√	√	√	√
15	√	√	√	√	√	√
16	√	√	√	√	√	√
17	√	√	√	√	√	√
18	√	×	√	√	√	√
19	√	√	√	√	√	√
20	√	√	√	√	√	√
21	√	√	√	√	√	√
22	√	√	√	√	√	√
23	√	√	√	√	√	√
24	√	√	√	√	√	√
25	√	√	√	√	√	√
26	√	√	√	√	√	√
27	√	√	√	√	√	√
28	√	√	√	√	√	√
29	×	√	√	√	√	√

30	√	√	√	√	√	√
31	×	√	√	√	√	√
32	√	√	√	√	√	√
33	√	√	√	√	√	√
34	√	√	√	√	√	√
35	√	√	√	√	√	√
36	√	√	√	√	√	√
37	×	√	√	√	√	×
38	√	√	√	√	√	√
39	√	√	√	√	√	√
40	√	√	√	√	√	√
41	√	√	√	√	√	√
42	√	×	√	√	√	√
43	√	√	√	√	√	√
44	×	√	√	√	√	√
45	√	×	√	√	√	√
46	√	√	√	√	√	√
47	√	√	√	√	√	√
48	√	×	√	√	√	√
49	√	√	√	√	√	√
50	√	×	√	√	√	√

√: Pass; ×: Failed.

Task 1 common errors (“module load” tools version is wrong):

```
module load trimmomatic/0.39 spades/3.15 quast/5.0 checkv/0.8 kraken2/2.0
```

Tash 2 common errors (fastq files are in the wrong order and missing paramater):

```
# Task 2: Trim the files
trimmomatic PE -threads 2 \
    hiseq_reads_R1.fastq hiseq_reads_R2.fastq adapters.fa \
    read1trimmed.fastq read1untrimmed.fastq \
    read2trimmed.fastq read2untrimmed.fastq
```

Task 6 common errors:

```
kraken2 --db custom_db read1trimmed.fastq --threads 2 --report reads_report.txt
```

Supplementary Table 29: Correctness of metaviral pipeline codes execution from fifty testing cases (reported in Supplementary Table 20) using structured prompts in ChatGPT-5

Test	Task 1	Task 2	Task 3	Task 4	Task 5	Task 6
1	√	√	√	√	√	√
2	√	√	√	√	×	√
3	√	√	√	√	√	√
4	√	√	√	√	√	√
5	√	√	√	√	√	√
6	√	√	√	√	√	√
7	√	√	√	√	√	√

8	√	√	√	√	√	√
9	√	√	√	√	√	√
10	√	√	√	√	√	√
11	√	√	√	√	√	√
12	√	√	√	√	√	√
13	√	√	√	√	√	√
14	√	√	√	√	√	√
15	√	√	√	√	√	√
16	√	√	√	√	√	√
17	√	√	√	√	√	√
18	√	√	√	√	√	√
19	√	√	√	√	√	√
20	√	√	√	√	√	√
21	√	√	√	√	√	√
22	√	√	√	√	√	√
23	√	√	√	√	√	√
24	√	√	√	√	√	√
25	√	√	√	√	√	√
26	√	√	√	√	√	√
27	√	√	√	√	√	√
28	√	√	√	√	√	√
29	√	√	√	√	√	√
30	√	√	√	√	√	√
31	√	√	√	√	√	√
32	√	√	√	√	√	√
33	√	√	√	√	√	√
34	√	√	√	√	√	√
35	√	√	√	√	√	√
36	√	√	√	√	√	√
37	√	√	√	√	√	√
38	√	√	√	√	√	√
39	√	√	√	√	√	√
40	√	√	√	√	√	√
41	√	√	√	√	√	√
42	√	√	√	√	√	√
43	√	√	√	√	√	√
44	√	√	√	√	√	√
45	√	√	√	√	√	√
46	√	√	√	√	√	√
47	√	√	√	√	√	√

48	√	√	√	√	√	√
49	√	√	√	√	√	√
50	√	√	√	√	√	√

Supplementary Table 30: Prompt engineering results in metaviral pipeline task 3.
Performance improvement by one-shot, stimulus, and complete (one-shot plus stimulus) prompting in Task 3 using different ChatGPT Model API.

**Task 3**	gpt-3.5-turbo-1106	gpt-4-0314	gpt-4-0613	gpt-4-1106-preview
**Complete**	50/0	50/0	50/0	50/0
**one-shot**	50/0	48/2	50/0	50/0
**Stimulus**	9/41	50/0	50/0	50/0
**base**	0/50	0/50	0/50	0/50
Plot	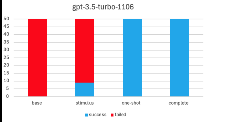	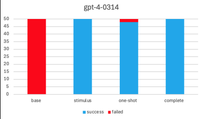	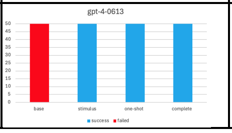	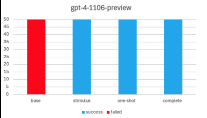
Numbers presented in “A/B”, where A = success, B = failed.<br>**Prompts send to GPT models through API (n=50 for each) *:**				
Act as an experienced bioinformatician proficient in viral genomic assembly and annotation tasks. Only when specifically requested, you will provide comments on the code. Reply “code completed” after each time of code generation. All required software is installed globally. Save the results from all tasks to the current working folder. Show me only the code. Absolutely no need to execute.<br>**Task 3**: Use SPAdes (St. Petersburg genome assembler) program using the 'spades.py' script with the "--metaviral" option for assembling viral metagenomic sequences.<br>Example: spades.py --metaviral --pe1-1 read1trimmed.fastq --pe1-2 read2trimmed.fastq -t 2 -o ./spadeout<br>Input: read1trimmed.fastq, read2trimmed.fastq<br>Output: spadeout folder<br>Tools: spades/3.15.<br>Coding language: bash				
* One-shot prompting marked in red; Stimulus prompting marked in purple; base prompting marked in black.				

Supplementary Table 31: Prompt engineering results in metaviral pipeline task 6.
Performance improvement by stimulus prompting in Task 6 using different ChatGPT Model API.

**Task 6**	gpt-3.5-turbo-1106	gpt-4-0314	gpt-4-0613	gpt-4-1106-preview
**Stimulus**	27/23	19/31	12/38	37/13
**base**	5/45	9/41	9/41	42/8
Plot	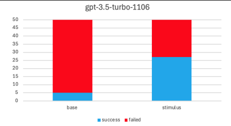	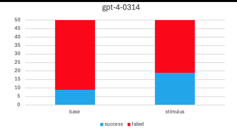	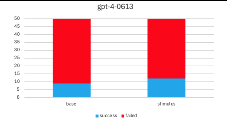	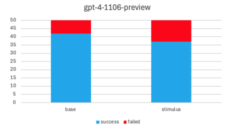
Numbers presented in “A/B”, where A = success, B = failed.<br>**Prompts send to GPT models through API (n=50 for each) *:**				
Act as an experienced bioinformatician proficient in viral genomic assembly and annotation tasks. Only when specifically requested, you will provide comments on the code. Reply “code completed” after each time of code generation. All required software is installed globally. Save the results from all tasks to the current working folder. Show me only the code. Absolutely no need to execute.<br>**Task 6:** Create a custom Kraken2 database, download taxonomy information, and add reference sequences from the Ref_database folder (need a for loop), then build the database without using any threads. Finally, use 2 threads annotate both contigs and filtered reads then use option "--report " generate reports for both.				

Input: Ref_database folder with. fa files, contigs.fasta, read1trimmed.fastq, read2trimmed.fastq<br>Output: two folders<br>Tools: kraken2<br>Coding language: bash<br>Common errors:<br>Missing input files after “--paired” option from Kraken.
* Stimulus prompting marked in purple; base prompting marked in black.<br>Task 6 is giving more errors, we add more details in prompt like use “--report” option in the tool which make this Task 6 can have 100% accuracy when generating the code.

Supplementary Table 32: Recent computational virome tools being tested.

Tool	Reference command line
2021-1, VirSorter2<br>Virus detection<br>https://github.com/jiarong/VirSorter2	virsorter run -w test.out -i contigs.fasta -j 4 all<br><br>(-j is CPU cores, which is not very important; all means entire pipeline).
2021-2, CheckV<br>Virus detection<br>https://bitbucket.org/berkeleylab/checkv/src/master	checkv end_to_end contigs.fasta checkv_output -t 16<br><br>(-t is CPU cores, which is not very important)
2022-1, vRhyme<br>Virus Binning<br>https://github.com/AnantharamanLab/vRhyme	vRhyme -i contigs.fasta -c coverage.tsv -o vrhyme_output -t 1<br><br>(-t 1 is not very important)
2022-2, PHAMB<br>Virus Binning<br>https://github.com/RasmussenLab/phamb	run_RF.py contigs.fna.gz clusters.tsv annotations phamb_output
2023-1, geNomad<br>Virus detection and annotation<br>https://portal.nersc.gov/genomad/	genomad end-to-end --cleanup --splits 8 contigs.fasta genomad_output genomad_db<br><br>(--cleanup --splits may not be important; contig.fasta is our input; genomad_db is pre-downloaded)
2023-2, iPHoP<br>Host prediction<br>https://bitbucket.org/srouxjgi/iphop/src/main/	iphop predict --fa_file genome.fasta --db_dir iphop_db/Test_db_rw/ --out_dir iphop_output<br><br>(database is pre-downloaded).

Supplementary Table 33: Chat sessions for testing a recent computational virome tool (VirSorter2).

VirSorter2	ChatGPT-3.5	Status	ChatGPT-4 Without document	Status	ChatGPT-4o Without document	Status	ChatGPT-4 With document	Status	ChatGPT-4o With document	Status	ChatGPT-5	Status

1	https://chatgpt.com/share/3d466c74-2966-4fd4-85a1-970746231450	×	https://chatgpt.com/share/d0d0e288-b861-458e-aa5f-1641c48eb338	√	https://chatgpt.com/share/c9892120-e097-4cf4-8a71-ae64d8c237cf	√	https://chatgpt.com/share/73be7bd5-edd1-455e-bce7-2b05ffe36215	√	https://chatgpt.com/share/1660c601-c767-417c-afe8-b0e313ae1815	√	https://chatgpt.com/share/68d6c0c4-5b90-8013-bf36-5d543fe87615	√
2	https://chatgpt.com/share/e4015408-b28f-4a43-a6e3-1b3cd409b66c	×	https://chatgpt.com/share/a68db61e-8271-4316-b9e7-dea61de7ba2a	√	https://chatgpt.com/share/0e06881c-0ccf-449c-974a-48c2bda5a0e8	√	https://chatgpt.com/share/ce5cd795-9936-46f4-b43c-eb16136e30ef	√	https://chatgpt.com/share/a7b20a23-19ea-4818-9857-64c72f99ce52	√	https://chatgpt.com/share/68d6cfaa-4f60-8013-958d-73118e7c1d92	√
3	https://chatgpt.com/share/d2a356e2-e434-4347-b2e5-a8bac0ec8c78	×	https://chatgpt.com/share/a3cb8039-786b-41f1-9479-6e65f12d19fc	×	https://chatgpt.com/share/92f253a7-059e-42f9-9a72-2b592e9f991e	×	https://chatgpt.com/share/d5a82887-d943-4598-899c-6e8d00dfc9ea	√	https://chatgpt.com/share/ee957a31-9a6c-456f-a43a-d79807c16187	√	https://chatgpt.com/share/68d6cfbf-1728-8013-94f6-f473239fd5d3	√
4	https://chatgpt.com/share/ab5e0836-21aa-47d0-b0aa-3d84ec897411	×	https://chatgpt.com/share/e5507979-e4d3-47d4-89ed-988e0139f9f3	×	https://chatgpt.com/share/a4557197-eaa3-4938-8fc1-a60d084ada7e	√	https://chatgpt.com/share/5a0cd1c2-4b0c-44fc-991a-45e267463d69	√	https://chatgpt.com/share/dee1f37e-243b-4eb4-8cf8-70f7b77e6e4f	√	https://chatgpt.com/share/68d6cfce-67a4-8013-bd5a-daf987bed6bb	√
5	https://chatgpt.com/share/032ad895-d565-448e-b92d-	×	https://chatgpt.com/share/fe2d3aac-a212-4d01-9fa2-	√	https://chatgpt.com/share/64d98d63-e46e-4368-adb7-	√	https://chatgpt.com/share/48404ae0-702c-4394-8f85-	√	https://chatgpt.com/share/f96388fc-2bf0-4792-bf10-	√	https://chatgpt.com/share/68d6cfde-2600-8013-a24f-1c5be45f3ddb	√

	e18061751622		8f7d0217ae28		239df991e07b		b0d4d0d7c067		0e080b2c41a2			
6	https://chatgpt.com/share/0be20724-048e-4b55-95be-8f44de82b82e	×	https://chatgpt.com/share/2f7f731f-acc5-48f8-bd91-e51f2eabee01	×	https://chatgpt.com/share/612c20dc-f56b-415e-aa99-e34d0d5e6d17	√	https://chatgpt.com/share/3de8fb1b-b409-44d2-b3ef-ee4074baa021	√	https://chatgpt.com/share/56420ef4-ae19-4b70-8895-1c7fc6049794	√	https://chatgpt.com/share/68d6cff2-54d8-8013-8df7-3a042885e3a4	√
7	https://chatgpt.com/share/278bb4b9-ca3b-41b0-b738-857a7101d679	×	https://chatgpt.com/share/cff2afa6-87c6-4dd8-acec-522d27ed13b5	√	https://chatgpt.com/share/b2137bd3-3e16-4f2f-9990-a409a795bac5	×	https://chatgpt.com/share/fa120b02-2c2c-4c9e-8c86-a40b740cac91	√	https://chatgpt.com/share/5dcff7bc-bc0a-4711-bfde-9aacaa0c4ecc	√	https://chatgpt.com/share/68d6cfff-89f8-8013-a1df-80aeec26a2a4	√
8	https://chatgpt.com/share/075be24f-1375-4d4d-a6ec-5e632ecf699b	√	https://chatgpt.com/share/87e68db9-2e0d-40be-b0e1-08d13bc7e384	√	https://chatgpt.com/share/7bb1eef3-c097-4675-aa50-1466d7a9b340	√	https://chatgpt.com/share/e80cd8f3-f579-4d51-91a2-7d92d3aa8530	√	https://chatgpt.com/share/c50fb108-e604-473e-abd3-4eed0c298f39	√	https://chatgpt.com/share/68d6d02c-35e8-8013-9765-588b49232b3e	√
9	https://chatgpt.com/share/9e55248a-5a15-41cc-8268-7b0bf0870104	×	https://chatgpt.com/share/44ac488f-8b91-418d-b220-906ce08d0cb0	√	https://chatgpt.com/share/ae6d65cb-7daa-47e1-ac56-089687edf7ec	√	https://chatgpt.com/share/4b8399b5-1628-4d99-9bcf-1f444b2bfbde	√	https://chatgpt.com/share/6202fdb9-17c0-4374-8945-cdd2dd8edbb4	√	https://chatgpt.com/share/68d6d04e-ff90-8013-8b74-5cf8f181bc6d	√
10	https://chatgpt.com/share/4ea2609d-c4b2-	×	https://chatgpt.com/share/a6761153-3041-	√	https://chatgpt.com/share/a7fc156f-8b67-	√	https://chatgpt.com/share/9c37937d-c4bc-	√	https://chatgpt.com/share/ca27e4c9-322b-	√	https://chatgpt.com/share/68d6d010-6e4c-8013-	√

	439c-abf5-4c8915281bf5		43c8-82a4-ac02d60a7a98		431e-882b-945c302098c6		4351-a126-3e362bf110d8		4073-8e9e-407d8b04fb04		80c6-d9d30093baec	

Supplementary Table 34: Chat sessions for testing a recent computational virome tool (CheckV).

CheckV	ChatGPT-3.5	Status	ChatGPT-4	Status	ChatGPT-4o	Status	ChatGPT-4 With document	Status	ChatGPT-4o With document	Status	ChatGPT-5	Status
1	https://chatgpt.com/share/a300a9b4-10b4-4a7b-88b7-85ced2cf09c0	√	https://chatgpt.com/share/b6ba49d2-b5cd-4dc0-b0f0-e96089265034	×	https://chatgpt.com/share/0d6c41d5-91b1-4a77-b2d2-bb190ee907e1	√	https://chatgpt.com/share/2384be1f-8072-4468-9c71-5d0273cb1399	√	https://chatgpt.com/share/6f89d877-0f3e-4c6e-9bef-168bfb3bfa66	√	https://chatgpt.com/share/68d6d24b-d2c4-8013-b09a-598f16ddcf82	√
2	https://chatgpt.com/share/a347c33b-35ea-475d-9b00-877958a28997	×	https://chatgpt.com/share/9f433155-11c5-4659-a614-74492e9fd1d6	×	https://chatgpt.com/share/14dec06d-2138-4798-8329-6ff365cbfb88	√	https://chatgpt.com/share/8bd06793-e5a2-4e8d-8da8-eb8284aaf594	√	https://chatgpt.com/share/be88e0eb-75f7-46a2-9f77-526ba58e2ad0	√	https://chatgpt.com/share/68d6d403-44f4-8013-978d-8438e1b4e828	√
3	https://chatgpt.com/share/c17fe13e-3e97-4c1a-8f03-b0aa3b10ffd4	×	https://chatgpt.com/share/4e86fbfd-a42a-4f42-bcda-717be49775c1	×	https://chatgpt.com/share/1650357b-6967-4b47-8f81-26f47c067524	√	https://chatgpt.com/share/e06ad63a-f636-4e33-ada1-67d8e1a32697	√	https://chatgpt.com/share/73814f7f-062a-4d3d-9d72-a8445b825138	√	https://chatgpt.com/share/68d6d444-0048-8013-b655-f228db4bef32	√
4	https://chatgpt.com/share/8d	√	https://chatgpt.com/share/fc9	×	https://chatgpt.com/share/b9	√	https://chatgpt.com/share/7a7	√	https://chatgpt.com/share/aae	√	https://chatgpt.com/share/68d6d4a7-	√

	367a81-2f11-48ed-ae86-9c2edbab990a		20b3f-cbb1-4954-af69-dba62d6201fc		6d6cb0-9b0e-432e-a12e-5822039b4472		5456d-6916-45ce-8fbc-fe66e18309d8		0bee4-2726-491b-974f-820691550161		5df0-8013-bec6-ac8fe429766c	
5	https://chatgpt.com/share/0d9eb407-29c9-426e-96c5-373f12dd1ce5	×	https://chatgpt.com/share/b82794c6-87c1-4b88-89c9-590d7a709944	×	https://chatgpt.com/share/1d5eadff-e342-4680-8d5c-67d30b5f3af7	√	https://chatgpt.com/share/a71ac0c5-6d64-49bf-a04f-b15454533a34	√	https://chatgpt.com/share/38fda9bb-4a70-4c92-ad3a-0eea5a969d75	√	https://chatgpt.com/share/68d6d4e0-18cc-8013-9b90-ff73da894253	√
6	https://chatgpt.com/share/086f4553-f3a9-4ee2-905d-df29ea51bf0c	×	https://chatgpt.com/share/51a74ea1-5dda-44cb-94a1-7783ba9893c7	×	https://chatgpt.com/share/e06df9dd-06dd-49d5-8b06-14ebfd66ee9a	√	https://chatgpt.com/share/a3200cd2-034f-4a4c-afcf-37b1165498c2	√	https://chatgpt.com/share/c36bf5da-a24f-4931-9e58-83794af8252d	√	https://chatgpt.com/share/68d6d504-7de8-8013-b0ad-b2e4614b5a89	√
7	https://chatgpt.com/share/1b563cea-c1c4-4f8c-9e08-ed01368b7e3c	×	https://chatgpt.com/share/25e05d4e-dab9-4788-98dc-452f5d1ad862	×	https://chatgpt.com/share/bc925dba-a357-4cb7-a957-a1f54a3a1147	√	https://chatgpt.com/share/57839826-a00e-40c3-ac2a-6d4db95381e3	√	https://chatgpt.com/share/4c146bd1-8f49-4191-9c0b-bbb2d4ff5b92	√	https://chatgpt.com/share/68d6d551-52e8-8013-bb1f-7186decc4bed	√
8	https://chatgpt.com/share/bdbaf8ca-1fae-46c8-805e-e0f5f1762210	×	https://chatgpt.com/share/e2cddee7-2c67-4480-863d-064c7426c30c	√	https://chatgpt.com/share/4858b25f-f215-428b-9bf1-4a59c7ef8010	√	https://chatgpt.com/share/502a08df-3d47-4bdb-9424-12250c032e0d	√	https://chatgpt.com/share/48f3141b-717e-4ed9-b9cd-a6830f40cb8c	√	https://chatgpt.com/share/68d6d587-54d8-8013-a023-29463369e951	√

9	https://chatgpt.com/share/f9fe4d5a-472a-47a9-a317-c3e019d456e1	√	https://chatgpt.com/share/57019991-ca4a-4b27-8a29-6631d72c66fc	×	https://chatgpt.com/share/0b6086d7-db54-4774-9b33-687e08de5cda	√	https://chatgpt.com/share/213db613-2631-45c2-9217-8884c6a5b3a1	√	https://chatgpt.com/share/c8845e80-ee01-47c0-a4a8-292fc82fa77e	√	https://chatgpt.com/share/68d6d5c2-6e20-8013-b125-5ff1ea4a7b32	√
10	https://chatgpt.com/share/ccbf9723-ad58-4ced-91ff-462c8ada6b7f	×	https://chatgpt.com/share/a2a96924-0ccf-482c-96c3-7ad0531a7d29	×	https://chatgpt.com/share/c71251c6-87ef-4b91-b24d-69455890e031	√	https://chatgpt.com/share/c7e5ff23-1f99-4b02-b216-af429f10192c	√	https://chatgpt.com/share/5de9e0e3-a735-43d9-ae47-01f2225a0897	√	https://chatgpt.com/share/68d6d604-d0f4-8013-a8be-e77e6326f595	√

Supplementary Table 35: Chat sessions for testing a recent computational virome tool (vRhyme).

vRhyme	ChatGPT-3.5	Status	ChatGPT-4	Status	ChatGPT-4o	Status	ChatGPT-5	Status
1	https://chatgpt.com/share/2bf60031-cf0c-430d-b39a-27a00a9174cc	×	https://chatgpt.com/share/8b57a321-a973-42f9-8e56-b790406674d9	√	https://chatgpt.com/share/3fcc7924-810b-4205-a739-a6ba56fe3056	√	https://chatgpt.com/share/68d6d7b0-c4ec-8013-bd9d-34afef4d9980	√
2	https://chatgpt.com/share/fb9bfdd7-251e-4624-a816-dbd044e147a8	×	https://chatgpt.com/share/bed37397-2de7-4a3d-b278-23829f690b8a	√	https://chatgpt.com/share/98aec3b1-3771-4b5d-8be9-d4e0c6c94773	√	https://chatgpt.com/share/68d6dc64-c800-8013-b5d9-d086a2ce6bea	√
3	https://chatgpt.com/share/e08ad443-8648-4da2-ade0-24bb71bc0368	×	https://chatgpt.com/share/c6ae9cda-e232-443b-8c8a-b95970535c1c	√	https://chatgpt.com/share/07fb5a25-d3fd-4fec-9609-e11468ee209f	√	https://chatgpt.com/share/68d6dca7-e43c-8013-8d70-ef10f25981c2	√
4	https://chatgpt.com/share/54381739-2750-43cb-	×	https://chatgpt.com/share/7fe40d36-3252-41fd-	√	https://chatgpt.com/share/705f0d4c-55b1-4891-	√	https://chatgpt.com/share/68d6dce0-8098-8013-	√

	8e94-0b4c7c8fea43		81d5-98b7c7f0765e		b14c-8347fdc348d8		aa22-5bf2f1542e5e	
5	https://chatgpt.com/share/45a4e88a-9571-4160-a74d-0d2560fb9431	×	https://chatgpt.com/share/6d27bcf0-9a4b-4211-bee5-e7adf4796cbc	√	https://chatgpt.com/share/81f6bfbe-d351-427f-a66e-76da15951baa	√	https://chatgpt.com/share/68d6dd1a-88e0-8013-bc4c-bb515b882075	√
6	https://chatgpt.com/share/1d1624c5-a45b-43e3-96b1-378852b9f1be	×	https://chatgpt.com/share/c4976fb4-3391-45a3-9b3a-b0c753e66ad8	√	https://chatgpt.com/share/c7eb889f-d133-48b6-bf90-c335982d9596	√	https://chatgpt.com/share/68d6dd8b-5574-8013-b3e0-80ecffa415ad	√
7	https://chatgpt.com/share/38013289-0789-4bd4-95ac-3f3adee5bd7d	×	https://chatgpt.com/share/aae9b172-9466-4e9a-b808-cb286cbe131d	√	https://chatgpt.com/share/acbf0072-7c15-4130-86bd-64cb203928fb	√	https://chatgpt.com/share/68d6ddd8-2a88-8013-8ce1-5421dbd92787	√
8	https://chatgpt.com/share/4ef4cf46-3058-4351-8272-4f28dec9689f	×	https://chatgpt.com/share/e47d27d3-1a2f-46e9-ad82-9e6e87055784	√	https://chatgpt.com/share/5b3d4013-dd76-4813-acef-7d1267934918	√	https://chatgpt.com/share/68d6de14-a384-8013-a2e8-4c75910851c4	√
9	https://chatgpt.com/share/b206b9db-0870-4634-8526-9f5ab2b16f6d	×	https://chatgpt.com/share/f548b693-28b9-490a-8255-b589225213b8	√	https://chatgpt.com/share/d7668c75-91b9-4e95-8e44-0fb6df6572fe	√	https://chatgpt.com/share/68d6de79-9c44-8013-9b39-cc86ff227d90	√
10	https://chatgpt.com/share/2abec359-1a96-4162-b289-494b6a700c4f	×	https://chatgpt.com/share/26534ac1-f4db-420f-96ca-c95bcf23783c	√	https://chatgpt.com/share/98f02b03-b862-4258-8d29-f6ce9cc1e084	√	https://chatgpt.com/share/68d6decf-99a8-8013-97c4-e3cda8eba28f	√

Supplementary Table 36: Chat sessions for testing a recent computational virome tool (PHAMB).

PHAMB	ChatGPT-3.5	Status	ChatGPT-4	Status	ChatGPT-4o	Status	ChatGPT-5	Status
1	https://chatgpt.com/share/2f897d71-03e1-4718-	×	https://chatgpt.com/share/10f08828-e53d-46bf-	×	https://chatgpt.com/share/e1b7e468-ce28-428b-	√	https://chatgpt.com/share/68d6dfaf-fec0-8013-	√

	be7d-46921b12b7d4		a800-827eb25fa15f		9b98-1fbb3b57f92f		ac37-f6d9ea7062d3	
2	https://chatgpt.com/share/0e01af88-428c-46ab-aa93-2eae46d89e3b	×	https://chatgpt.com/share/ce26a5ba-79a1-48d3-b0c4-fd72a7adb093	×	https://chatgpt.com/share/4abad54d-b900-4364-9a71-d01fe958ed74	√	https://chatgpt.com/share/68d6e005-d7bc-8013-9401-fb53dc0c7dfd	√
3	https://chatgpt.com/share/71ec3d10-64e9-4c73-9e32-1086f8ad2856	×	https://chatgpt.com/share/c7f40b64-5aa7-4f04-97a7-94dcd8fc51b7	√	https://chatgpt.com/share/d8d2a432-1b4c-4a7b-875e-acf714e48cb8	√	https://chatgpt.com/share/68d6e046-19e4-8013-b359-522bb802aff2	√
4	https://chatgpt.com/share/f19ef840-da7f-4829-bbc1-9250242ec6fb	×	https://chatgpt.com/share/dc17fea2-d391-44d5-a212-30f3afd0de3c	×	https://chatgpt.com/share/4bcd646f-e7c7-40fc-ab6f-bca1bb75bf8f	√	https://chatgpt.com/share/68d6e0a1-c170-8013-b760-2de183834b34	√
5	https://chatgpt.com/share/e3e72aa2-eabf-4bf0-be01-60d3b55958b1	×	https://chatgpt.com/share/5d6a3c77-9356-4da4-a879-d665aa3bf63a	×	https://chatgpt.com/share/9200991a-da24-4a9b-9971-bf2eee6bca7d	√	https://chatgpt.com/share/68d6e0ec-1ca0-8013-b92e-794636a50d84	√
6	https://chatgpt.com/share/3f7f0bee-ea6f-4495-8046-49bbe3ee5593	×	https://chatgpt.com/share/852a01ec-cb12-42e7-b2b5-eff44d4e95fe	×	https://chatgpt.com/share/cd7000f5-5d9f-4fb1-890d-4bf821a99424	√	https://chatgpt.com/share/68d6e15e-9fb4-8013-b247-8b2f265e2587	√
7	https://chatgpt.com/share/cd1c3a77-8ae5-4e83-91e5-e46f59b15036	×	https://chatgpt.com/share/833b2ee3-8d29-4bbf-a9ef-b95da2ba2f0a	×	https://chatgpt.com/share/1004729f-b1be-421c-b09d-dc8814970caf	√	https://chatgpt.com/share/68d6e1e4-20e8-8013-97d1-dd6e014b4fd3	√
8	https://chatgpt.com/share/caaded12-e235-455b-bd9c-88a71d7a30e8	×	https://chatgpt.com/share/a5276522-de96-4183-bf41-04757081bd5f	√	https://chatgpt.com/share/b6112623-37e4-4a09-838f-c9987e5e0315	√	https://chatgpt.com/share/68d6e23e-0cd4-8013-8bf2-a7539a39b64c	√
9	https://chatgpt.com/share/8b4fe9bc-1dd2-45d9-	×	https://chatgpt.com/share/c96edd78-8e01-4a33-	√	https://chatgpt.com/share/d6b6bc41-459d-4b4d-	√	https://chatgpt.com/share/68d6e276-07d0-8013-	√

	8a64-9e5892170298		bbf1-72c63e4618af		b560-1940196f0aad		850b-5f5317818c97	
10	https://chatgpt.com/share/cf576dc3-308a-49c4-ae59-e2bc13c72f80	×	https://chatgpt.com/share/f4375f2a-26f1-4b05-9655-226372cb9833	√	https://chatgpt.com/share/20eabe70-47cf-4864-bf0c-0a5aaf107083	√	https://chatgpt.com/share/68d6e2ba-8974-8013-b5b7-eefad07f7ae6	√

Supplementary Table 37: Chat sessions for testing a recent computational virome tool (geNomad).

geNomad	ChatGPT-3.5	Status	ChatGPT-4	Status	ChatGPT-4o	Status	Chatgpt-5	Status
1	https://chatgpt.com/share/9700ec9a-def5-49d0-bb21-272684ade54d	×	https://chatgpt.com/share/bd9dd223-5de3-4854-b14f-ba284adcb6c9	√	https://chatgpt.com/share/3d6bf15f-1a1c-4750-b9de-cc38a4f9272e	√	https://chatgpt.com/share/68d6ebe8-c8a8-8013-99cb-fed63cf108f3	√
2	https://chatgpt.com/share/9911da8c-29f9-4133-9f44-c382c8676860	×	https://chatgpt.com/share/c6b7c81f-d419-4209-a011-58b700ef855d	×	https://chatgpt.com/share/32b18b08-0071-448c-8693-1969549b184f	√	https://chatgpt.com/share/68d6eb96-826c-8013-addc-c1fd30c6152e	√
3	https://chatgpt.com/share/c40bd8e1-16d5-4481-9a2a-688d7aa785f2	×	https://chatgpt.com/share/da9d75ec-55e8-4a5b-85a4-b910c1480ce2	×	https://chatgpt.com/share/88167492-627a-4535-bd2e-19b2fd507c41	√	https://chatgpt.com/share/68d6eb43-97ec-8013-92d1-8c40c6642276	√
4	https://chatgpt.com/share/f3043e78-8beb-4652-b2b0-e795ac432a3a	×	https://chatgpt.com/share/9b1f88ce-373f-40ee-858b-3ddf19fd2cd6	√	https://chatgpt.com/share/3dd4e2ee-dd06-45d1-a797-9537b9bc5a3d	×	https://chatgpt.com/share/68d6eaa1-ca40-8013-9981-79d34456056c	√
5	https://chatgpt.com/share/17f95b92-254a-4083-9e92-6a6965db5785	×	https://chatgpt.com/share/bf715164-a426-4674-a44b-f9ffd5e0dd5d	√	https://chatgpt.com/share/4cf5f6c5-35c4-4abc-aa30-1dfd2641f39d	√	https://chatgpt.com/share/68d6e90b-bb88-8013-adf0-239eae801500	√
6	https://chatgpt.com/share/455cca0d-9130-4ced-9129-310c6e99bacb	×	https://chatgpt.com/share/9ce52fde-6741-4fa4-9ec1-68de0adbd4fe	√	https://chatgpt.com/share/43fa8a83-458d-49db-98e1-ff90ade1feb3	√	https://chatgpt.com/share/68d6e8bf-ff44-8013-9730-b0ee773346e5	√

7	https://chatgpt.com/share/98fca111-e3e6-465e-8bd8-6b1e4c4d8448	×	https://chatgpt.com/share/0b5fd517-520e-43e0-b075-de1e4c40f6f7	√	https://chatgpt.com/share/7572cca1-5198-49e3-8937-bf319f63cb89	√	https://chatgpt.com/share/68d6e875-152c-8013-add7-96c5cebf80c3	√
8	https://chatgpt.com/share/a1d6684d-2d6a-463d-b39f-9d38a5fea8ed	×	https://chatgpt.com/share/9c15cdf2-d77a-412d-a84c-35c189d6ff7c	√	https://chatgpt.com/share/ac5a04b4-2108-4262-b67f-9001226ec90b	√	https://chatgpt.com/share/68d6e836-e810-8013-892c-644de205b683	√
9	https://chatgpt.com/share/2e05e044-fba0-4ba1-9a39-fb0855bc09e2	×	https://chatgpt.com/share/ca7329d5-d7b8-4e57-ad85-4eaf603c50da	√	https://chatgpt.com/share/f4d2bf7a-83e9-4a7b-bc32-7feee53bd1ef	√	https://chatgpt.com/share/68d6e7ff-eca0-8013-831a-126b291b381f	√
10	https://chatgpt.com/share/b6b6d304-e12c-4dd9-84e5-2ca8c6219553	×	https://chatgpt.com/share/fe5c189c-309d-4962-82fe-d8b19fa28b38	×	https://chatgpt.com/share/02960073-702a-437b-adca-b4ba6665979c	√	https://chatgpt.com/share/68d6e352-d7e4-8013-9927-cf5299a403b3	√

Supplementary Table 38: Chat sessions for testing a recent computational virome tool (iPHoP).

iPHoP	ChatGPT-3.5	Status	ChatGPT-4	Status	ChatGPT-4o	Status	ChatGPT-5	Status
1	https://chatgpt.com/share/b588553a-6f39-44be-8832-b37be4db24c1	×	https://chatgpt.com/share/6b1a0dc8-a02a-4a46-8445-aa504e43e028	√	https://chatgpt.com/share/4431890d-e5cb-4bcc-ad0f-b638c6d1e699	√	https://chatgpt.com/share/68d6ecbd-e344-8013-9bb2-e590edd6da8c	√
2	https://chatgpt.com/share/1865e5fb-0b2a-42d8-bd8a-f7fe3fe8cf34	×	https://chatgpt.com/share/7d005c6e-0b8a-4ea4-9c15-a532537655f5	√	https://chatgpt.com/share/44bf52a5-f388-422c-a057-9fd4b662ce08	√	https://chatgpt.com/share/68d6ed0f-4ec4-8013-ac68-9aa861078a1c	√
3	https://chatgpt.com/share/389b7ffa-a372-42be-8ad4-3162e095bf96	×	https://chatgpt.com/share/c2ea2ab9-7700-4b3c-85e1-8a3bd3424600	√	https://chatgpt.com/share/dc6ba046-d43a-4224-b4b3-8c49c728de04	√	https://chatgpt.com/share/68d6ed4e-06c0-8013-870f-d43d11b71ce8	√
4	https://chatgpt.com/share/7350682	×	https://chatgpt.com/share/5b79a07	√	https://chatgpt.com/share/e9f3a06	√	https://chatgpt.com/share/68d6ed8	√

	0-8565-4ace-a068-dbfc1a53ada9		2-795b-418b-a794-61d90407af2d		e-93e6-4256-ad88-7f0a3b96bf3a		b-462c-8013-a902-c7347dd1771f	
5	https://chatgpt.com/share/6d95cbd9-0f9e-4e33-92ad-4944756127b1	×	https://chatgpt.com/share/714003af-8221-4019-bc51-db61e1da57c9	√	https://chatgpt.com/share/b6e27cb2-a5e6-4016-b4dd-fc07bd6245e7	√	https://chatgpt.com/share/68d6edd6-5560-8013-a578-bb5a4e2c1ba8	√
6	https://chatgpt.com/share/3d86031a-fe0a-43d1-aa07-06d006c473da	×	https://chatgpt.com/share/d57f9c6a-73ba-4e24-99ba-e309c38c70fa	√	https://chatgpt.com/share/8e14fb89-34bf-4b3f-b8ce-8b73b2fd17bb	√	https://chatgpt.com/share/68d6ee4c-3720-8013-9d85-b97692eae51a	√
7	https://chatgpt.com/share/f7960f95-1627-4296-8b25-bf2bfe1e4382	×	https://chatgpt.com/share/6622e20f-fc90-40ce-bdc9-e74b05c15ce4	√	https://chatgpt.com/share/89c19554-f362-474c-9879-aff003b8b9e0	√	https://chatgpt.com/share/68d6eeac-bddc-8013-8b10-e0fddbddb1af	√
8	https://chatgpt.com/share/057c6146-f32b-45cd-8627-e4a7bdcfc1fc	×	https://chatgpt.com/share/9fab4059-7cc2-42cb-afae-f9f9acf1bd52	√	https://chatgpt.com/share/e28b19c4-0c4c-4801-b4d3-499de7324a5c	√	https://chatgpt.com/share/68d6eff1-1e84-8013-803a-852fb70f3ab7	√
9	https://chatgpt.com/share/a0f85afb-185b-48d0-9c07-24cfe9d276db	×	https://chatgpt.com/share/b8df0026-ed96-4824-a7c2-7ecbbd5dbe37	√	https://chatgpt.com/share/e62a21f0-65ce-4e72-8117-3be9a00c8904	√	https://chatgpt.com/share/68d6f27e-fb40-8013-9f87-796705d40506	√
10	https://chatgpt.com/share/7c0870df-1eb2-4b24-ba94-11397788d163	×	https://chatgpt.com/share/847ffac4-1ea0-45a5-9c2d-cb4923491e92	√	https://chatgpt.com/share/87c9c225-df44-4424-9b1d-a0b43992bdee	√	https://chatgpt.com/share/68d6f2bd-6874-8013-a4db-ed2dea886cc1	√

Supplementary Table 39: Pipeline tool replacement testing (geNomad replacing checkV) For ChatGPT-4omini, the status sign means correctness without providing document. For ChatGPT-4 and ChatGPT-4o, the first status sign means correctness without providing document, and the second status sign means correctness with providing document.

geNomad	ChatGPT-4o mini	status	ChatGPT-4	Status	ChatGPT-4o	Status

1	https://chatgpt.com/share/8292405c-250d-4de8-8484-48bc24e35848	×	https://chatgpt.com/share/6b1663c4-4a67-4824-9188-0231f0eacd3b	×√	https://chatgpt.com/share/6fb8515f-7e8c-4001-b7cb-ac4ca1fc90ef	×√
2	https://chatgpt.com/share/f1bfdf28-305d-4469-88d5-ee8364f7879b	×	https://chatgpt.com/share/166143bc-aeb3-4ae9-a537-156c43697987	×√	https://chatgpt.com/share/1a13d7c4-0f62-4258-9fe8-ad876146f09a	×√
3	https://chatgpt.com/share/f633acc2-fa9b-404d-a06d-8cc7c86c5d53	×	https://chatgpt.com/share/2c774dfa-247c-4a47-9d23-bdbcb5564f0b	×√	https://chatgpt.com/share/5db16d52-cf1f-4973-b302-8acbf5f30376	×√
4	https://chatgpt.com/share/09705dfe-21f3-4d10-9afb-53fcd50f9a29	×	https://chatgpt.com/share/484daaf7-4210-4286-a91d-114ef7a8c25c	×√	https://chatgpt.com/share/f8b0422a-dded-40b9-a746-77f739079c50	×√
5	https://chatgpt.com/share/5c8c7b21-b2d3-474b-a620-900a54aa81c4	×	https://chatgpt.com/share/982d3ab2-a04e-4b98-bc2f-43daff8f8cb3	×√	https://chatgpt.com/share/b3640334-d901-4913-b667-17ef52ed291e	×√
6	https://chatgpt.com/share/12ea5e4c-1b66-4e78-8528-de5a1d0dc1b9	×	https://chatgpt.com/share/a41c1577-8f77-4638-9524-f7df06f960cd	×√	https://chatgpt.com/share/7dd28b88-2925-409b-b0c8-adac9747f9b3	×√
7	https://chatgpt.com/share/00e9bad0-c7a2-4d03-b0d5-122f9b3a23c1	×	https://chatgpt.com/share/83b9e31d-5d02-4bd9-b6a2-14a7d772d363	×√	https://chatgpt.com/share/1473bcde-0a38-4a38-a8f4-3cfeb3feb3c0	×√
8	https://chatgpt.com/share/45df3d55-d819-4ecd-8969-740cf010ecf4	×	https://chatgpt.com/share/589d1a87-905c-4ac2-b35b-8df2bff60876	×√	https://chatgpt.com/share/0a75669f-d876-4846-9566-ec88570839ee	×√
9	https://chatgpt.com/share/76c3252b-6802-412b-9e36-e3b2ae7bcb76	×	https://chatgpt.com/share/522c1e6d-5a51-440b-860c-a719020f3d1c	×√	https://chatgpt.com/share/964fb1f1-7340-4f5e-ba5f-5e921ecf73b1	×√
10	https://chatgpt.com/share/4d4e3b38-fc25-4c8c-bc15-46cf192bd202	×	https://chatgpt.com/share/e6dd0669-4937-4f22-958a-e17a09490e30	×√	https://chatgpt.com/share/dca3f30c-093d-4a70-8757-ec8d1af7e13a	×√

Supplementary Table 40: Pipeline tool replacement testing (VirSorter2 replacing checkV). For ChatGPT-4omini, the status sign means correctness without providing document. For ChatGPT-4 and ChatGPT-4o, the first status sign means correctness without providing document, and the second status sign means correctness with providing document.

Virsorter2	Chatgpt-4omini	Status	ChatGPT-4	Status	ChatGPT-4o	Status

1	https://chatgpt.com/share/ced0ec91-ebb7-44c6-8ae0-9953ccf8408d	×	https://chatgpt.com/share/b9766936-cb5d-4878-92c8-5aaf8c9b5180	×√	https://chatgpt.com/share/057acc27-24e1-4c78-9314-5bf929ef8ab1	×√
2	https://chatgpt.com/share/da9c508d-5081-44fd-81f2-71a8845e0f4c	×	https://chatgpt.com/share/90ae1f79-2f20-4e64-89a1-78cbabf3da72	×√	https://chatgpt.com/share/925abdaf-12fc-4f9d-be29-5089292b2fe1	×√
3	https://chatgpt.com/share/8a7054d4-cce3-4c87-ad52-ad60191a4fac	×	https://chatgpt.com/share/48d68791-fa1a-484d-9439-51737233bc33	××	https://chatgpt.com/share/463e8a88-95cd-4c0c-a79e-b527695ef38c	√√
4	https://chatgpt.com/share/632ddea7-b17e-4f39-beb7-e0abb4cf9637	×	https://chatgpt.com/share/fe64f2e4-f33d-4174-aa45-a6e5b7cec199	×√	https://chatgpt.com/share/f338c15d-67c8-483e-a4a5-cdf2c3fb82b9	×√
5	https://chatgpt.com/share/a0533d6f-0bee-4397-b94b-952472afa395	×	https://chatgpt.com/share/79c5c9f0-00fc-43d6-a7e4-4957e617a783	××	https://chatgpt.com/share/2c57649d-5048-407a-b86d-ea79c395cf7e	×√
6	https://chatgpt.com/share/8d4b0e9a-e5ea-4878-952b-cd91d7288230	×	https://chatgpt.com/share/0bf79a58-6765-4ba2-876d-fa12856b14af	××	https://chatgpt.com/share/8af1ff89-45c4-4136-b72e-67c1ad206412	√√
7	https://chatgpt.com/share/f3f1aff8-d3b8-449e-8422-c9b4db250fee	×	https://chatgpt.com/share/635446dc-457e-41f5-a829-31783ef97003	√√	https://chatgpt.com/share/9b489143-d998-46b0-9d56-5a7cfa17dde7	×√
8	https://chatgpt.com/share/4ea7834a-9bce-40ac-b4b0-64f8a77dd5c2	×	https://chatgpt.com/share/fe10f0ee-7404-4735-9ccc-8acd1b2e65e5	×√	https://chatgpt.com/share/dec3170e-1b06-4492-87c5-031c5d25c2a5	√√
9	https://chatgpt.com/share/083384d9-acb2-4a21-8311-5ad551193232	×	https://chatgpt.com/share/187fe9d1-2fb3-4788-b56a-78f7baeaef29	×√	https://chatgpt.com/share/c8f571b2-ac8f-4d26-a6b2-803a6fab0189	√√
10	https://chatgpt.com/share/19cb0205-b0c8-49a6-a5da-e31d569ace3e	×	https://chatgpt.com/share/b9e731d3-4a95-4b29-b23d-7b75b24a5328	×√	https://chatgpt.com/share/76dfd802-2a70-45a6-8936-08e9e33c3e64	√√

Supplementary Table 41: Pipeline tool replacement testing (geNomad replacing checkV) For ChatGPT-o1-mini and ChatGPT-o1-preview, the status sign means correctness without providing document.

geNomad	ChatGPT-o1-mini	Status	ChatGPT-o1-preview	Status
1	https://chatgpt.com/share/66e8a801-8b44-8013-80c3-1ade1a6d6d72	×	https://chatgpt.com/share/66e8a759-e7a4-8013-bcd5-1d3ab8440641	×
2	https://chatgpt.com/share/66e8a811-71a4-8013-a3ea-2fe92b0a731d	×	https://chatgpt.com/share/66e8a768-b534-8013-af17-2ce75d7e4015	×

3	https://chatgpt.com/share/66e8a820-a564-8013-97e5-28a9cd64efe6	×	https://chatgpt.com/share/66e8a77c-2fdc-8013-ba3f-d8c33b4a4dc8	×
4	https://chatgpt.com/share/66e8a830-a700-8013-baaf-0fe70d69fdb4	×	https://chatgpt.com/share/66e8a78b-d584-8013-bb52-92534b948306	×
5	https://chatgpt.com/share/66e8a842-2a40-8013-891c-1978df922858	×	https://chatgpt.com/share/66e8a797-6f50-8013-a9cf-42488101e89e	×
6	https://chatgpt.com/share/66e8a850-5cc8-8013-ab2f-e15cf87a9214	×	https://chatgpt.com/share/66e8a7a4-9980-8013-8830-7a759803efc6	×
7	https://chatgpt.com/share/66e8a85c-81c8-8013-af02-3850b38048be	×	https://chatgpt.com/share/66e8a7b0-3cb8-8013-aebc-ea8b3368a5c7	×
8	https://chatgpt.com/share/66e8a868-8494-8013-8151-a5fa1523d381	×	https://chatgpt.com/share/66e8a7be-387c-8013-8e83-5f3b65a3a360	×
9	https://chatgpt.com/share/66e8a875-bfd8-8013-a507-056f08926a9a	×	https://chatgpt.com/share/66e8a7c9-d8b8-8013-a313-0b1d97459a3b	×
10	https://chatgpt.com/share/66e8a87f-cb4c-8013-80b4-15c82807e1a0	×	https://chatgpt.com/share/66e8a7d8-158c-8013-9054-b6d5cc211670	×

Supplementary Table 42: Pipeline tool replacement testing (VirSorter2 replacing checkV) For ChatGPT-o1-mini and ChatGPT-o1-preview, the status sign means correctness without providing document.

VirSorter 2	ChatGPT-o1-mini	Status	ChatGPT-o1-preview	Status
1	https://chatgpt.com/share/66e99231-4e84-8013-8a3a-545de84f4559	×	https://chatgpt.com/share/66e99c81-d950-8013-96c0-6b322d13d14f	√
2	https://chatgpt.com/share/66e99241-e44c-8013-bb4d-e91315544664	×	https://chatgpt.com/share/66e99c8f-c144-8013-a5e8-458fd8d35538	√
3	https://chatgpt.com/share/66e99257-a9f4-8013-898f-e97832fc33b4	×	https://chatgpt.com/share/66e99c9d-d0f4-8013-9d38-6e815d1c8916	√
4	https://chatgpt.com/share/66e9926d-b0f4-8013-bc16-eb5eb6257e60	×	https://chatgpt.com/share/66e99cb2-3d88-8013-a35e-7962b5c57cf6	√
5	https://chatgpt.com/share/66e99282-a470-8013-b116-7835b5071e73	×	https://chatgpt.com/share/66e99cc6-8174-8013-8841-db6b9c9c98ca	√
6	https://chatgpt.com/share/66e9929d-d914-8013-b7cd-e300d07c6b1d	×	https://chatgpt.com/share/66e99cd3-2e08-8013-9034-0fcd010d7619	√
7	https://chatgpt.com/share/66e992b0-3568-8013-b831-57b34ef556b0	×	https://chatgpt.com/share/66e99ce0-aad0-8013-b192-0b12f2d06c78	√
8	https://chatgpt.com/share/66e992c4-a298-8013-a73c-f8e6888cd322	×	https://chatgpt.com/share/66e99cec-4990-8013-ae3f-ae5bff521db0	√
9	https://chatgpt.com/share/66e992d5-0440-8013-8590-784dda20f492	×	https://chatgpt.com/share/66e99cf8-57cc-8013-ba02-20b472ac8ac8	√
10	https://chatgpt.com/share/66e992ef-bbe4-8013-85fd-0015044910ee	×	https://chatgpt.com/share/66e99d06-75c0-8013-a8fb-b85336bf5ed8	√

Supplementary Table 43: Pipeline tool replacement testing (geNomad replacing checkV) For ChatGPT-4.1, ChatGPT-4.1-mini and ChatGPT-4.5, the status sign means correctness without providing document.

geNomad	ChatGPT-4.1	Status	ChatGPT-4.1-mini	Status	ChatGPT-4.5	Status
1	https://chatgpt.com/share/682cc826	×	https://chatgpt.com/share/682cd127	×	https://chatgpt.com/canvas/shared/6836297ce6608191b30acb37a6acc1c2	√

	-b6f4-8013-a254-3034c9d3daab		-9ae4-8013-bc15-3b3fbd7b76b7			
2	https://chatgpt.com/share/682ccac6-50a8-8013-ac70-efa04a97bb0d	×	https://chatgpt.com/share/682cd185-8bd0-8013-b0a0-825a638cf57f	×	https://chatgpt.com/canvas/shared/68362a15a4d88191bbbd3362ab0f5a3f	√
3	https://chatgpt.com/share/682ccc6a-02d4-8013-96d4-499d45061847	√	https://chatgpt.com/share/682cd1f6-4d78-8013-9bcd-5d904878650b	×	https://chatgpt.com/canvas/shared/68362a5f6c548191adf55c41a5cf5b1a	√
4	https://chatgpt.com/share/682cce4f-00e8-8013-9191-11e4a645bfef	×	https://chatgpt.com/share/682cd21d-0a14-8013-93d5-ed0ac5766aa9	×	https://chatgpt.com/canvas/shared/68362ad9e43c8191853a9ec8dc2532e8	√
5	https://chatgpt.com/share/682cce75-bcac-8013-a696-79ada36a5d64	×	https://chatgpt.com/share/682cd244-4d8c-8013-b67f-e82ed1c886c9	×	https://chatgpt.com/canvas/shared/68362b454a588191a2d68a523e0fff5f	√
6	https://chatgpt.com/share/682ccf70-432c-8013-90df-cc60c6a7780b	√	https://chatgpt.com/share/682cd27b-adc0-8013-a674-5a348e669225	×	https://chatgpt.com/canvas/shared/68362bb251948191935585a6802142b3	√
7	https://chatgpt.com/share/682ccfc7-72cc-8013-86b4-3a064f17c9ed	×	https://chatgpt.com/share/682cd2a0-4f10-8013-8f71-89adcf24354e	×	https://chatgpt.com/share/6839c14d-58d0-8013-98eb-db0062ab9e81	√
8	https://chatgpt.com/share/682ccffc-bf00-8013-93d6-9f7d6694502c	×	https://chatgpt.com/share/682cd2b9-a090-8013-9dbe-b1958a2e9577	×	https://chatgpt.com/canvas/shared/68362cd4202c8191886ae83294c5f852	√
9	https://chatgpt.com/share/682cd02f-2744-8013-b955-0bf1c36088ea	√	https://chatgpt.com/share/682cd2e5-3590-8013-9d43-b3df34117ed7	×	https://chatgpt.com/canvas/shared/68362d1c22b081918074e9227c6347e9	√
10	https://chatgpt.com/share/682cd067-90b0-8013-a0fc-044647da9eab	×	https://chatgpt.com/share/682cd306-21f0-8013-ba27-84b5aa3f94a8	×	https://chatgpt.com/canvas/shared/6839c1a992888191b6758ce5f3e0edcd	√

Supplementary Table 44: Pipeline tool replacement testing (VirSorter2 replacing checkV) For ChatGPT-4.1, ChatGPT-4.1-mini and ChatGPT-4.5, the status sign means correctness without providing document.

VirSorter2	ChatGPT-4.1	Status	ChatGPT-4.1-mini	Status	ChatGPT-4.5	Status
1	https://chatgpt.com/share/682cd4d1-bdd4-8013-a7aa-72543ccca63c	×	https://chatgpt.com/share/682cd8fe-924c-8013-84c0-cf52a80a6557	√	https://chatgpt.com/canvas/shared/68362ea64b0c8191a44b7895c497d38a	√

2	https://chatgpt.com/share/682cd52d-d42c-8013-8065-4de0bfd5583b	×	https://chatgpt.com/share/682cd92c-134c-8013-9045-ac0461418af8	√	https://chatgpt.com/canvas/shared/68362ef7199c8191a34e4b29515014b4	√
3	https://chatgpt.com/share/682cd5ca-6adc-8013-b12e-2fb4087e9efa	×	https://chatgpt.com/share/682cdb29-e028-8013-b886-8f654355f4cb	√	https://chatgpt.com/canvas/shared/6838c612693c8191b0aa10655bb08104	√
4	https://chatgpt.com/share/682cd5f4-cb60-8013-9d8c-dda7a2246ce6	×	https://chatgpt.com/share/682cdc1b-90f8-8013-970a-6945d77641e4	√	https://chatgpt.com/share/6838c84f-4c00-8013-bebc-5fa7ee360192	×
5	https://chatgpt.com/share/682cd62e-105c-8013-9dc7-8a619015569f	×	https://chatgpt.com/share/682cdc44-b624-8013-afd9-30da9a3399dd	×	https://chatgpt.com/canvas/shared/6838c8dc7d188191a6c6af1fac1ae5bf	×
6	https://chatgpt.com/share/682cd65f-dfc0-8013-bc04-b274d583a38c	×	https://chatgpt.com/share/682cdccd-0778-8013-b465-450904987bc1	×	https://chatgpt.com/canvas/shared/6838c97a7fac8191b201489fd33a2bb1	√
7	https://chatgpt.com/share/682cd6fd-f954-8013-bf8f-685530055a10	×	https://chatgpt.com/share/682cddc8-1e48-8013-8834-0fddf9bc5f87	×	https://chatgpt.com/canvas/shared/6838c9b0f6908191946df760dd2aa993	√
8	https://chatgpt.com/share/682cd72c-003c-8013-963c-9d303f1d6595	√	https://chatgpt.com/share/682cde49-38f0-8013-b31c-7d52c4e9a470	√	https://chatgpt.com/canvas/shared/6838ca58b2a08191aeb3e49c7926a118	√
9	https://chatgpt.com/share/682cd761-b6c0-8013-9308-c4d9399a32d0	×	https://chatgpt.com/share/682cdf7d-cc5c-8013-9996-e4268eaffcf6	×	https://chatgpt.com/canvas/shared/6838ca862e208191be8a3626d8fb0bb3	√
10	https://chatgpt.com/share/682cd8a3-969c-8013-ad4a-483a83daeb84	√	https://chatgpt.com/share/682cdfa7-1584-8013-8962-da287610f55c	×	https://chatgpt.com/share/6838cab4-15b8-8013-aed8-b0814d3e521d	√

Supplementary Table 45: Pipeline tool replacement testing (geNomad replacing checkV) For ChatGPT-5, the status sign means correctness without providing document.

geNomad	ChatGPT-5	Status
1	https://chatgpt.com/share/68bee75c-f638-8013-9e84-d2702e30e444	√
2	https://chatgpt.com/share/68beefb2-86d4-8013-be82-f92e8225150d	√
3	https://chatgpt.com/share/68beefc4-434c-8013-94aa-6889aa0a0b72	√
4	https://chatgpt.com/share/68bef0d5-2948-8013-b3b7-fc2499dec0bf	√
5	https://chatgpt.com/share/68bef1a3-4a6c-8013-a9b9-a0bc09e90bd5	√
6	https://chatgpt.com/share/68bef24b-ede0-8013-859f-1d46d7145a77	√
7	https://chatgpt.com/share/68bef323-ba48-8013-8042-f9fee8f1767b	√
8	https://chatgpt.com/share/68bef4f0-f540-8013-bee8-a22790b2cf0b	√

9	https://chatgpt.com/share/68bef5dd-4020-8013-9854-ae22c7ee31bf	√
10	https://chatgpt.com/share/68bef7f9-5ec8-8013-a217-33768d25e86b	√

Supplementary Table 46: Pipeline tool replacement testing (VirSorter2 replacing checkV) For ChatGPT-5, the status sign means correctness without providing document.

VirSorter 2	ChatGPT-5	Status
1	https://chatgpt.com/share/68befa14-558c-8013-b474-288bffa46b60	√
2	https://chatgpt.com/share/68befaf8-4d40-8013-a6be-4b19bb904b34	√
3	https://chatgpt.com/share/68befb73-5d58-8013-ac0e-e545aea2be73	√
4	https://chatgpt.com/share/68befc00-d494-8013-8967-cee0a840993c	√
5	https://chatgpt.com/share/68befce0-3c68-8013-a2ab-b5ba676ee663	√
6	https://chatgpt.com/share/68befd5f-9b98-8013-b745-29177088bc52	√
7	https://chatgpt.com/share/68befe03-d6b8-8013-8185-e85673dbdb83	√
8	https://chatgpt.com/share/68befeac-0de0-8013-a547-b79793f27135	√
9	https://chatgpt.com/share/68beff6b-3960-8013-8906-a73e94d8a79b	√
10	https://chatgpt.com/share/68bf000c-fab4-8013-ab8e-54fc57199a42	√

Supplementary Table 47: Pipeline tool replacement testing (geNomad replacing checkV) For ChatGPT-5, the status sign means correctness without providing document.

geNomad	Google Gemini 2.5 Flash	Status	Google Gemini 2.5 Pro	Status
1	https://g.co/gemini/share/acea6ad1eb37	×	https://g.co/gemini/share/65847536a056	√
2	https://g.co/gemini/share/df29f5b7c429	×	https://g.co/gemini/share/e7818c8da5cc	√
3	https://g.co/gemini/share/b686decf3435	×	https://g.co/gemini/share/9ee80e8cfd62	√
4	https://g.co/gemini/share/307a5b61f673	×	https://g.co/gemini/share/2ddf2c403bb9	√
5	https://g.co/gemini/share/a04f5932fe8b	×	https://g.co/gemini/share/294d4c9ee372	√
6	https://g.co/gemini/share/f2e288f546ce	×	https://g.co/gemini/share/42b23b0586df	√
7	https://g.co/gemini/share/59720984837f	×	https://g.co/gemini/share/f17b3fcb2218	√
8	https://g.co/gemini/share/b8fc310c7a62	×	https://g.co/gemini/share/5d2395dda1c0	√
9	https://g.co/gemini/share/f38aa9734d74	×	https://g.co/gemini/share/237032c6ad7b	√
10	https://g.co/gemini/share/9d73f44ccccf	√	https://g.co/gemini/share/747facfca93a	√

Supplementary Table 48: Pipeline tool replacement testing (VirSorter2 replacing checkV) For Google Gemini 2.5 Flash and 2.5 Pro, the status sign means correctness without providing document.

VirSorter 2	Google Gemini 2.5 Flash	Status	Google Gemini 2.5 Pro	Status

1	https://g.co/gemini/share/a83db481dad6	×	https://g.co/gemini/share/3e4b6270a1e8	√
2	https://g.co/gemini/share/57258fd400ff	×	https://g.co/gemini/share/61c3f890afbb	√
3	https://g.co/gemini/share/742920ded0cd	√	https://g.co/gemini/share/aaba2c889d7f	√
4	https://g.co/gemini/share/ae198eafc20a	×	https://g.co/gemini/share/bb066d1b04fa	√
5	https://g.co/gemini/share/dcdc87b998fd	√	https://g.co/gemini/share/c4d6e8c69361	√
6	https://g.co/gemini/share/71d18590502c	×	https://g.co/gemini/share/9dd065f58564	√
7	https://g.co/gemini/share/07425703baa6	×	https://g.co/gemini/share/50b4ee3d3dfb	√
8	https://g.co/gemini/share/9a366844dcc9	√	https://g.co/gemini/share/f5ed47ef7992	√
9	https://g.co/gemini/share/4662f569bd76	√	https://g.co/gemini/share/9229305c9954	√
10	https://g.co/gemini/share/91a8e6035755	×	https://g.co/gemini/share/35152f86caeb	√
