# Supplementary material for: Prompt-based bioinformatic pipeline generation for a multi-step metaviral workflow
Source: Bioinform Adv. 2025 Nov 27;6(1):vbaf308. doi: 10.1093/bioadv/vbaf308 (PMC12782108; doi:10.1093/bioadv/vbaf308)

Supplementary Figure 1. Reference codes for metaviral pipeline

Task 1

#input files need to be prepared before running
(1) paired end reads: hiseq_reads_R1.fastq hiseq_reads_R2.fastq (2) adapters.fa (3) Ref_database folder with reference genome fasta files

```
#load all required modules
module load trimmomatic/0.39
module load spades/3.15
module load quast/5.0
module load kraken2
module load checkv/0.8
```

Task 2

```
#use two threads, trim the reads
trimmomatic PE -threads 2 hiseq_reads_R1.fastq hiseq_reads_R2.fastq \
        read1trimmed.fastq read1untrimmed.fastq \
        read2trimmed.fastq read2untrimmed.fastq \
        SLIDINGWINDOW:4:20 MINLEN:25 ILLUMINACLIP:adapters.fa:2:40:15
```

Task 3

```
#assembly (every new run needs to delete the folder)
spades.py --metaviral --pe1-1 read1trimmed.fastq --pe1-2 read2trimmed.fastq -t 2 -o ./spadeout
```

Task 4

```
#check quality of assembly
quast.py ./spadeout/contigs.fasta -1 read1trimmed.fastq -2 read2trimmed.fastq -o ./quastout
```

Task 5

```
#check completeness of the assembly result
checkv end_to_end ./spadeout/contigs.fasta ./checkvout
```

Task 6

```
#build a customized database for Kraken2
kraken2-build --download-taxonomy --db MYDB

for filename in ./Ref_database/*.fa; do
   kraken2-build --add-to-library $filename --db MYDB
done

kraken2-build --build --db MYDB

#annotate contigs by Kraken2
kraken2 --db MYDB --use-names --threads 2 --output kraken2.output --report kraken2.report ./spadeout/contigs.fasta

#annoate from filtered reads
kraken2 --db MYDB --use-names --threads 2 --output kraken2.reads.output --report kraken2.reads.report —-paired
read1trimmed.fastq read2trimmed.fastq
```

Supplementary Figure 2. Prompts used in this paper

Natural language version for pipeline

Act as an experienced bioinformatician proficient in viral genomic assembly and annotation task. I have 3 input files, (1) hiseq_reads_R1.fastq, hiseq_reads_R2.fastq (2) adapters.fa (3) Ref_database folder with reference genome fa files. Trim the files and report both trimmed and unpaired files. After trim, I want to use the trim files to do metaviralSPAdes, which is a specific mode of SPAdes for metaviral assembly with exactly "spade.py --metaviral" option in command. After assembly check the contig quality using trim files. Also check completeness of the assembly result with end to end options. Then, builds a custom database from reference sequences, then annotates contigs and filtered read pairs using the database. Make sure downloads taxonomy data and loop for every reference sequence file in the 'Ref_database' folder add into the database(no threads). Use this database to annotates assembled contigs, and annotates filtered reads(no threads), put results in separate folder. Please load the following modules/tools before using them: trimmomatic/0.39 , spades/3.15 , quast/5.0 , checkv/0.8, kraken2. Use 2 threads and write code in bash.

Structured prompt for pipeline

Act as an experienced bioinformatician proficient in viral genomic assembly and annotation tasks.
Generate code for Task 1 to Task 6 in one block.
Task 1: Module load the tools before running task, use 2 threads except building the Kraken2 custom database and for annotating assembled contigs and filtered reads.
Task 2: Trim the files
Example: trimmomatic PE
Input: hiseq_reads_R1.fastq, hiseq_reads_R2.fastq, adapters.fa
Output: read1trimmed.fastq, read2trimmed.fastq, read1untrimmed.fastq, read2untrimmed.fastq
Tools: trimmomatic/0.39
Coding language: bash
Task 3: Use SPAdes (St. Petersburg genome assembler) program using the 'spades.py' script with the "--metaviral" option for assembling viral metagenomic sequences.
Example: spades.py --metaviral --pe1-1 read1trimmed.fastq --pe1-2 read2trimmed.fastq -t 2 -o ./spadeout
Input: read1trimmed.fastq, read2trimmed.fastq
Output: spadeout folder
Tools: spades/3.15.
Coding language: bash
Task 4: Check quality of assembly
Example: quast.py ./spadeout/contigs.fasta -1 read1trimmed.fastq -2 read2trimmed.fastq -o ./quastout
Input: contigs.fasta from task3 output, read1trimmed.fastq, read2trimmed.fastq
Output: quastout folder
Tools: quast/5.0
Coding language: bash
Task 5: Check completeness of the assembly result, use end to end option, removing "-o" before output folder
Example: checkv end_to_end fasta_file output_folder
Input: contigs.fasta from task3 output
Output: checkvout folder
Tools: checkv/0.8
Coding language: bash
Task 6: Create a custom Kraken2 database, download taxonomy information, and add reference sequences from the Ref_database folder (need a for loop), then build the database without using any threads. Finally, use 2 threads annotate both contigs and filtered reads then use option "--report " generate reports for both.
Input: Ref_database folder with. fa files
Output: two folders
Tools: kraken2
Coding language: bash

Prompt for single tool (without document)

As a computational virologist or bioinformatic expert, please write down a command line for the following task. We want to use a tool called "geNomad" to evaluate the quality of generated contigs. The contig data is in "contigs.fasta", and the pre-downloaded database is in "genomad_db". The output folder is "genomad_output". Please generate this command line.

Prompt for single tool (with document)

As a computational virologist or bioinformatic expert, please write down a command line for the following task. We want to use a tool called "geNomad" to evaluate the quality of generated contigs. The geNomad command line information is described in the given document. The contig data is in "contigs.fasta", and the predownloaded database is in "genomad_db". The output folder is "genomad_output". Please generate this command line.

Prompt for tool replacement in pipeline (without document)

Replace checkv with a new tool genomad to test the quality of the virus genome. The existing pipeline is as follows: “
XXXXXXXXX (reference code)”

Prompt for tool replacement in pipeline (with document)

Replace checkv with a new tool genomad to test the quality of the virus genome. The geNomad command line information is described in the given document. The existing pipeline is as follows: “
XXXXXXXXX (reference code)”

A

```
Assembly                    contigs
# contigs (>= 0 bp)         10
# contigs (>= 1000 bp)      10
# contigs (>= 5000 bp)      10
# contigs (>= 10000 bp)     10
# contigs (>= 25000 bp)     10
# contigs (>= 50000 bp)     5
Total length (>= 0 bp)      597694
Total length (>= 1000 bp)   597694
Total length (>= 5000 bp)   597694
Total length (>= 10000 bp)  597694
Total length (>= 25000 bp)  597694
Total length (>= 50000 bp)  404056
# contigs                   10
Largest contig              129396
Total length                597694
GC (%)                      36.60
N50                         71442
N75                         44628
L50                         4
L75                         6
# N's per 100 kbp           0.00
```

B

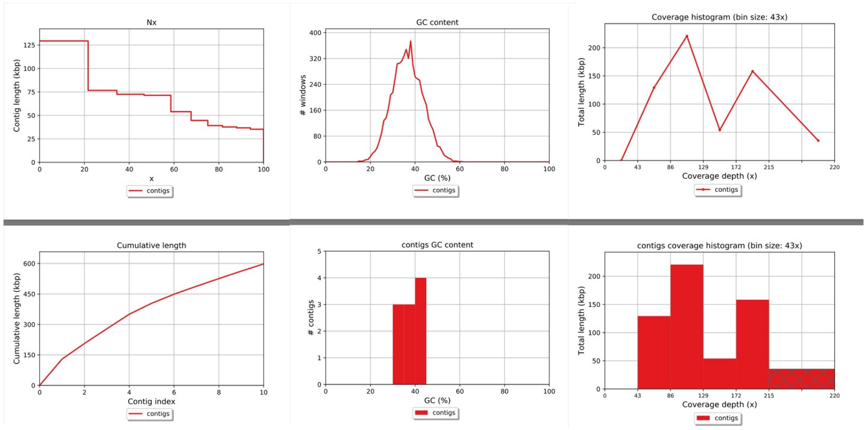

C

```
contig_id       contig_length   provirus        proviral_length gene_count      viral_genes     host_genes      checkv_quality  miuvig_quality  completeness    completeness_method     contamination   kmer_freq       warnings
NODE_1_length_76675_cov_101.121648_cutoff_0_type_circular       76675   No      NA      104     17      0       Medium-quality  Genome-fragment 71.64   HMM-based (lower-bound) 0.0     1.0     low-confidence DTR
NODE_2_length_44628_cov_173.901506_cutoff_0_type_circular       44628   No      NA      57      31      0       Medium-quality  Genome-fragment 73.35   HMM-based (lower-bound) 0.0     1.0     low-confidence DTR
NODE_1_length_129396_cov_59.937728_cutoff_5_type_linears        129396  No      NA      230     69      0       Medium-quality  Genome-fragment 84.78   HMM-based (lower-bound) 0.0     1.0
NODE_2_length_72533_cov_106.941385_cutoff_5_type_linears        72533   No      NA      90      22      0       Medium-quality  Genome-fragment 58.04   HMM-based (lower-bound) 0.0     1.0
NODE_3_length_71442_cov_108.617025_cutoff_5_type_linears        71442   No      NA      112     24      0       Medium-quality  Genome-fragment 65.61   HMM-based (lower-bound) 0.0     1.0
NODE_4_length_54010_cov_143.602637_cutoff_5_type_linears        54010   No      NA      73      28      0       High-quality    High-quality    94.26   HMM-based (lower-bound) 0.0     1.01
NODE_5_length_39188_cov_198.015162_cutoff_5_type_linears        39188   No      NA      63      25      0       Medium-quality  Genome-fragment 56.95   HMM-based (lower-bound) 0.0     1.0
NODE_6_length_37727_cov_205.817477_cutoff_5_type_linears        37727   No      NA      60      24      0       Medium-quality  Genome-fragment 50.83   HMM-based (lower-bound) 0.0     1.0
NODE_7_length_36768_cov_211.427789_cutoff_5_type_linears        36768   No      NA      65      23      0       Medium-quality  Genome-fragment 52.02   HMM-based (lower-bound) 0.0     1.0
NODE_8_length_35327_cov_219.687858_cutoff_5_type_linears        35327   No      NA      51      21      0       Medium-quality  Genome-fragment 79.14   HMM-based (lower-bound) 0.0     1.0
```

D

```
  0.01  129     129     U       0       unclassified
 99.99  985268  0       R       1       root
 99.99  985268  0       D       10239     Viruses
 41.67  410628  0       D1      12333       unclassified bacterial viruses
  8.34  82204   82204   S       1348402       Pseudoalteromonas phage PSA-HS9
  8.33  82126   82126   S       1348397       Pseudoalteromonas phage PSA-HP1
  8.33  82119   82119   S       1348399       Pseudoalteromonas phage PSA-HS2
  8.33  82118   82118   S       1348400       Pseudoalteromonas phage PSA-HM1
  8.33  82061   82061   S       1348398       Pseudoalteromonas phage PSA-HS6
 41.65  410413  0       D1      2731341     Duplodnaviria
 41.65  410413  0       K       2731360       Heunggongvirae
 41.65  410413  0       P       2731618         Uroviricota
 41.65  410413  0       C       2731619           Caudoviricetes
  8.34  82141   0       F       2946166             Pachyviridae
  8.34  82141   0       G       2946816               Baltivirus
  8.34  82141   0       S       2955411                 Baltivirus phi18tres
  8.34  82141   82141   S1      1327983                   Cellulophaga phage phi18:3
  8.33  82122   0       G       1918017             Helsingorvirus
  8.33  82122   0       S       1918192               Helsingorvirus Cba181
  8.33  82122   82122   S1      1327982                 Cellulophaga phage phi18:1
  8.33  82117   0       G       1918718             Cbastvirus
  8.33  82117   0       S       1918720               Cbastvirus ST
  8.33  82117   0       S1      756282                  Cellulophaga phage phiST
  8.33  82117   82117   S2      1327992                   Cellulophaga phage phi13:1
  8.33  82085   0       F       2946168             Pervagoviridae
  8.33  82085   0       G       2948648               Callevirus
  8.33  82085   0       S       2955570                 Callevirus phi38una
  8.33  82085   82085   S1      1327977                   Cellulophaga phage phi38:1
  8.32  81948   0       C1      2788787             unclassified Caudoviricetes
  8.32  81948   81948   S       1327999               Cellulophaga phage phi38:2
 16.67  164227  0       D1      2731342     Monodnaviria
 16.67  164227  0       K       2732091       Sangervirae
 16.67  164227  0       P       2732412         Phixviricota
 16.67  164227  0       C       2732413           Malgrandaviricetes
 16.67  164227  0       O       2732414             Petitvirales
 16.67  164227  0       F       10841                 Microviridae
 16.67  164227  0       F1      1910950                 Bullavirinae
  8.33  82114   0       G       1910954                   Sinsheimervirus
  8.33  82114   0       S       10847                       Sinsheimervirus phiX174
  8.33  82114   82114   S1      2886930                       Escherichia phage phiX174
  8.33  82113   0       G       1910951                   Alphatrevirus
  8.33  82113   0       S       1945589                     Alphatrevirus alpha3
  8.33  82113   82113   S1      10849                         Escherichia phage alpha3
```

E

```
100.00  10      0       R       1       root
100.00  10      0       D       10239     Viruses
 50.00  5       0       D1      12333       unclassified bacterial viruses
 10.00  1       1       S       1348397       Pseudoalteromonas phage PSA-HP1
 10.00  1       1       S       1348398       Pseudoalteromonas phage PSA-HS6
 10.00  1       1       S       1348399       Pseudoalteromonas phage PSA-HS2
 10.00  1       1       S       1348400       Pseudoalteromonas phage PSA-HM1
 10.00  1       1       S       1348402       Pseudoalteromonas phage PSA-HS9
 50.00  5       0       D1      2731341     Duplodnaviria
 50.00  5       0       K       2731360       Heunggongvirae
 50.00  5       0       P       2731618         Uroviricota
 50.00  5       0       C       2731619           Caudoviricetes
 10.00  1       0       G       1918017             Helsingorvirus
 10.00  1       0       S       1918192               Helsingorvirus Cba181
 10.00  1       1       S1      1327982                 Cellulophaga phage phi18:1
 10.00  1       0       G       1918718             Cbastvirus
 10.00  1       0       S       1918720               Cbastvirus ST
 10.00  1       0       S1      756282                  Cellulophaga phage phiST
 10.00  1       1       S2      1327992                   Cellulophaga phage phi13:1
 10.00  1       0       C1      2788787             unclassified Caudoviricetes
 10.00  1       1       S       1327999               Cellulophaga phage phi38:2
 10.00  1       0       F       2946166             Pachyviridae
 10.00  1       0       G       2946816               Baltivirus
 10.00  1       0       S       2955411                 Baltivirus phi18tres
 10.00  1       1       S1      1327983                   Cellulophaga phage phi18:3
 10.00  1       0       F       2946168             Pervagoviridae
 10.00  1       0       G       2948648               Callevirus
 10.00  1       0       S       2955570                 Callevirus phi38una
 10.00  1       1       S1      1327977                   Cellulophaga phage phi38:1
```

Supplementary Figure 3. Expected outcomes of metaviral pipeline from assembly, quality of assembly, completeness of quality and annotate contigs. (A) Assembly results report with detail found contigs information using tool Quast. All statistics are based on contigs of size >= x bp, unless otherwise noted (e.g., "# contigs (>= 0 bp)" and "Total length (>= 0 bp)" include all contigs). All contigs are larger than 25,000 base pairs (bp). The total length of the assembly is 597,694 bp. The largest contig is 129,396 bp long. The assembly has a GC content of 36.60%. (B) Assembly visualization plots show the quality of assembly. (C) Completeness of the assembly output provides a comprehensive quality assessment of metagenomic viral genomes. It includes metrics like contig id, contig length, total genes, viral and host genes. In our sample data, there are 10 contigs founded in provide sample data, 9 contigs with medium quality and 1 contig with high quality. (D)The Kraken2 read report reflects the classification of individual raw read sequences. It shows that 99.99% of the sequences were classified as viruses, with a significant proportion belonging to unclassified bacterial viruses, Duplodnaviria, and Monodnaviria. The twelve identified viral species share the equal amount of relative abundance (8.32%) as expected. (E) Classification taxonomic report shows the classification of ten contig sequences, all classified as viruses, with half being unclassified bacterial viruses and the other half belonging to the Duplodnaviria group. Different species within the Pseudoalteromonas phage and Cellulophaga phage were identified. These reports together provide a comprehensive picture of the viral diversity and abundance in the sample data provided.

Supplementary Figure 4. Common errors in pipeline building from ChatGPT models

OpenAI ChatGPT 3.5 | OpenAI ChatGPT4.0 | ChatGPT-4o

Task 1

ChatGPT 3.5:

```
# Set up variables
input_dir="input_files/"
output_dir="output/"
trimmomatic_path="trimmomatic/0.39"
spades_path="spades/3.15"
quast_path="quast/5.0"
checkv_path="checkv/0.8"
kraken2_path="kraken2"
```

ChatGPT-4o:

```
module load trimmomatic/0.39 spades/3.15 quast/5.0 checkv/0.8 kraken2/2.0
```

Task 2

ChatGPT 3.5:

```
# Trim reads using Trimmomatic
java -jar trimmomatic-0.39.jar PE -threads 2 \
    $reads_R1 $reads_R2 \
    trimmed_R1.fastq unpaired_R1.fastq \
    trimmed_R2.fastq unpaired_R2.fastq \
    ILLUMINACLIP:$adapters:2:30:10 \
    LEADING:3 TRAILING:3 SLIDINGWINDOW:4:15 MINLEN:36
```

ChatGPT4.0:

```
# Trimming reads
echo "Trimming reads..."
trimmomatic PE -threads 2 $R1 $R2 \
            trimmed_R1.paired.fastq trimmed_R1.unpaired.fastq \
            trimmed_R2.paired.fastq trimmed_R2.unpaired.fastq \
            ILLUMINACLIP:$ADAPTERS:2:30:10 MINLEN:36
```

ChatGPT-4o:

```
# Task 2: Trim the files
trimmomatic PE -threads 2 hiseq_reads_R1.fastq hiseq_reads_R2.fastq read1trimmed

ed.fastq read2trimmed.fastq read2untrimmed.fastq ILLUMINACLIP:adapters.fa:2:30:10
```

Task 3

ChatGPT4.0:

```
spades.py --metaviral \
  -1 hiseq_reads_R1_paired.fastq \
  -2 hiseq_reads_R2_paired.fastq \
  --only-assembler \
  -t 2 \
  -o metaviral_spades_output
```

ChatGPT-4o:

```
echo "Running metaviralSPAdes..."
ASSEMBLY_DIR="${OUTPUT_DIR}/spades_output"
spades.py --meta -1 $TRIM_R1 -2 $TRIM_R2 -o $ASSEMBLY_DIR --threads 2
```

Task 4

ChatGPT 3.5:

```
quast.py metaviral_assembly/contigs.fasta -o assembly_quality
```

ChatGPT4.0:

```
# Quality check using QUAST
echo "Checking quality of assembled contigs..."
quast.py -o quast_output assembly_output/contigs.fasta
```

ChatGPT-4o:

```
quast.py -o $QUALITY_DIR $ASSEMBLY_DIR/contigs.fasta
```

Task 5

ChatGPT 3.5:

```
# Check assembly completeness using CheckV
checkv end_to_end metaviral_spades_output/contigs.fasta -t 2 -d checkv_output
```

Task 6

ChatGPT 3.5:

```
mkdir -p custom_database

for ref_file in $ref_db/*.fa; do
    name=$(basename $ref_file .fa)
    kraken2-build --add-to-library $ref_file --db custom_database --name $name
done
```

ChatGPT-4o:

```
kraken2 --db $KRAKEN2_DB --paired --output $KRAKEN2_OUTPUT/reads_annotation.txt
```

Supplementary Figure 5. Common errors in pipeline building from ChatGPT models

Columns: OpenAI o1 | OpenAI o1-mini | GPT-4.1

Task 1

Task 2

Task 3

OpenAI o1:

```
mkdir -p spades_output
spades.py --metaviral \
  -1 trimmed_R1_paired.fastq \
  -2 trimmed_R2_paired.fastq \
  -s trimmed_R1_unpaired.fastq \
  -s2 trimmed_R2_unpaired.fastq \
  -o spades_output \
  -t 2
```

Task 4

OpenAI o1:

```
quast.py -t $THREADS \
  -o quast_output \
  spades_metaviral_output/contigs.fasta \
  -1 hiseq_reads_R1_paired_trimmed.fastq \
  -2 hiseq_reads_R2_paired_trimmed.fastq \
  -s hiseq_reads_R1_unpaired_trimmed.fastq,hiseq_reads_R2_unpaired_trimmed.fastq
```

OpenAI o1-mini:

```
quast.py ${ASSEMBLY_DIR}/contigs.fasta \
    -R ${REF_DB_DIR}/*.fa \
    -o ${ASSEMBLY_QUALITY_DIR} \
    -t ${THREADS}
```

Task 5

OpenAI o1-mini:

```
checkv end_to_end \
  --assembly ${ASSEMBLY_DIR}/contigs.fasta \
  --db ${DATABASE_DIR} \
  --out-dir ${CHECKV_DIR} \
  --threads ${THREADS}
```

Task 6

OpenAI o1:

```
# Combine trimmed and unpaired reads
cat trimmed_R1_paired.fastq trimmed_R1_unpaired.fastq > combined_trimmed_R1.fastq
cat trimmed_R2_paired.fastq trimmed_R2_unpaired.fastq > combined_trimmed_R2.fastq

# Run Kraken2 on the combined reads
kraken2 --db $kraken2_db_dir \
  --output kraken2_reads_output.txt \
  --report kraken2_reads_report.txt \
  --paired combined_trimmed_R1.fastq combined_trimmed_R2.fastq
```

OpenAI o1-mini:

```
kraken2 --db ${DATABASE_DIR} \
  --threads ${THREADS} \
  --output ${ANNOTATION_DIR}/reads_kraken2.out \
  --report ${ANNOTATION_DIR}/reads_kraken2.report \
  ${TRIMMED_DIR}/combined_trimmed.fastq
```

Supplementary Figure 6. Common errors in pipeline building from ChatGPT models

Columns: 4.1 mini | GPT-4.5 | GPT-5

Task 1

Task 2

Task 3

GPT-5:

```
"${SPADES_BIN}" --metaviral \
  -1 "${TRIM_DIR}/R1.paired.fastq.gz" \
  -2 "${TRIM_DIR}/R2.paired.fastq.gz" \
  -s "${TRIM_DIR}/singletons.fastq.gz" \
  -t "${THREADS}" \
  -o "${SPADES_DIR}"
```

Task 4

GPT-5:

```
quast.py "${ASSEMBLED}" \
  --pe1 "${TRIM_DIR}/R1.paired.fastq.gz" \
  --pe2 "${TRIM_DIR}/R2.paired.fastq.gz" \
  --single "${TRIM_DIR}/singletons.fastq.gz" \
  -o "${QUAST_DIR}" -t "${THREADS}"
```

Task 5

4.1 mini:

```
checkv end_to_end \
  $OUTPUT_DIR/spades_output/contigs.fasta \
  $OUTPUT_DIR/checkv_output \
  -t $THREADS \
  --all
```

Task 6

4.1 mini:

```
# Annotate filtered paired reads concatenated
cat ${TRIMMED_DIR}/trimmed_R1_paired.fastq ${TRIMMED_DIR}/trimmed_R2_paired.fastq >

kraken2 --db $KRAKEN_DB_DIR \
  --output ${ANNOTATION_DIR}/reads_kraken2_output.txt \
  --report ${ANNOTATION_DIR}/reads_kraken2_report.txt \
  ${TRIMMED_DIR}/filtered_paired_combined.fastq
```

GPT-4.5:

```
kraken2 --db kraken2_custom_db --threads 2 \
  combined_filtered_reads.fastq \
  --output kraken_reads_output.txt \
  --report kraken_reads_report.txt
```

GPT-5:

```
kraken2 \
  --db "$KRAKEN_DB_DIR" \
  --fasta-input \
  --use-names \
  --report "$ANNOT_CONTIGS_DIR/contigs.kraken2.report" \
  --output "$ANNOT_CONTIGS_DIR/contigs.kraken2.output" \
  "$CONTIGS"
```

Supplementary Figure 7. Common errors in pipeline building from Claude models and other LLMs

Claude 3.5 Sonnet | Claude 3.7 Sonnet | Claude Sonnet 4 | Claude Opus 4

Task 1

Claude 3.5 Sonnet:

```
# Task 1: Load necessary modules
echo "Loading required modules..."
module load trimmomatic/0.39
module load spades/3.15.5
module load quast/5.0
module load checkv/0.8
module load kraken2/2.1.2
```

Claude 3.7 Sonnet:

```
# Task 1: Load necessary modules
echo "Loading required modules..."
module load trimmomatic/0.39
module load spades/3.15.5
module load quast/5.0
module load checkv/0.8
module load kraken2/2.1.2
```

Task 2

Claude Sonnet 4:

```
trimmomatic PE -threads $THREADS \
    -phred33 \
    $INPUT_R1 $INPUT_R2 \
    trimmed_reads/trimmed_R1_paired.fastq trimmed_reads/trimmed_R1_unpaired.fastq \
    trimmed_reads/trimmed_R2_paired.fastq trimmed_reads/trimmed_R2_unpaired.fastq \
    ILLUMINACLIP:$ADAPTERS:2:30:10 \
    LEADING:3 \
    TRAILING:3 \
    SLIDINGWINDOW:4:15 \
    MINLEN:36 \
    2>&1 | tee logs/trimmomatic.log
```

Task 3

Claude 3.7 Sonnet:

```
# Step 2: Metaviral assembly with SPAdes
echo "Step 2: Running metaviralSPAdes assembly..."
spades.py --metaviral \
    -1 trimmed_reads/R1_paired.fastq \
    -2 trimmed_reads/R2_paired.fastq \
    -s trimmed_reads/R1_unpaired.fastq \
    -s trimmed_reads/R2_unpaired.fastq \
    -o assembly_output \
    -t $THREADS
```

Claude Sonnet 4:

```
# Step 2: Metaviral assembly with SPAdes
echo "Step 2: Running metaviralSPAdes assembly..."
spades.py --metaviral \
    -1 trimmed_reads/R1_paired.fastq \
    -2 trimmed_reads/R2_paired.fastq \
    -s trimmed_reads/R1_unpaired.fastq \
    -s trimmed_reads/R2_unpaired.fastq \
    -o assembly_output \
    -t $THREADS
```

Claude Opus 4:

```
# Step 2: Metaviral assembly with SPAdes
echo "Step 2: Running metaviralSPAdes assembly..."
spades.py --metaviral \
    -1 trimmed_reads/R1_paired.fastq \
    -2 trimmed_reads/R2_paired.fastq \
    -s trimmed_reads/R1_unpaired.fastq \
    -s trimmed_reads/R2_unpaired.fastq \
    -o assembly_output \
    -t $THREADS
```

Task 4

Claude 3.5 Sonnet:

```
quast.py spades_output/contigs.fasta \
    -r trimmed_R1_paired.fastq,trimmed_R2_paired.fastq \
    -o quast_output -t $THREADS
```

Claude 3.7 Sonnet:

```
# Step 3: Quality assessment with QUAST
echo "Step 3: Assessing assembly quality with QUAST..."
quast.py \
    assembly/contigs.fasta \
    -r trimmed_reads/trimmed_R1_paired.fastq \
    trimmed_reads/trimmed_R2_paired.fastq \
    -o quality_assessment \
    --threads $THREADS
```

Claude Sonnet 4:

```
# Step 4: End-to-end completeness check with QUAST
echo "Step 4: Checking assembly completeness (end-to-end mapping)..."
quast.py assembly_output/contigs.fasta \
    -1 trimmed_reads/R1_paired.fastq \
    -2 trimmed_reads/R2_paired.fastq \
    --pe1 trimmed_reads/R1_unpaired.fastq \
    --pe2 trimmed_reads/R2_unpaired.fastq \
    -o quality_assessment/quast_completeness \
    -t $THREADS
```

Claude Opus 4:

```
# Step 4: End-to-end completeness check with QUAST
echo "Step 4: Checking assembly completeness (end-to-end mapping)..."
quast.py assembly_output/contigs.fasta \
    -1 trimmed_reads/R1_paired.fastq \
    -2 trimmed_reads/R2_paired.fastq \
    --pe1 trimmed_reads/R1_unpaired.fastq \
    --pe2 trimmed_reads/R2_unpaired.fastq \
    -o quality_assessment/quast_completeness \
    -t $THREADS
```

Task 5

Task 6

Meta Llama 3 | deepseek R1

Task 1

deepseek R1:

```
# Task 1: Load modules with specified threads
module load trimmomatic/0.39 \
            spades/3.15.5 \
            quast/5.0 \
            checkv/0.8 \
            kraken2/2.1.2
```

Task 2

Llama 3:

```
# Trim files
trimmomatic PE -threads 2 hiseq_reads_R1.fastq hiseq_reads_R2.fastq \
  hiseq_reads_R1_paired.fastq hiseq_reads_R1_unpaired.fastq \
  hiseq_reads_R2_paired.fastq hiseq_reads_R2_unpaired.fastq \
  ILLUMINACLIP:adapters.fa:2:30:10
```

deepseek R1:

```
# Task 2: Read trimming
trimmomatic PE -threads 2 \
    hiseq_reads_R1.fastq hiseq_reads_R2.fastq \
    read1trimmed.fastq read1untrimmed.fastq \
    read2trimmed.fastq read2untrimmed.fastq \
    ILLUMINACLIP:adapters.fa:2:30:10
```

Task 3

Llama 3:

```
# Metaviral assembly with SPAdes
spade.py --metaviral -1 trimmed_R1.fastq -2 trimmed_R2.fastq \
  -o metaviral_spades_output -t 2
```

deepseek R1:

```
echo "Running metaSPAdes..."
spades.py --metaviral \
  -1 trimmed_reads/trimmed_1P.fastq \
  -2 trimmed_reads/trimmed_2P.fastq \
  -s trimmed_reads/trimmed_1U.fastq \
  -s trimmed_reads/trimmed_2U.fastq \
  -o spades_output \
  -t $THREADS
```

Task 4

Llama 3:

```
# Check contig quality
quast.py -o quast_output -t 2 metaviral_spades_output/contigs.fasta
```

deepseek R1:

```
echo "Running QUAST..."
quast.py spades_output/contigs.fasta \
  -r Ref_database/*.fa \
  -o quast_results
```

Task 5

Llama 3:

```
# Check completeness using CheckV
checkv end_to_end metaviral_spades_output/contigs.fasta \
  -o checkv_output -t 2
```

Supplementary Figure 8. Common errors in pipeline building from Google Gemini

Gemini | Gemini 2.5 Flash | Gemini 2.5 Pro

Task 1

Gemini 2.5 Flash:

```
module load trimmomatic/0.39
module load spades/3.15.5
module load quast/5.0
module load checkv/0.8
module load kraken2
```

Gemini 2.5 Pro:

```
module load trimmomatic/0.39
module load spades/3.15
module load quast/5.0
module load checkv/0.8
module load kraken2/2.1.2 # Version may vary, using a recent one
```

Task 2

Gemini:

```
# Perform trimming
trim_reads hiseq_reads_R1.fastq hiseq_reads_R2.fastq trimmed
```

Task 3

Gemini:

```
# Perform metaviral assembly using SPAdes
spades.py --metaviral -t 2 -o $assembly_dir \
          trimmed_R1_trimmed.fastq trimmed_R2_trimmed.fastq
```

Task 4

Gemini:

```
quast.py -t 2 -o $qc_dir $assembly_dir/contigs.fasta
```

Gemini 2.5 Flash:

```
quast.py $ASSEMBLY_DIR/contigs.fasta \
    -o $QUAST_DIR \
    -t $THREADS
```

Gemini 2.5 Pro:

```
quast.py $ASSEMBLY_DIR/contigs.fasta \
    -o $QUAST_DIR \
    -t $THREADS
```

Task 5

Gemini:

```
checkv analyze -t 2 -d $qc_dir $assembly_dir/contigs.fasta
```

Gemini 2.5 Flash:

```
checkv end_to_end \
    metaviral_assembly/contigs.fasta \
    trimmed_reads/trimmed_R1.fastq \
    trimmed_reads/trimmed_R2.fastq \
    assembly_qc/checkv_results \
    --threads 2
```

Task 6

Gemini:

```
# Build custom database from reference sequences (no threads)
kraken2-build --db $annotation_dir/refdb --threads 1 \
              $(find $ref_dir -name "*.fa")

# Annotate contigs and filtered reads using Kraken2 (no threads)
for ref in $(find $ref_dir -name "*.fa"); do
  ref_name=$(basename $ref .fa)
  kraken2-annotate --db $annotation_dir/refdb --threads 1 \
                   --output $annotation_dir/$ref_name.contigs.out \
                   $assembly_dir/*.fasta
  kraken2-annotate --db $annotation_dir/refdb --threads 1 \
                   --output $annotation_dir/$ref_name.reads.out \
                   trimmed_R1_trimmed.fastq trimmed_R2_trimmed.fastq
done

echo "Assembly and annotation completed. Results in $assembly_dir and $[illegible]
```

Gemini 2.5 Flash:

```
echo "Starting Kraken2 annotation for filtered reads..."
# Combine trimmed and untrimmed reads for comprehensive analysis
cat read1trimmed.fastq read1untrimmed.fastq read2trimmed.fastq read2untrimmed.fast[illegible]
kraken2 --db ./kraken2_db --threads ${THREADS} all_reads.fastq --report ./kraken2_[illegible]
echo "Filtered reads annotation complete."
```

Supplementary Figure 9. ChatGPT o1-mini and ChatGPT o1-preview. (A) Total correctness by natural language version prompts (B) Total correctness by structured prompts (C) Task-wise correctness by natural language version prompts (D) Task-wise correctness by structured prompts (E) Tool replacement test in existing pipeline (geNomad replacing CheckV and virsorter2 replacing CheckV)

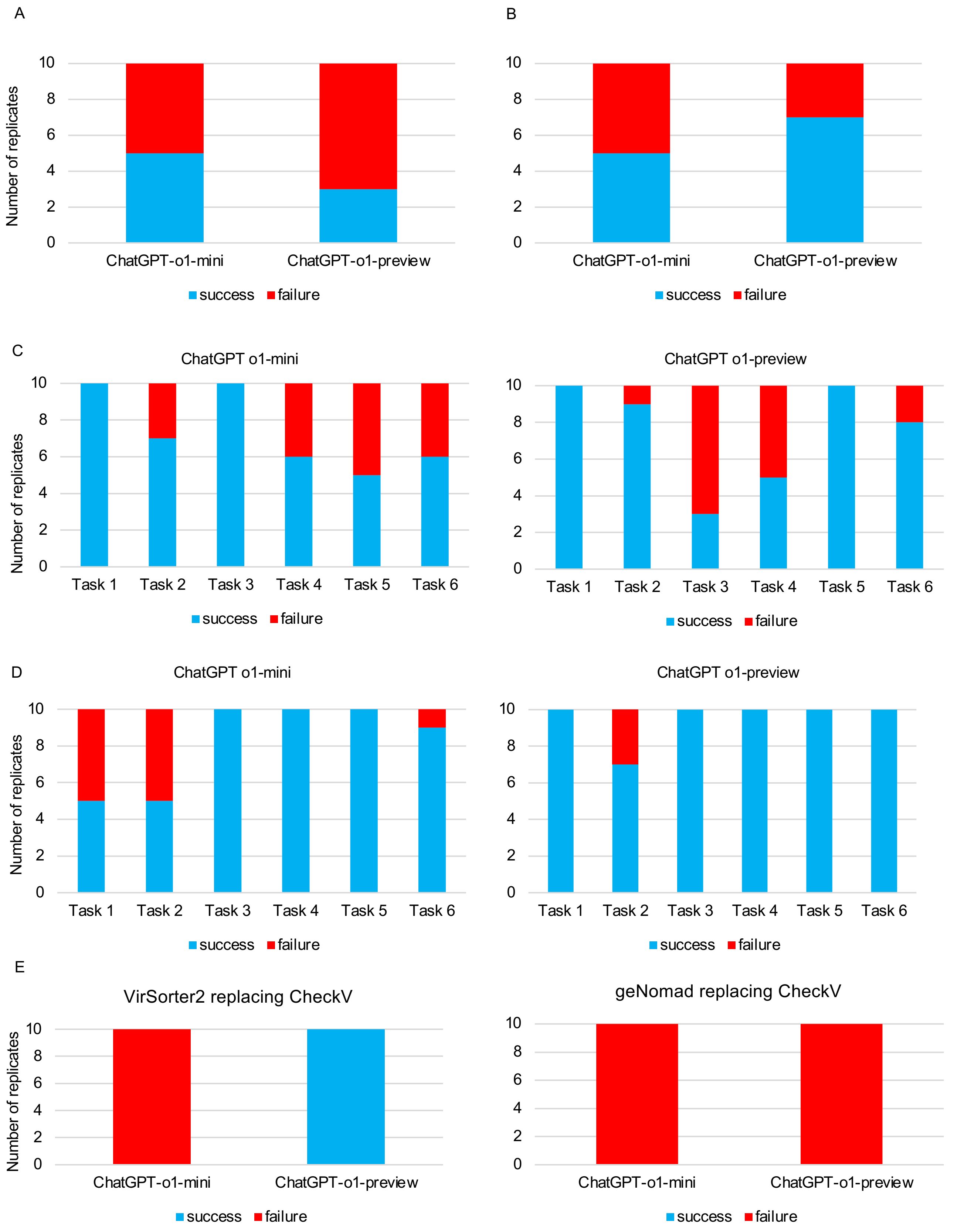

Supplementary Figure 10. ChatGPT 4.1 ChatGPT 4.1-mini ChatGPT 4.5 and ChatGPT 5 (A) Total correctness by natural language version prompts (B) Total correctness by structured prompts (C) Task-wise correctness by natural language version prompts and structured prompts (D) ChatGPT 4.1 and ChatGPT 4.1 mini (E) ChatGPT 4.5 (F) ChatGPT 5 (G) Tool replacement test in existing pipeline (geNomad replacing CheckV and virsorter2 replacing CheckV)

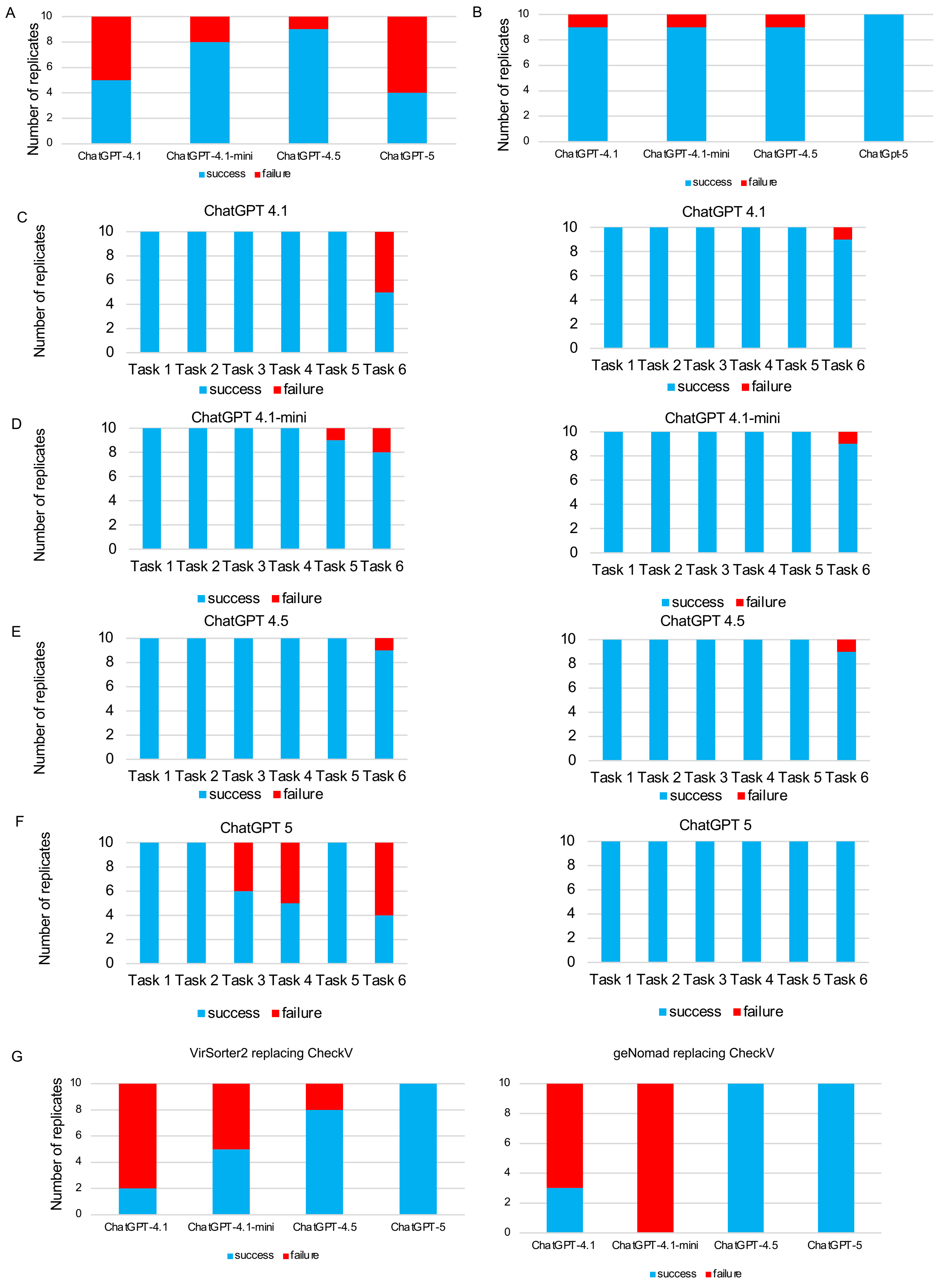

Supplementary Figure 11. Testing results with other Large Language Models (LLMs) (A) Pipeline generation with natural language prompts (B) and structured prompts for their total correctness and task-wise correctness among ten cases. (C) Correctness for six tools among ten testing cases (D) Tool replacement test in existing pipeline (geNomad replacing CheckV and Virsorter2 replacing CheckV)

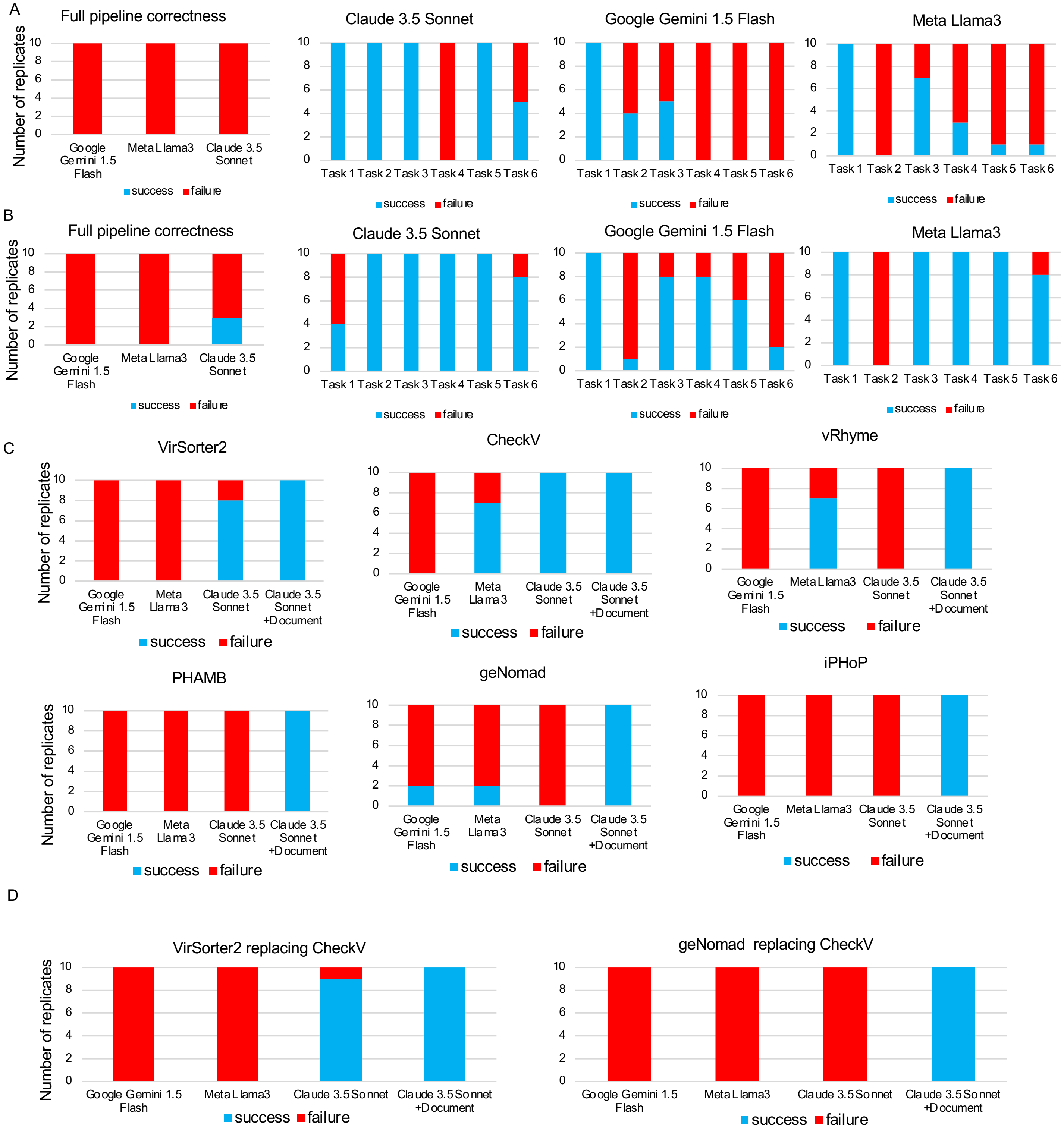

Supplementary Figure 12. Deepseek R1 and Claude 3.7 Sonnet. (A) Total correctness by natural language version prompts (B) Total correctness by structured prompts (C) Task-wise correctness by natural language version prompts (D) Task-wise correctness by structured prompts (E) Tool replacement test in existing pipeline (geNomad replacing CheckV and virsorter2 replacing CheckV)

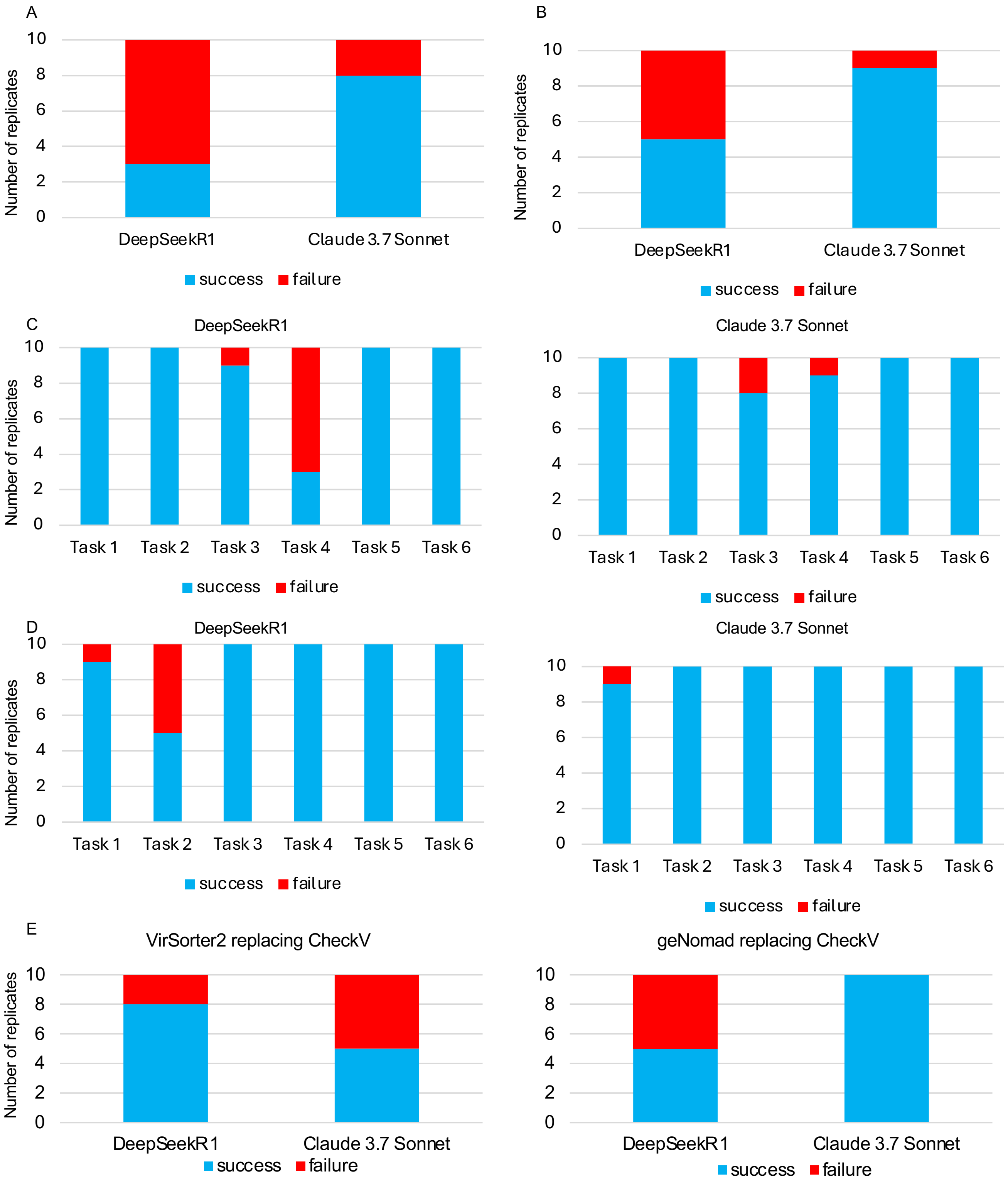

Supplementary Figure 13. Claude Sonnet 4, Claude Opus 4 and Sonnet 4.5 (A) Total correctness by natural language version prompts (B) Total correctness by structured prompts. Task-wise correctness by natural language version prompts and task-wise correctness by structured prompts for (C) Claude Sonnet 4 (D) Claude Opus 4. (E) Tool replacement test in existing pipeline (geNomad replacing CheckV and virsorter2 replacing CheckV)

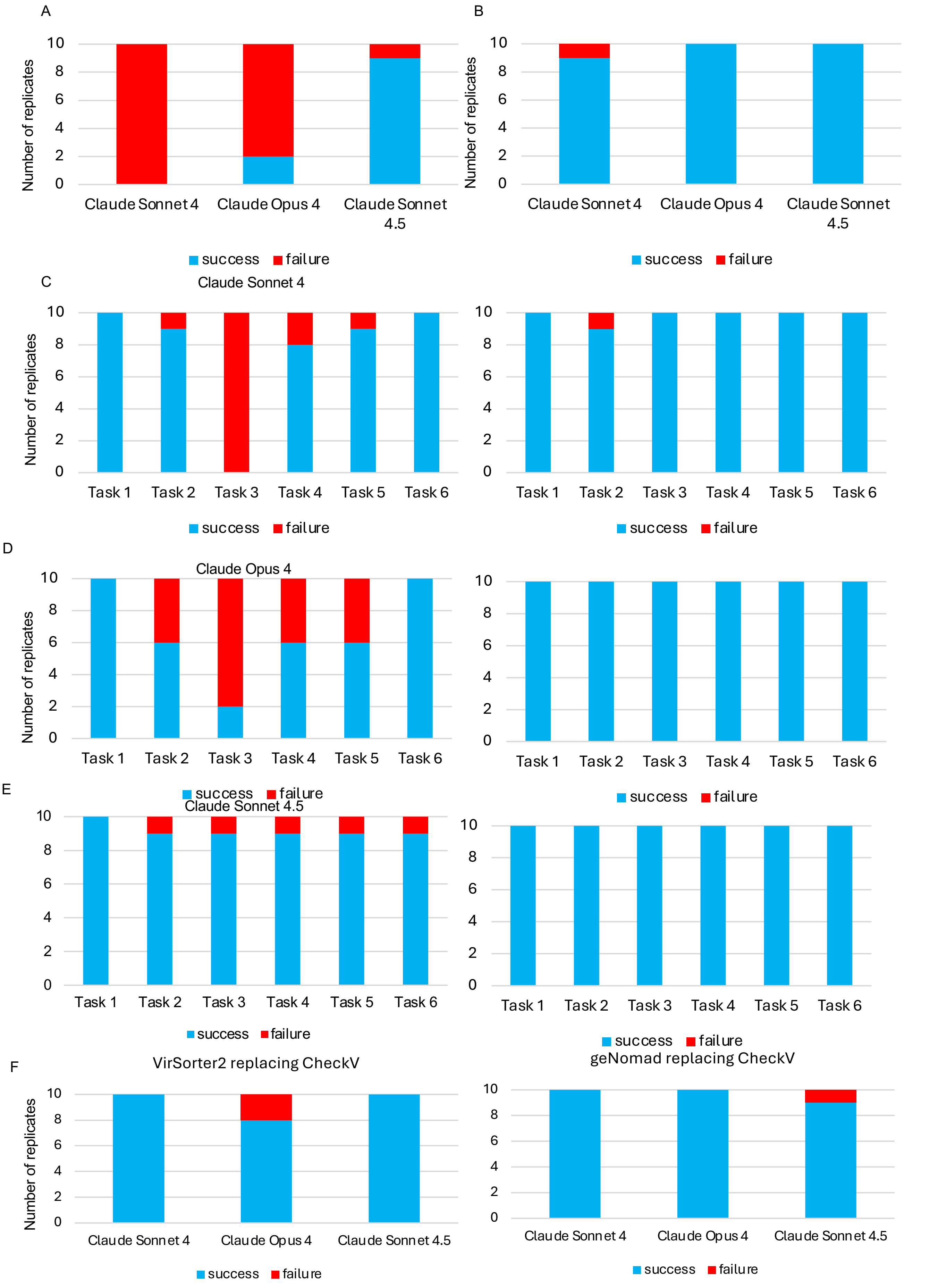

Supplementary Figure 14. Google Gemini 2.5 Flash and Google Gemini 2.5 Pro (A) Total correctness by natural language version prompts (B) Total correctness by structured prompts. Task-wise correctness by natural language version prompts and task-wise correctness by structured prompts for (C) Google Gemini 2.5 Flash (D) Google Gemini 2.5 Pro (E) Tool replacement test in existing pipeline (geNomad replacing CheckV and virsorter2 replacing CheckV)

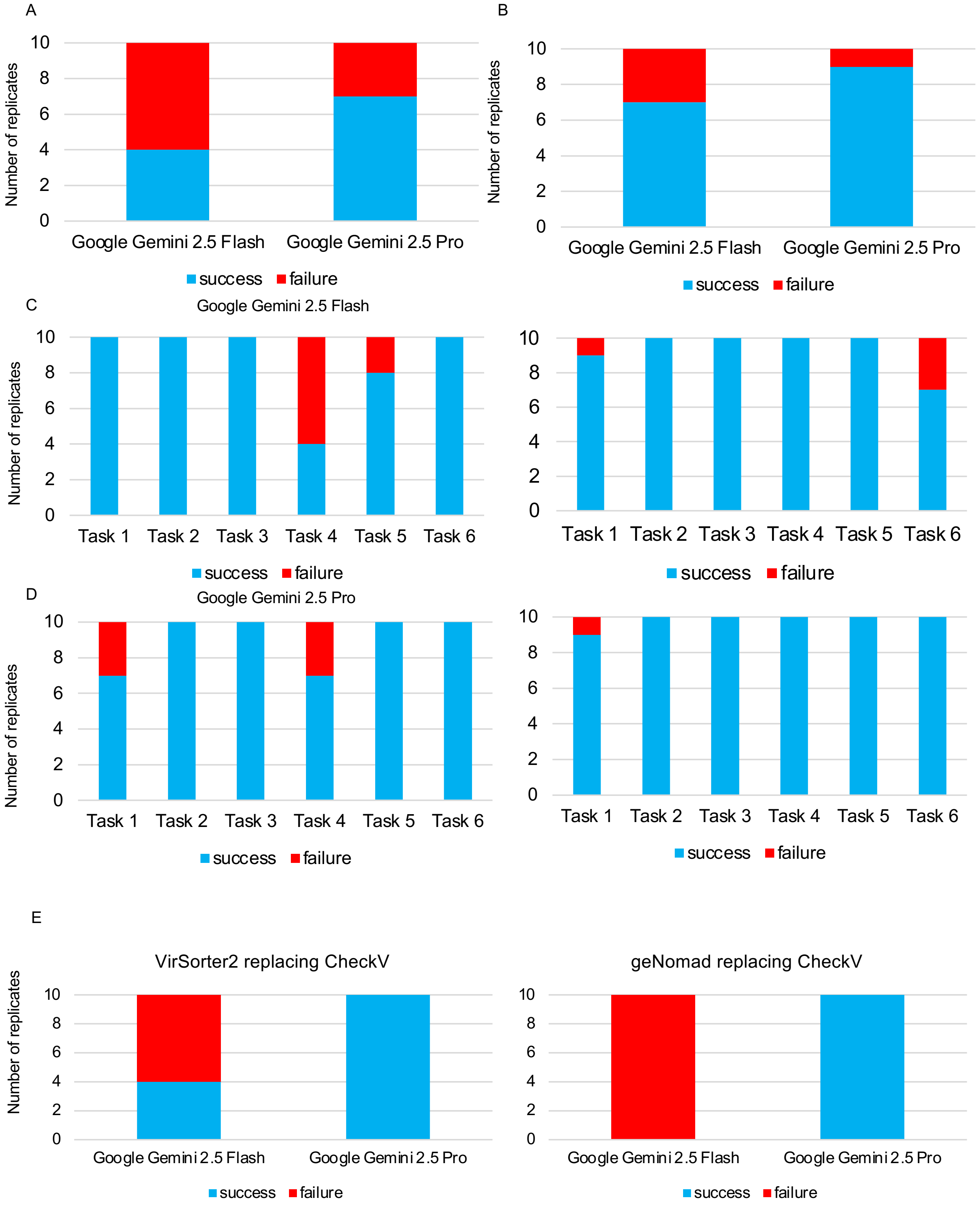

Supplementary Figure 15. P-values from two sample proportion Z test to show the if two methods yield different performance or not (A) Natural language prompt performance (B) Structure prompt performance

A

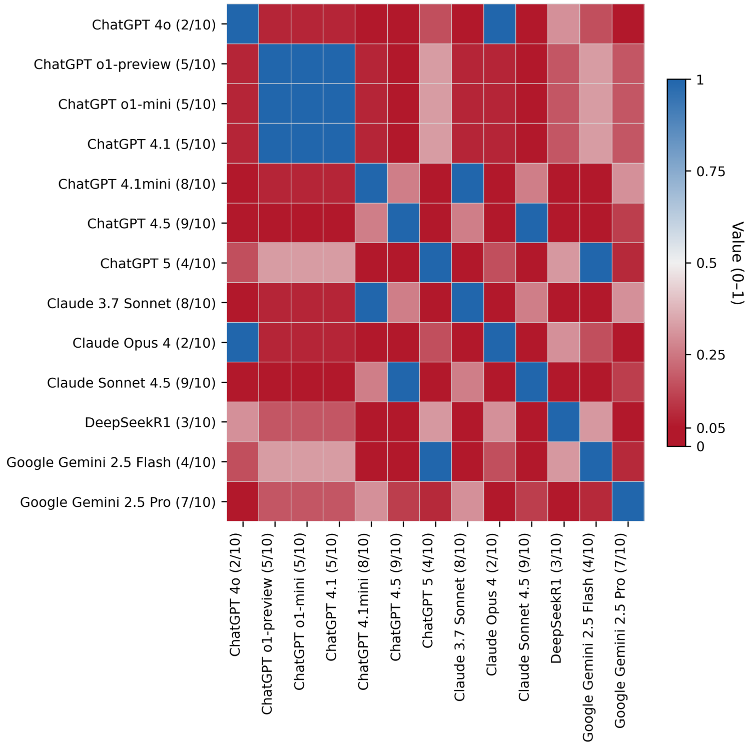

	ChatGPT 4o (2/10)	ChatGPT o1-preview (5/10)	ChatGPT o1-mini (5/10)	ChatGPT 4.1 (5/10)	ChatGPT 4.1mini (8/10)	ChatGPT 4.5 (9/10)	ChatGPT 5 (4/10)	Claude 3.7 Sonnet (8/10)	Claude Opus 4 (2/10)	Claude Sonnet 4.5 (9/10)	DeepSeekR1 (3/10)	Google Gemini 2.5 Flash (4/10)	Google Gemini 2.5 Pro (7/10)
ChatGPT 4o (2/10)	1	0.0797995	0.0797995	0.0797995	0.00364518	0.00082684	0.164557	0.00364518	1	0.00082684	0.302788	0.164557	0.0123094
ChatGPT o1-preview (5/10)	0.0797995	1	1	1	0.0797995	0.025481	0.326548	0.0797995	0.0797995	0.025481	0.180655	0.326548	0.180655
ChatGPT o1-mini (5/10)	0.0797995	1	1	1	0.0797995	0.025481	0.326548	0.0797995	0.0797995	0.025481	0.180655	0.326548	0.180655
ChatGPT 4.1 (5/10)	0.0797995	1	1	1	0.0797995	0.025481	0.326548	0.0797995	0.0797995	0.025481	0.180655	0.326548	0.180655
ChatGPT 4.1mini (8/10)	0.00364518	0.0797995	0.0797995	0.0797995	1	0.265584	0.0339446	1	0.00364518	0.265584	0.0123094	0.0339446	0.302788
ChatGPT 4.5 (9/10)	0.00082684	0.025481	0.025481	0.025481	0.265584	1	0.00953816	0.265584	0.000826848	1	0.00308495	0.00953816	0.131776
ChatGPT 5 (4/10)	0.164557	0.326548	0.326548	0.326548	0.0339446	0.00953816	1	0.0339446	0.164557	0.00953816	0.319604	1	0.0887649
Claude 3.7 Sonnet (8/10)	0.00364518	0.0797995	0.0797995	0.0797995	1	0.265584	0.0339446	1	0.00364518	0.265584	0.0123094	0.0339446	0.302788
Claude Opus 4 (2/10)	1	0.0797995	0.0797995	0.0797995	0.00364518	0.000826848	0.164557	0.00364518	1	0.000826848	0.302788	0.164557	0.0123094
Claude Sonnet 4.5 (9/10)	0.00082684	0.025481	0.025481	0.025481	0.265584	1	0.00953816	0.265584	0.000826848	1	0.00308495	0.00953816	0.131776
DeepSeekR1 (3/10)	0.302788	0.180655	0.180655	0.180655	0.0123094	0.00308495	0.319604	0.0123094	0.302788	0.00308495	1	0.319604	0.0368191
Google Gemini 2.5 Flash (4/10)	0.164557	0.326548	0.326548	0.326548	0.0339446	0.00953816	1	0.0339446	0.164557	0.00953816	0.319604	1	0.0887649
Google Gemini 2.5 Pro (7/10)	0.0123094	0.180655	0.180655	0.180655	0.302788	0.131776	0.0887649	0.302788	0.0123094	0.131776	0.0368191	0.0887649	1

Supplementary Figure 15 (Continue). P-values from two sample proportion Z test to show the if two methods yield different performance or not (A) Natural language prompt performance (B) Structure prompt performance

B

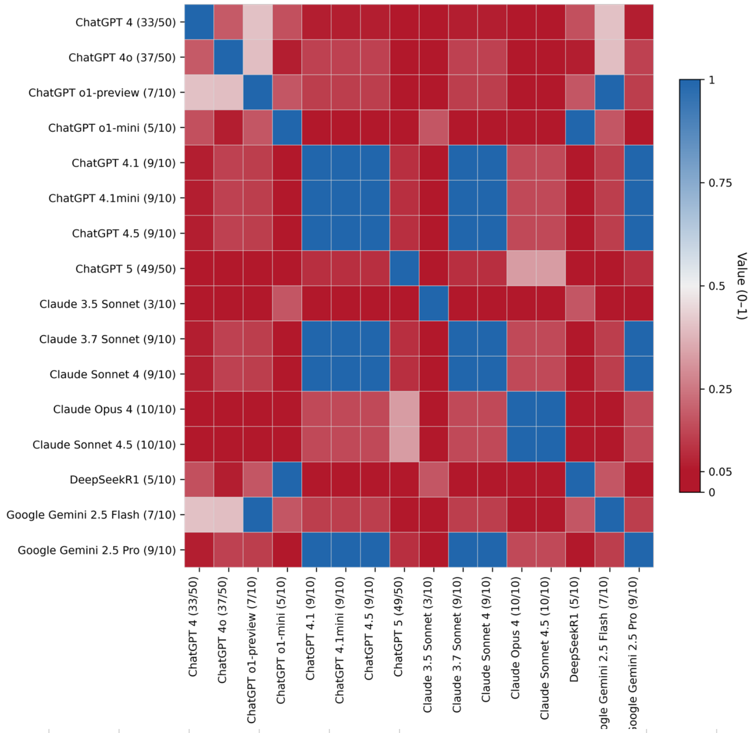

	ChatGPT 4 (33/50)	ChatGPT 4o (37/50)	ChatGPT o1-preview (7/10)	ChatGPT o1-mini (5/10)	ChatGPT 4.1 (9/10)	ChatGPT 4.1mini (9/10)	ChatGPT 4.5 (9/10)	ChatGPT 5 (49/50)	Claude 3.5 Sonnet (3/10)	Claude 3.7 Sonnet (9/10)	Claude Sonnet 4 (9/10)	Claude Opus 4 (10/10)	Claude Sonnet 4.5 (10/10)	DeepSeekR1 (5/10)	Google Gemini 2.5 Flash (7/10)	Google Gemini 2.5 Pro (9/10)
ChatGPT 4 (33/50)	1	0.191367	0.403248	0.168913	0.065285	0.065285	0.065285	1.55926E-05	0.0169474	0.065285	0.065285	0.0146987	0.0146987	0.168913	0.403248	0.065285
ChatGPT 4o (37/50)	0.191367	1	0.397001	0.065285	0.137408	0.137408	0.137408	0.000271755	0.00352536	0.137408	0.137408	0.0342386	0.0342386	0.065285	0.397001	0.137408
ChatGPT o1-preview (7/10)	0.403248	0.397001	1	0.180655	0.131776	0.131776	0.131776	0.000596873	0.0368191	0.131776	0.131776	0.0301446	0.0301446	0.180655	1	0.131776
ChatGPT o1-mini (5/10)	0.168913	0.065285	0.180655	1	0.025481	0.025481	0.025481	1.92981E-06	0.180655	0.025481	0.025481	0.00491164	0.00491164	1	0.180655	0.025481
ChatGPT 4.1 (9/10)	0.065285	0.137408	0.131776	0.025481	1	1	1	0.0991282	0.00308495	1	1	0.152451	0.152451	0.025481	0.131776	1
ChatGPT 4.1mini (9/10)	0.065285	0.137408	0.131776	0.025481	1	1	1	0.0991282	0.00308495	1	1	0.152451	0.152451	0.025481	0.131776	1
ChatGPT 4.5 (9/10)	0.065285	0.137408	0.131776	0.025481	1	1	1	0.0991282	0.00308495	1	1	0.152451	0.152451	0.025481	0.131776	1
ChatGPT 5 (49/50)	1.5593E-05	0.00027176	0.00059687	1.9298E-06	0.0991282	0.0991282	0.0991282	1	3.86E-09	0.0991282	0.0991282	0.325999	0.325999	1.92981E-06	0.00059687	0.0991282
Claude 3.5 Sonnet (3/10)	0.0169474	0.00352536	0.0368191	0.180655	0.00308495	0.00308495	0.00308495	3.86E-09	1	0.00308495	0.00308495	0.00051601	0.00051601	0.180655	0.0368191	0.00308495
Claude 3.7 Sonnet (9/10)	0.065285	0.137408	0.131776	0.025481	1	1	1	0.0991282	0.00308495	1	1	0.152451	0.152451	0.025481	0.131776	1
Claude Sonnet 4 (9/10)	0.065285	0.137408	0.131776	0.025481	1	1	1	0.0991282	0.00308495	1	1	0.152451	0.152451	0.025481	0.131776	1
Claude Opus 4 (10/10)	0.0146987	0.0342386	0.0301446	0.00491164	0.152451	0.152451	0.152451	0.325999	0.00051601	0.152451	0.152451	1	1	0.00491164	0.0301446	0.152451
Claude Sonnet 4.5 (10/10)	0.0146987	0.0342386	0.0301446	0.00491164	0.152451	0.152451	0.152451	0.325999	0.00051601	0.152451	0.152451	1	1	0.00491164	0.0301446	0.152451
DeepSeekR1 (5/10)	0.168913	0.065285	0.180655	1	0.025481	0.025481	0.025481	1.92981E-06	0.180655	0.025481	0.025481	0.00491164	0.00491164	1	0.180655	0.025481
Google Gemini 2.5 Flash (7/10)	0.403248	0.397001	1	0.180655	0.131776	0.131776	0.131776	0.000596873	0.0368191	0.131776	0.131776	0.0301446	0.0301446	0.180655	1	0.131776
Google Gemini 2.5 Pro (9/10)	0.065285	0.137408	0.131776	0.025481	1	1	1	0.0991282	0.00308495	1	1	0.152451	0.152451	0.025481	0.131776	1

Supplementary Figure 16. Modern agentic tools testing (Replit)

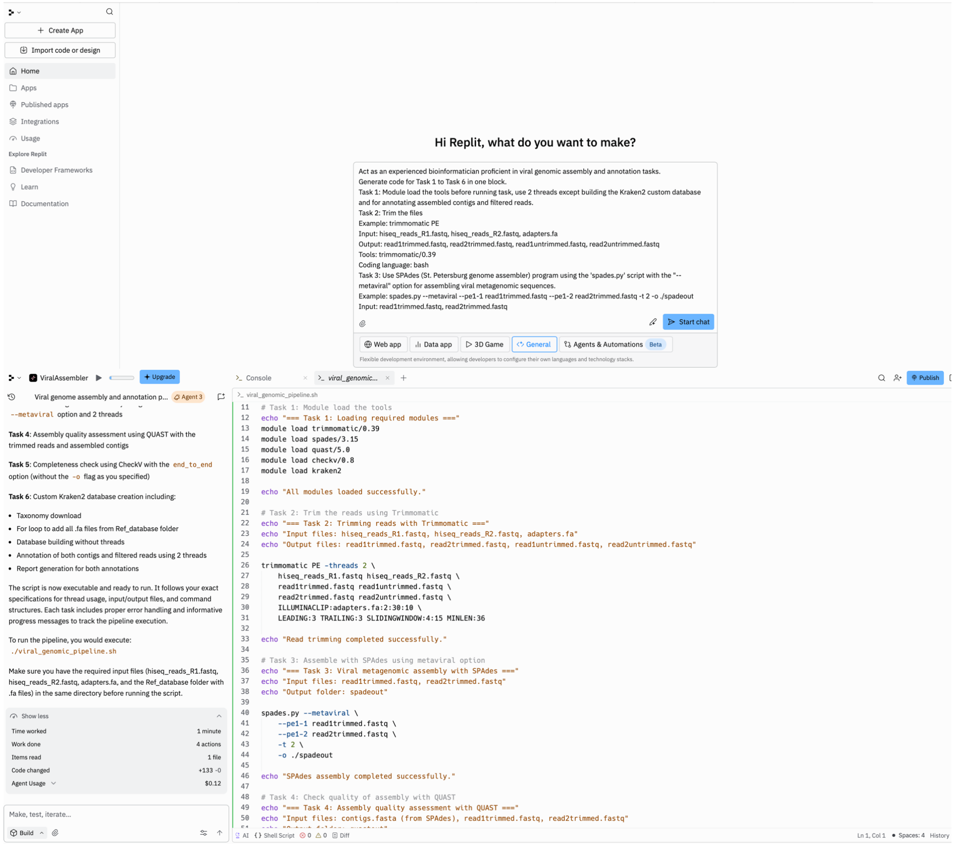


```bash
echo "Output folder: quastout"

quast.py ./spadeout/contigs.fasta \
    -1 read1trimmed.fastq \
    -2 read2trimmed.fastq \
    -o ./quastout

echo "QUAST quality assessment completed successfully."

# Task 5: Check completeness with CheckV
echo "=== Task 5: Assembly completeness check with CheckV ==="
echo "Input file: contigs.fasta (from SPAdes)"
echo "Output folder: checkvout"

checkv end_to_end ./spadeout/contigs.fasta ./checkvout

echo "CheckV completeness assessment completed successfully."

# Task 6: Create custom Kraken2 database and annotate
echo "=== Task 6: Custom Kraken2 database creation and annotation ==="
echo "Input: Ref_database folder with .fa files"
echo "Output: kraken2_db folder (database) and annotation results"

# Create directories for Kraken2 database and results
mkdir -p kraken2_db
mkdir -p kraken2_results

# Download taxonomy information
echo "Downloading taxonomy information..."
kraken2-build --download-taxonomy --db kraken2_db

# Add reference sequences from Ref_database folder
echo "Adding reference sequences to database..."
for fasta_file in Ref_database/*.fa; do
    if [ -f "$fasta_file" ]; then
        echo "Adding $fasta_file to database..."
        kraken2-build --add-to-library "$fasta_file" --db kraken2_db
    fi
done

# Build the database (without using threads as specified)
echo "Building Kraken2 database..."
kraken2-build --build --db kraken2_db

# Annotate assembled contigs with 2 threads and generate report
echo "Annotating assembled contigs..."
kraken2 --db kraken2_db \
    --threads 2 \
    --report kraken2_results/contigs_report.txt \
    --output kraken2_results/contigs_classification.txt \
    ./spadeout/contigs.fasta

# Annotate filtered reads with 2 threads and generate report
echo "Annotating filtered reads..."
kraken2 --db kraken2_db \
    --threads 2 \
    --paired \
    --report kraken2_results/reads_report.txt \
    --output kraken2_results/reads_classification.txt \
    read1trimmed.fastq read2trimmed.fastq

echo "Kraken2 database creation and annotation completed successfully."
```

Supplementary Figure 17. Modern agentic tools testing (Cursor)

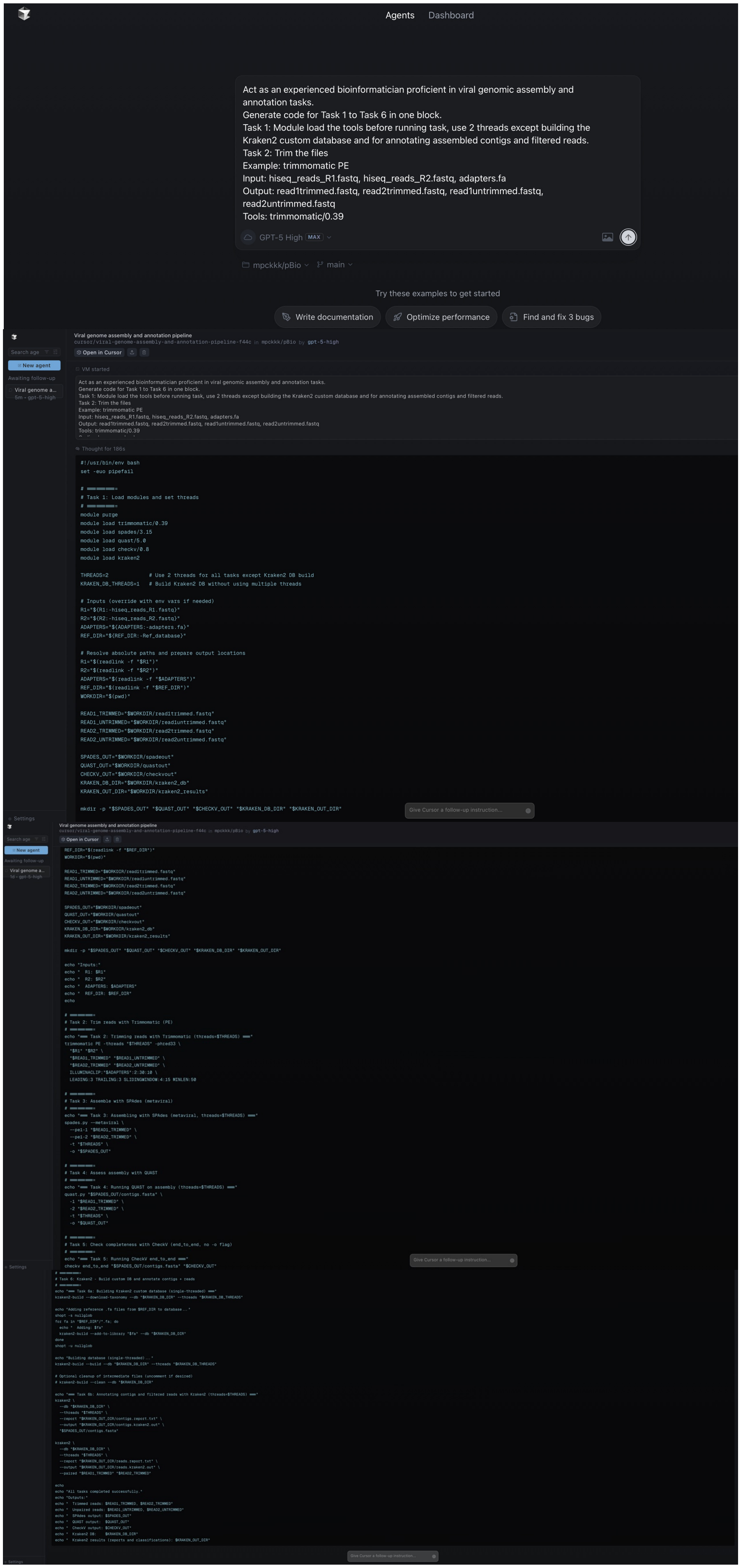

Supplement: vbaf308_Supplementary_Data [file vbaf308_supplementary_data.zip › Supplementary_figures_v14.pdf]
